# PROMISE: a PROspective randomised double-blind parallel group placebo-controlled multicentre trial of faecal MIcrobiota tranSplantation to improve outcomEs in patients with cirrhosis over 24 months—study protocol for a clinical trial

**DOI:** 10.1186/s13063-026-09914-x

**Published:** 2026-07-31

**Authors:** Victoria T. Kronsten, Yevedzo Ntuli, Ellis Kobina Paintsil, Charlotte A. Woodhouse, Thomas H. Tranah, Sue Cheung, Daryl Hagan, Blair Merrick, Angela Cape, Francesca Maher, Kirin Sultana, Desiree Prossomariti, Steven Rodrigues, Christine Masterson, Vanessa Hebditch, Ane Zamalloa, Benjamin H. Mullish, Arjuna Singanayagam, Vishal C. Patel, Mark R. Thursz, PROMISE study group, Victoria T. Kronsten, Yevedzo Ntuli, Charlotte A. Woodhouse, Ane Zamalloa, Benjamin H. Mullish, Arjuna Singanayagam, Vishal C. Patel, Mark R. Thursz, Gavin Wright, Manuel Jasper, Tahir Khan, Syeda Amber Ali, Sarah Fairclough, Bushena Miyesa, Michael Villaruel, Princess Gabiana, Keval Naik, Caroline Fox, Miranda Forsey, Prem Karthikeyan, Jennifer Child, Yvonne Lester, Gautham Appanna, Hannah Donnelly, Alice Lagnado, Robbie Adamson, Joanne Elliot, Gifthy Perez, Rose Fyfe-Beedell, Siby Varghese, Chiaki Shioi, Bindu Mary Jinesh, Shannah O’Sullivan, Hannah Whitehouse, Eunice Akintemi, Matthew Cramp, Glen Marsh, Angela Downing, Gabriella Smallwood, Sasa Topol, Nelly Imbwana, Natasha Wilmshurst, Kelly Bowers, Elanor Haughan, Steven Masson, Juliet Obhiozele, Sarah Hogg, Sheenu Thomas, Lynsey Corless, George Abouda, Kim Dearnley, Jane Rice, Lisa Burton, Julie Gracie, Angie Good, Amy Hicks, Ali Elgadari, Nurun Tania, Ani Kingsbury, Mark McPhail, Michael Ihimekpen, Ruairi Lynch, Damien Leith, Jude Wellens-Mensah, Ann Elliott, Leanne Cosgrove, Abigail Grieve, Paul Johnson, Dina Mansour, Rajan Bhandari, Ann Wilson, Dorothy Carman, Kara Wilshire, Rachael Swann, Laura Davidson, Michael Lumsden, Keziah Lewis, Dominic Rimmer, Faye McMeeken, Shona Perry, Nicole Stoddart, Kirsty Fallon, Lesley Gilmour, Hayley King, Deborah McGlynn, Katherine Burrows, Gemma Duff, Lauren Walker, Alexis Duncan, Guru Aithal, Robert Scott, Adina Olaru, Kimberley Noon, Sophie Cusick, Maria Baffa-Scineli, Amanda Cetroni, Sebastian Wallace, Clare Bradley, Salman Bhatta, Euan Humphreys, Fiona Barrett, Charlotte Barr, Andy Gibson, Valarie Anderson, Safa Al-Shamma, Lindsay Rogers, Fardowsa Ahmed-Timms, Annamaria Wilce, Nicholas Taylor, Andrew Austin, Claire Grant, Maggie Langley, Catherine Addleton, Jisha Joseph, Zeino Zeino, Ankur Srivastava, James Maurice, Lana Ward, Trudie Burge, Louise Jennings, Joseph Delo, Joana Teixeira, Criscel Jan Pelaez, Maria Belini, Amy Curtis, Annie Matthew, Samantha Taylor, Valentia Raspa, Khine Thu, Caroline Gosson, Victoria Comerford, Oluwadamilola Ogunrinde, Kim Sorley, Simon O’ Sullivan, Ian Rowe, Oliver Tavabie, Suzannah Dorey, Arshatisha Joe, Rebecca Bishop, Mehvish Hussain, Yasira Ghaseeta, Madeleine Graydon, Jonathan King, Gareth Hahn, Celia Diaz-Moore, James Neale, Pauline Mercer, Claire Stringer, Sophie Booth, Natasha McDonald, Emma Speake, Rebecca Rielly, Morag Murray, Maria McLaughlin, Sandra Newlands, Janisha Patel, Sofia Pires, Susan Samuel, Faith Panilagao-Bushi, Simon D. Goldenberg, Ben Carter, Lindsey A. Edwards, Debbie L. Shawcross

**Affiliations:** 1 Roger Williams Institute of Liver Studies, School of Immunology and Microbial Sciences, Faculty of Life Sciences and Medicine, King’s College London, Foundation for Liver Research and King’s College Hospital, London, UK; 2 King’s Clinical Trials Unit, King’s College London, London, UK; 3 Centre for Clinical Infection and Diagnostics Research (CIDR), King’s College London and Guy’s & St. Thomas’ NHS Foundation Trust, London, UK; 4 Department of Biostatistics and Health Informatics, Institute of Psychiatry, Psychology and Neuroscience, King’s College London, London, UK; 5 King’s Health Partners Clinical Trials Office, King’s College London, London, UK; 6 Patient and Public Representatives, London, UK; 7 British Liver Trust, Venta Court, Winchester, UK; 8 Department of Hepatology, St Mary’s Hospital, Imperial College Healthcare NHS Trust, London, UK; 9 Division of Digestive Diseases, Department of Metabolism, Digestion and Reproduction, Faculty of Medicine, Imperial College London, London, UK; 10 Infection Clinical Academic Group, City St. George’s University of London, London, UK; 11 Centre for Host Microbiome Interactions, Faculty of Dentistry and Oral Craniofacial Sciences, Guy’s Hospital, Guy’s Tower, King’s College London, London, UK

**Keywords:** Gut microbiome, Faecal microbiota transplantation, Alcohol-related liver disease (ALD), Metabolic dysfunction-associated steatotic liver disease (MASLD), Metabolic dysfunction and alcohol-related liver disease (MetALD), Infection, Antimicrobial resistance

## Abstract

**Background:**

Patients with cirrhosis have reduced gut bacterial diversity and a gut microbiota dominated by pathobionts. These changes, coupled with increased gut permeability and bacterial translocation, increase susceptibility to infection and death. There is also considerable concern that high antimicrobial exposure in the cirrhotic population drives the development of antimicrobial resistance (AMR). We previously performed a randomised feasibility trial of endoscopically administered jejunal faecal microbiota transplant (FMT) slurry derived from stringently screened donors, and showed it to be safe and well-tolerated in patients with cirrhosis (PROFIT Trial: NCT02862249). FMT was associated with reduced enteropathogenic bacteria in the gut, augmented ammonia excretion, ameliorated systemic inflammation, and enhanced innate immune responses to pathogen challenge. The trial was not powered to detect differences in clinical outcomes. The PROMISE study will evaluate the efficacy of lyophilised encapsulated FMT to reduce infection, decompensating events and mortality in patients with cirrhosis secondary to alcohol-related liver disease (ALD), metabolic dysfunction-associated steatotic liver disease (MASLD) cirrhosis and metabolic dysfunction and alcohol-related liver disease (MetALD).

**Methods:**

The PROMISE study is a phase 3 multicentre, randomised, double-blinded, placebo-controlled trial that will evaluate encapsulated lyophilised FMT in 300 participants with ALD, MetALD or MASLD cirrhosis (Model for End-Stage Liver Disease Sodium (MELD-Na) score 8–16). Participants will be randomly allocated (1:1) to receive FMT or matched placebo capsules every 91 days for 21 months, with 24-month follow-up. The primary endpoint is time-to-infection or decompensating event resulting in presentation to the emergency department or hospitalisation.

Secondary endpoints include all hepatic decompensation, all-cause infection, antibiotic usage, incidence of AMR, hospitalisation rates, liver disease severity scores, quality of life scores, Hospital Anxiety and Depression Scale score (HADS), alcohol use, and mortality. Mechanistic endpoints include quantification of plasma bacterial DNA, plasma and faecal cytokines/biomarkers, plasma and faecal metabolome, faecal proteome, faecal microbiome composition and diversity (including resistome) and monocyte and mucosal-associated invariant T (MAIT) cell frequency, phenotype and function. Recruitment commenced in June 2023. Results will be disseminated via peer-reviewed journals, international conferences and patient support groups.

**Discussion:**

The PROMISE study will assess the efficacy and evaluate the mechanisms of action of FMT in patients with ALD, MASLD and MetALD cirrhosis. FMT may provide an alternative non-antibiotic treatment for these patients through ecological reconstitution of microbial balance.

**Trial registration:**

ISRCTN, ISRCTN17863382. Registered on 25th March 2022, https://www.isrctn.com/ISRCTNISRCTN17863382. ClinicalTrials.gov NCT06461208. Registered on 4th June 2024, https://clinicaltrials.gov/study/NCT06461208#study-overview.

**Supplementary Information:**

The online version contains supplementary material available at https://doi.org/10.1186/s13063-026-09914-x.

## Background and trial rationale

There is an evolving crisis of chronic liver disease (CLD) in the UK, with the prevalence and mortality increasing exponentially. It is estimated that 10–20% of the UK population are at risk of developing CLD [1]. Advanced CLD, known as cirrhosis, accounts for 2 million deaths per year and 4% of global mortality [2].

Infection is common in cirrhosis and occurs in up to 40% of hospital admissions [3]. Infection frequently precipitates complications including hepatic encephalopathy, gastrointestinal bleeding, acute kidney injury, acute-on-chronic liver failure (ACLF) and multi-organ failure (MOF), thus carrying a high mortality [4]. Overt systemic inflammation with concurrent functional immunodeficiency is exhibited in cirrhosis, a phenomenon termed cirrhosis-associated immune dysfunction (CAID), resulting in an increased propensity to infection and higher likelihood of resultant complications [5]. Furthermore, drug-resistant infections pose a significant challenge in cirrhosis [6], with their prevalence increasing from 29 to 38% between 2011 and 2018 [7]. A recent meta-analysis further demonstrated that 31% of bacteraemia cases in cirrhosis were caused by multidrug-resistant bacterial infections [6]. Current strategies to prevent infection rely heavily on long-term antibiotic prophylaxis (e.g. norfloxacin, rifaximin), which can reduce infection rates but contribute to antimicrobial resistance (AMR).

Patients with cirrhosis, especially alcohol-related liver disease (ALD) and metabolic dysfunction-associated fatty liver disease (MASLD), have a microbial imbalance with reduced gut bacterial diversity and a microbiome dominated with pathobionts that can drive barrier damage and inflammation, and in some instances translocate from the gut to the systemic circulation, resulting in endotoxaemia and systemic inflammation, which significantly drive CAID [4]. Reported pathobionts in cirrhosis include *Escherichia coli* and other *Enterobacterales*, *Staphylococcus aureus*, pathogenic *Clostridium* species, *Enterococcus*, and certain *Prevotella* strains [6, 8–10], which can drive barrier damage and systemic inflammation [11–13].

Manipulation of the gut microbiome in individuals with cirrhosis and hepatic encephalopathy with the non-absorbable antibiotic rifaximin has proven beneficial, with significant reductions in hospitalisation and 30-day readmissions [14]. In the RIFSYS trial, rifaximin reduced markers of gut-derived systemic inflammation and abundance of pathobionts in both the faecal and salivary compartments and reduced infections [15]. However, considerable concern remains regarding the long-term effect of antibiotic prescriptions on AMR [16, 17]. Rifaximin resistance in E. coli exceeds 60% in patients on chronic therapy [18]. This underscores the urgent need for non-antibiotic alternatives.

Faecal microbiota transplantation (FMT) is an established treatment to modify the gut microbiome and has been shown to be safe [19], cost-effective [20], and efficacious in disease states resulting from gut dysbiosis [21], including recurrent *Clostridioides difficile* infection [22]. Bajaj et al. (2017) demonstrated in an open-label phase 1 pilot study of 10 patients treated with rectally instilled FMT from a single stool donor, that FMT is safe and potentially efficacious in treating hepatic encephalopathy [23]. The THEMATIC trial [24], a phase II randomised, placebo-controlled trial exploring the use of capsule and enema FMT in patients with cirrhosis and hepatic encephalopathy treated with lactulose and rifaximin, further demonstrated safety and tolerability, regardless of route of delivery, with the highest rates of hepatic encephalopathy recurrence in the placebo group [24]. Further work has revealed that FMT may also increase gut-microbial richness and reduce carriage of enteric pathogens, intestinal barrier damage, and systemic inflammation [21].

We recently demonstrated, through our feasibility study PROFIT (PROspective, randomised placebo-controlled feasibility trial of Faecal mIcrobiota Transplantation in cirrhosis NCT02862249) [25], that FMT is safe and well-tolerated in cirrhotic patients [26]. Either FMT suspension (manufactured from a minimum of 50 g of stool donated by carefully screened donors) or placebo (0.9% normal saline), allocated in a 3:1 ratio in a single-blinded fashion, was delivered by upper gastrointestinal endoscopy into the jejunum, following bowel lavage, in patients with advanced cirrhosis (Model for End-Stage Liver Disease (MELD) [27] score 10–16) who were not receiving antibiotics. This trial was not powered to detect differences in clinical outcomes. However, a significant reduction in plasma ammonia between baseline and day-30 was observed in those treated with FMT [26].

The beneficial effects of FMT diminished by approximately 90 days post-administration, with systemic ammonia levels trending back toward baseline, indicating a single endoscopic dose provides only short- to medium-term ecological and metabolic benefit. During the trial, it also became clear that not all eligible patients were willing to undergo an invasive endoscopy for FMT administration, and expressed a preference for oral ingestion of FMT. This was confirmed by patient and public involvement (PPI) workshops. Bajaj et al. (2020) published a phase 1 study, demonstrating that oral FMT capsules are safe, well-tolerated, and improved cognitive performance in 10 patients with cirrhosis and recurrent hepatic encephalopathy [28]. Furthermore, a recent feasibility randomised control trial (RCT), investigating the use of oral encapsulated lyophilised FMT to eradicate gastro-intestinal carriage of drug-resistant organisms, demonstrated satisfactory donor-derived engraftment [29].

Larger studies are now required to determine the efficacy of FMT in patients with cirrhosis, in a large, well-characterised cohort. The PROMISE trial is a phase 3 multicentre, randomised, double-blinded, placebo-controlled trial that will evaluate encapsulated, lyophilised FMT versus matched placebo for 2 years in patients with ALD, MASLD and MetALD cirrhosis. Matched placebo is justifiable as a comparator arm as there is no available alternative specific therapy to reduce mortality and improve outcomes in cirrhosis. The use of encapsulated FMT reduces risk further by removing the possibility of endoscopy-related adverse events. We hypothesise that encapsulated FMT will reduce infection, decompensating events and mortality in this patient cohort.

## Methods and analysis

### Objectives

Primary objective:

1. To evaluate the efficacy of encapsulated FMT to reduce the susceptibility to infection or decompensating events in patients with cirrhosis, measured by the time to first infection or decompensation resulting in presentation to the emergency department (ED) or hospitalisation.

Secondary objectives:

1. To evaluate the efficacy of encapsulated FMT to reduce non-acute decompensating episodes of cirrhosis including new-onset or worsening grade of ascites and covert hepatic encephalopathy (West Haven criteria grade 0–1) [30].
2. To evaluate the impact of encapsulated FMT on patient outcomes including all-cause hospitalisation, disease progression (including development of acute-on-chronic liver failure), quality of life, mental health, alcohol use and survival.
3. To assess the impact of FMT on antibiotic usage and loss/reduction of AMR genes.
4. To evaluate the safety of encapsulated FMT.

Mechanistic objectives:

1. To determine whether FMT increases the diversity of the gut microbiome in cirrhosis.
2. To determine whether FMT reduces bacterial translocation and endotoxaemia.
3. To evaluate whether FMT improves CAID and reduces systemic inflammation.

Primary endpoint:

1. Time to first infection resulting in presentation to the ED or hospitalisation with a confirmed infection (See Table 3 for trial definition of infection) or time to decompensating event (see Table 4 for trial definition of decompensating event) resulting in presentation to the ED or hospitalisation.

Secondary endpoints (over a 24-month follow-up period):

1. Time to first infection resulting in hospitalisation.
2. Incidence of any decompensating episode of cirrhosis, including hepatic encephalopathy, new-onset or worsening ascites or variceal bleeding.
3. Incidence of all-cause infection, including infections not resulting in hospitalisation.
4. Progression to ACLF (the development of one or more extrahepatic organ failures) [31].
5. Infection rates and antibiotic usage following randomisation.
6. Incidence of infections or colonisation with antimicrobial-resistant organisms including skin and nose colonisation with methicillin-resistant *Staphylococcus aureus* (MRSA), vancomycin-resistant *Enterococci* (VRE), extended-spectrum beta-lactamase-producing *Enterobacterales* (ESBL), fluoroquinolone-resistant Gram-negative and carbapenem-resistant *Enterobacterales* [32].
7. Hospitalisation rates (liver-related and all-cause) including the length of stay and admission to high dependency/intensive care.
8. Change in liver disease severity scores (Child–Pugh, MELD-Na, United Kingdom Model for End-Stage Liver Disease (UKELD)) at 3, 6, 12 and 24 months.
9. Change in quality of life (EQ-5D-5L score) at 3, 6, 12 and 24 months [33].
10. Change in depression and anxiety scores (Hospital Anxiety and Depression Scale (HADS)) at 3, 6, 12 and 24 months [34].
11. All-cause mortality and liver-related mortality.
12. Change in alcohol use in patients enrolled with ALD and MetALD as assessed by the extended item alcohol-use disorders identification test (AUDIT score) [35] and urinary ethyl glucuronide/ethyl sulphate levels (if taken as part of the standard of care).
13. Safety based on safety assessments including physical examination, clinical laboratory evaluation, vital signs and reported as adverse events (AEs) or serious adverse events (SAEs).

Mechanistic endpoints:

1. Matched faecal and plasma biomarkers of gut barrier integrity (including D-lactate, fatty acid-binding protein 2 and ammonia) measured by enzyme-linked immunosorbent assay (ELISA).
2. Faecal proteases as markers of DNA replication, tissue remodelling and immune response as a proxy of gut mucosal barrier integrity.
3. Matched plasma and faecal cytokine profiles using a combination of ELISA and electrochemiluminescence using the mesoscale discovery platform (MSD) (tumour necrosis factor-alpha, interferon-gamma, interleukin (IL)−1-beta, IL-6, IL-8, IL-10, IL-17A, IL-17E, IL-17F, IL-21 and IL-22).
4. Plasma, faecal and urinary metabonomics including short-chain fatty acids, tryptophan metabolites and bile acids.
5. Faecal proteomics.
6. Matched faecal and blood microbiome composition (including mycome, virome and phagome) and microbial diversity by metagenomic sequencing.
7. Faecal PCR for pathogenic species of interest, including copy number of DNA of cytolysin positive and negative *Enterococcus faecalis*, enteropathogenic *Escherichia*. *coli* and *Candida albicans*.
8. Analysis of the presence and dynamics of microbial resistome (ensemble of genes encoding AMR in a given microbiome).
9. Assessment of circulating innate immune profile from stored peripheral blood mononuclear cells (PBMCs), by assessing monocyte cell number, phenotype and function.
10. Assessment of mucosal-associated invariant T (MAIT) cell (isolated from PBMCs) frequency, phenotype and function.
11. Change in lipid profile (total cholesterol, triglycerides, HDL cholesterol and LDL cholesterol) and glycated haemoglobin (HbA1c) in those with MASLD and MetALD cirrhosis.
12. To investigate the impact of dietary habits on participants’ gut microbiome composition, by integrating faecal microbiome sequencing with metabolomic profiling and dietary intake data from baseline dietary questionnaire (Supplementary Materials).

### Rationale for changing the primary objective and primary end-point

The primary endpoint for the PROMISE trial was initially time to first infection resulting in hospitalisation.

Following discussion and approval from trial oversight committees (Trial Monitoring Group (TMG), Data Monitoring Committee (DMC) and Trial Steering Committee (TSC)) and PPI members, an amendment was submitted and the primary outcome was changed from time to first infection resulting in hospitalisation, to a composite primary endpoint, of time to first infection and decompensation resulting in presentation to the ED or hospitalisation. There is emerging evidence that the gut microbiome plays a key role in decompensating events [36–38] and recent research has suggested that FMT may have a potential therapeutic effect in this setting [24]. The use of ambulatory emergency care, including day-case paracentesis units, has rapidly increased in the UK in recent years [39]. Therefore, the primary endpoint was updated to include participants who present to ED with a confirmed infection or new decompensation.

Change to primary endpoint was approved by the TSC, DMC, MHRA, research ethics committee (REC) and funder and disseminated to all collaborators and study sites via email.

### Trial design

The SPIRIT reporting guidelines were used to write this protocol [40] (SPIRIT checklist in Supplementary Materials).

**Fig. 1:**
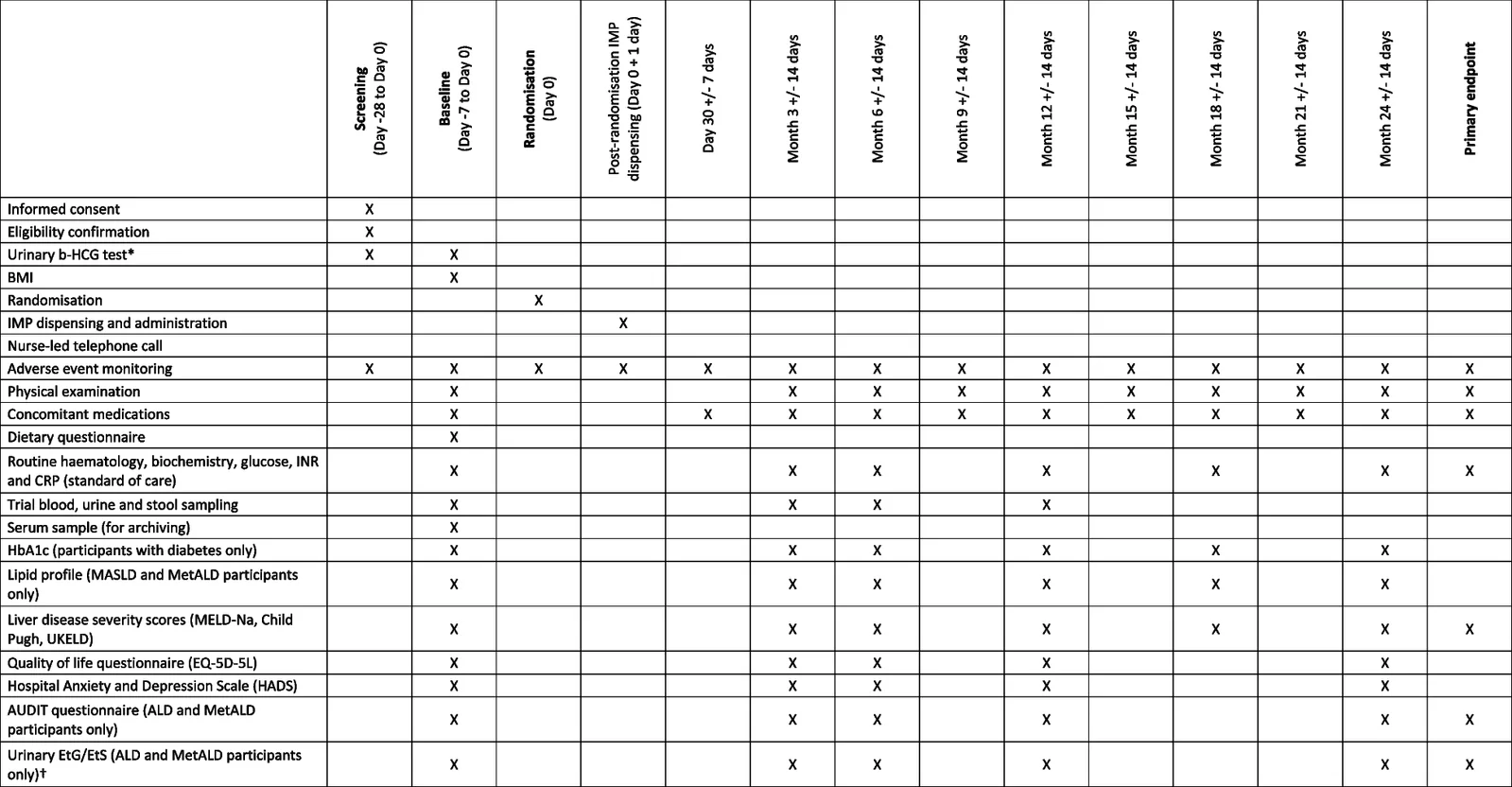
SPIRIT figure: study schedule of events. * In females of childbearing potential. When screening and baseline visits are performed on the same day, the Urinary β-HCG test will only need to be performed once for eligibility confirmation. † Only performed if this is a part of the participant’s routine standard of care. Abbreviations: AUDIT, alcohol use disorders identification test; β-HCG, beta-human chorionic gonadotropin; BMI, body mass index; CRP, C-reactive protein; EtG, ethylglucoronide; EtS ethyl sulphate; HADS, Hospital Anxiety and Depression Scale; HbA1c, glycated haemoglobin; IMP, investigational medicinal product; INR, international normalised ratio; MASLD, metabolic dysfunction-associated steatotic liver disease; MetALD, metabolic dysfunction and alcohol-related liver disease; MELD-Na, model for end-stage liver disease sodium; UKELD, United Kingdom model for end-stage liver disease

This is a phase 3 UK multicentre, randomised, double-blinded, parallel group placebo-controlled trial evaluating encapsulated FMT versus matched placebo with two-year follow-up. Placebo will be used as a comparator arm as there is no available alternative specific therapy to reduce mortality and improve outcome in cirrhosis. Participants will be randomised 1:1 to FMT or placebo. Participants will receive the investigational medicinal product (IMP) every 91 days ± 14 days for 21 months and will be evaluated at baseline before the IMP is administered and at 30 days (nurse-led telephone interview), and then every 3 months for 24 months. Blood, stool and urine will be collected at baseline, 3 months, 6 months and 12 months (see Fig. 1: SPIRIT figure: study schedule of events).

### Selection of participants

**Fig. 2:**
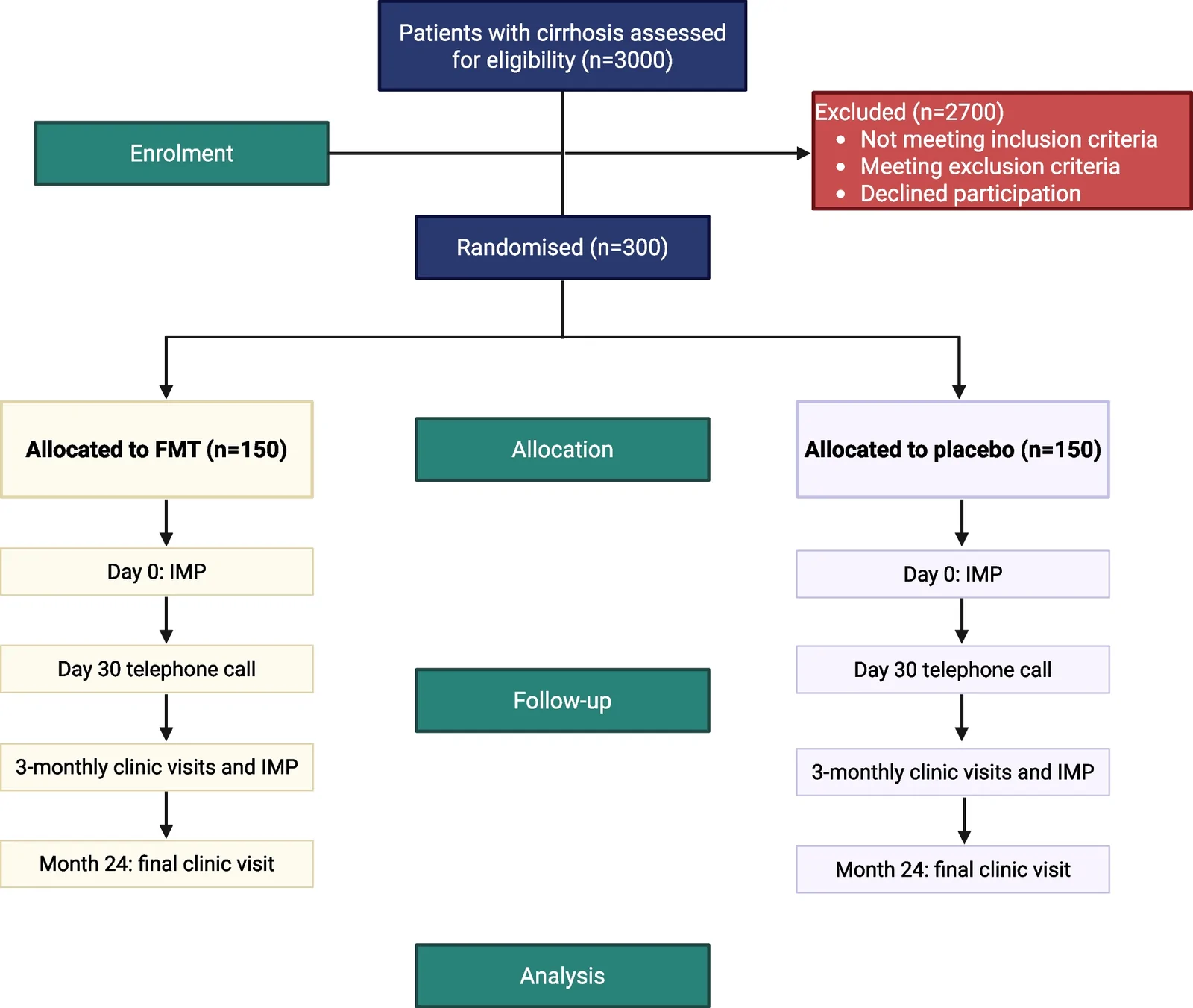
Consort trial participant flow diagram. Abbreviations: IMP, investigational medicinal product

Patients will be recruited from gastroenterology/hepatology outpatient clinics in 20 UK centres serving populations where rates of ALD, MASLD and MetALD are amongst the highest. A list of study sites can be found in the Acknowledgements section. Eligibility criteria are shown in Table 1. Anticipated recruitment is outlined in Fig. 2: consort trial participant patient flow diagram. Table 2 describes the expected demographic characteristics of trial participants.

**Table 1:** Inclusion and exclusion criteria

**Inclusion criteria**
Age 18 or over
Confirmed ALD, MASLD or MetALD cirrhosis based on clinical, radiological and/or histological criteria
MELD-Na score 8–16* [41]
All patients must be consuming ≤ 20 g of alcohol per day (on average) †
Patients must be deemed to have the capacity to provide written informed consent to participate
**Exclusion criteria**
Moderate, severe or life-threatening food allergy‡
Pregnancy or planned pregnancy§
Breastfeeding
Patients treated for acute variceal bleeding, infection, overt hepatic encephalopathy or ACLF within 14 days prior to randomisation
Active alcohol consumption of > 20 g/day
Previous liver transplant
Patients with inflammatory bowel disease
Patients with coeliac disease
Patients with a history of prior gastrointestinal resection or surgery that could change their gut microbiome or result in bacterial overgrowth, e.g. gastric bypass
Active malignancy including hepatocellular carcinoma
Patients with an expected life expectancy < 6 months or listed for liver transplantation
HIV, hepatitis B or C infection (patients who have undetectable HBV DNA/HCV RNA can be recruited)
Received antibiotics or probiotics (excluding foodstuffs containing ‘live bacteria’ such as live yoghurts, kefir, fermented vegetables such as sauerkraut/kombucha or cheese) in the 7 days prior to randomisation
Swallowing disorder, oral-motor dysfunction or likely inability/unwillingness to ingest study medication
Patients who have received another investigational drug or device within 4 months prior to randomisation
Patients, who in the opinion of the PI, have a medical condition, or other relevant psychological, familial, or social factor that may jeopardise their health, compliance, or influence the trial integrity in any way

*Abbreviations*: *ALD* alcohol-related liver disease, *ACLF* acute-on-chronic liver failure, *DNA* deoxyribonucleic acid, *HIV* human immunodeficiency virus, *MASLD* metabolic dysfunction-associated steatotic liver disease, *MetALD* metabolic dysfunction and alcohol-related liver disease, *MELD-Na* model for end-stage liver disease sodium, *PI* principal investigator, *RNA* ribonucleic acid

^*^MELD-Na score calculated in the previous 3 months can be used to confirm eligibility at the screening visit

^†^1 unit of alcohol contains 10 mL or 8 g of alcohol

^‡^Moderate, severe or life-threatening food allergy as defined by evidence of systemic involvement with any respiratory symptoms, or gastrointestinal symptoms that have ever required treatment or hospitalisation, or the presence of hypotension. Moderate to severe also includes cutaneous or mucosal symptoms that have ever required treatment or hospitalisation [42, 43]

^§^Urine testing will be performed at screening to rule out pregnancy in females of childbearing potential

**Table 2:** Non-clinical participant characteristics

Characteristic	Participants expected to be recruited
Age	The highest prevalence rate of cirrhosis is observed in those aged 50 to 69 years, with mortality peaking at 61 in men and 62 in women in the UK [44, 45]
Sex	Males and females will be included. Prevalence of cirrhosis, including ALD cirrhosis, is higher in males, and mortality from cirrhosis is higher in males than females [46, 47]
Gender	Men and women will be included, including transgender men and women
Ethnicity	More white individuals are diagnosed with ALD cirrhosis in the UK [47]. The trial will aim for community levels of ethnic diversity. The PIS has been translated into additional languages to reflect the local demographics of the study sites (Polish, Punjab, Spanish, Urdu and Welsh)
Socioeconomic status	Data has shown higher mortality in individuals with cirrhosis from more deprived areas [48] The trial will aim for broad participation from least to most deprived
Geographic location	The annual incidence of ALD cirrhosis is higher in northern areas of England [47]. The trial has 20 recruiting sites, including in northern areas of England and Scotland, and will aim for broad representation from all geographic locations, including rural areas

*Abbreviations*: *ALD* alcohol-related liver disease, *PIS* patient information sheet, *UK* United Kingdom

### Recruitment and informed consent

All participants will be asked to provide written informed consent (Supplementary Materials) after having read the patient information sheet (PIS) (Supplementary Materials) and having had the opportunity to discuss the trial with the clinician, their family and friends. Written informed consent will be obtained by medical staff delegated at each recruiting site having received protocol-specific training. Patients must be deemed to have the capacity to consent to enrol in the study. If participants lose capacity during the trial they will be immediately withdrawn from the trial.

### Study intervention

#### Investigational medicinal product (IMP)

FMT for this trial will be manufactured in an FMT laboratory at St Thomas’ Hospital in a lyophilised, encapsulated form in accordance with current Good Manufacturing Practice (cGMP) principles and under Manufacturing Authorisation for an IMP from the Medicines and Healthcare Products Regulatory Agency (MHRA). FMT donors are carefully screened volunteers who meet stringent health and eligibility criteria, including age between 18 and 60 years and body mass index between 18 and 30 kg/m^2^. Donors must not be taking any regular medication nor have had any antibiotics in the previous 3 months. Donors provide written informed consent and subsequently undergo questionnaire screening for risk factors (Supplementary Materials) and blood and stool testing for a range of infectious agents and in accordance with national guidelines published jointly by the British Society of Gastroenterology and the Healthcare Infection Society in the UK [21, 22, 49]. Full donor enrolment details and screening protocol are included in supplementary materials (Supplementary Table 1).

FMT contains human faeces and 0.9% sodium chloride and 5% trehalose (cryoprotectant) as excipients. A minimum of 50 g of faeces from a single donor is used as starting material for manufacture. Following filtration and centrifugation to remove undigested food and most of the water content, a fixed weight of faecal microbiota is frozen and lyophilised to generate a fine powder, which is encapsulated in five size 0 delayed-release methylcellulose capsules (DRcaps™, Capsugel®, Livingston, UK). Viable bacterial counts are stable for at least 6 months when stored at − 20 °C and 8 months at − 80 °C [49, 50], or as per the defined period stated in the latest Investigational Medicinal Product Dossier (IMPD). The capsules are coloured Swedish orange which is opaque to preserve the blinding FMT material, which is fully traceable from donor to recipient. Aliquots of donor stool will be kept for 30 years to allow for future testing if required.

Placebo will be manufactured by the Royal Free London NHS Foundation Trust, Clinical Trial Manufacturing and Supplies Unit, and will be provided as 5 size 0, Swedish orange microcrystalline methylcellulose capsules so as to appear identical in appearance to the FMT capsules.

The site pharmacy teams will be unblinded to a patient’s treatment allocation. The trial centre pharmacy will dispense the correct treatment arm in accordance with the unblinded randomisation notification upon receipt of a trial-specific prescription. The FMT capsules reach room temperature rapidly once removed from the freezer. FMT capsules that have been stored at room temperature for 6 h or more will not be administered to patients. The capsules are designed to retain integrity in the low pH of the stomach, releasing their contents only at the intestinal pH of 6.8.

#### Dosing regimen

Participants will receive five capsules of the IMP to be taken by mouth and swallowed every 91 days ± 14 days for a total of 21 months. Participants are recommended to fast (clear fluids only) for 4-h prior to treatment. They will be requested to ingest all capsules within 30 min and are permitted unlimited water or squash. The last treatment will be administered at the 21-month visit.

#### Participant compliance

Participants will take their IMP at their hospital outpatient visits so adherence will be directly observed. The total number of capsules taken by the patient will be recorded.

Trial participants who are only able to swallow 1 or more of the capsules but not all 5 will continue to be followed up as per the study schedule but this will be recorded in the eCRF.

Participants who are unable or are unwilling to ingest the IMP will be withdrawn from the trial.

#### Concomitant medication

Participants must not have received antibiotics within 7 days prior to randomisation. If oral or intravenous antibiotics are later indicated during the course of the study for the treatment of suspected or confirmed infection, or rifaximin for the treatment of recurrent overt hepatic encephalopathy, then these will be permitted to be given as standard of care.

If a participant has been treated with antibiotics for an acute infection following commencement of IMP which did not necessitate hospital admission, repeat dosing will be delayed until 7 days after completing the antibiotic course. If participants have been commenced on long-term prophylactic antibiotics as part of their standard clinical care that will continue for the remaining duration of the trial, then patients will be permitted to receive their IMP as per dosing schedule. The development of infection or decompensation resulting in hospitalisation or presentation to the ED will meet the primary endpoint and the participant will no longer receive further IMP doses, although they will continue to be followed-up for the duration of the trial. Patients who undergo liver transplantation will no longer receive IMP due to the lack of robust safety data of FMT administration in immunocompromised patients post liver transplantation.

### Trial procedures

#### Evaluations during and after treatment

Following identification, all patients will attend a screening visit and sign the consent form. A urinary pregnancy test will be performed for all females of childbearing potential. If eligibility is then confirmed, participants will attend for a baseline visit, which includes a focused medical history, concomitant medication review, physical examination, calculation of body mass index (BMI) and deprivation index. Participants will complete a dietary questionnaire, a quality-of-life questionnaire (EQ-5D-5L) [33], and the HADS questionnaire [34]. Participants with ALD or MetALD cirrhosis will complete the AUDIT questionnaire (questions 4–10 modified to capture alcohol use over past 6 months) [35]. Standard of care blood tests will be performed (Supplementary Materials, Supplementary Table 2) allowing calculation of liver disease severity scores, and baseline samples of stool, urine and blood will be collected as per protocol (Supplementary Materials, Supplementary Table 3). The result of a urinary ethyl glucuronide/ethyl sulphate (EtG/EtS) test, if performed as standard of care, will be recorded.

IMP will then be dispensed following randomisation and administered by an unblinded research nurse, who is only delegated this role. The IMP packs cannot be fully blinded due to a difference in the temperature storage conditions, expiry dates and batch number configurations for the FMT and placebo products.

Participants will receive a safety telephone call at day 30 (± 7 days) to check for adverse events and record concomitant medications.

Participants will then be evaluated before IMP is administered at 3, 6, 9, 12, 15, 18, 21 months and at the final 24-month visit. No IMP is administered at the 24-month visit. At each visit physical examination, concomitant medications and adverse events will be recorded, and the investigator will determine if the participant is fit for IMP administration. The EQ-5D-5L, HADS and AUDIT questionnaire (where applicable) will be repeated at 3, 6, 12 and 24 months. Standard of care blood tests and liver disease severity scores will be performed at 3, 6, 12, 18 and 24 months, and urinary ETG/ETS result recorded if performed. Study-specific blood, urine and stool will be collected at 3, 6 and 12 months.

After completion of the trial, standard of care for each patient will continue.

#### Randomisation

Randomisation will be undertaken by the local research team at each site once written informed consent has been obtained, eligibility confirmed, and baseline data collected. The personnel who enrol and assign participants to the interventions will not have access to the random allocation sequence.

A web-based system has been designed, using the bespoke King’s Clinical Trials Unit (KCTU) randomisation system. Randomisation will be at the level of the individual using the method of block randomisation with varying block sizes, stratified by site.

#### Blinding

All participants, investigators and outcome assessors will be fully blinded throughout the trial. The site pharmacy trials team and the research nurse administering the intervention will be unblinded to participants’ treatment allocation. The senior statistician will also be fully blinded, but the junior statistician will be unblinded from the 1 st DMC meeting with data onwards.

#### Unblinding

Each randomised participant will be provided with a card containing trial team contact numbers and emergency contact details. Participants will be asked to carry this card with them at all times whilst participating in the trial.

Investigators will only break the blind when information about the participant’s trial treatment is necessary to instigate appropriate medical management where stopping the blinded IMP is not sufficient. If there is no alternative, a 24-h telephone-based emergency code break service, Emergency Scientific Medical Services (eSMS), will be used.

#### Primary endpoint

Site PIs will determine if participants have met the primary endpoint, with presentation to the ED or hospitalisation for infection or decompensating event, as defined in Tables 3 and 4, respectively. Participants will be reviewed for adverse event monitoring and concomitant medications recorded in the eCRF. A physical examination will be performed or the findings recorded from the medical records if the patient is admitted to a hospital other than their recruiting trial centre. Results of the standard of care blood tests taken on admission will be documented. Blood, urine, stool, sputum, skin and/or ascitic culture results, and report of chest radiograph, as applicable, will be recorded.

**Table 3:** Trial definition of infection

Lower respiratory tract infection	New pulmonary infiltrate on chest x-ray (CXR) in the presence of at least one respiratory symptom (cough, sputum production, dyspnoea, pleuritic pain) or one sign of infection (core body temperature > 38 °C or less than 36 °C, shivering, or leukocyte count > 10,000/mm^3^. This includes the presence of respiratory tract viral infections, including COVID-19, contracted in the community resulting in a hospital admission with a positive test on viral PCR. Viral infections contracted whilst in hospital will not qualify as meeting primary endpoint*
Urinary tract infection	Urine white blood cell > 15/high-power field (or equivalent) with either positive urine Gram stain (or cytometry) or culture†
Catheter-associated urinary tract infection	Evidence of an organism grown on urine culture† or positive urine gram stain (or cytometry), plus symptoms to suggest systemic infection such as suprapubic pain, pyuria, fever > 38 °C, signs of sepsis
Spontaneous bacterial peritonitis	Ascitic fluid absolute neutrophil count > 250 cells/mm^3^ and/or positive ascitic culture†
Gastrointestinal infection	Positive stool culture†/PCR for a pathogen typically associated with gastrointestinal infection, including the presence of Clostridioides difficile infection/toxin if suspected
Cellulitis or soft tissue infection	Localised area of pain tenderness, swelling, erythema and heat, with a positive swab/tissue sample on culture or requiring intravenous antibiotics with evidence of improvement after treatment
Bone infection/osteomyelitis	Positive microbial culture† and/or PCR of a bacterial organism from biopsy of bone
Bacteraemia	The presence of bacteraemia with a positive line tip or peripheral blood cultures†
Central nervous system (meningitis or encephalitis)	Positive cerebrospinal fluid microscopy, culture† and/or PCR with a pathogen typically associated with meningitis/encephalitis. This includes viral, bacterial and fungal infection
Joint infections	Localised area of pain, tenderness, swelling, erythema and heat, with joint fluid analysis compatible with infection and positive microbial culture† and/or PCR of a bacterial organism
Intra-abdominal infections	Symptoms of abdominal pain, tenderness or jaundice (to suggest cholangitis) combined with core body temperature > 38 °C or less than 36 °C, rigours or leukocyte count > 10,000/mm^3^ and confirmatory findings on imaging to suggest an infective source such as cholecystitis or intra-abdominal abscess
Sepsis of unknown source	Sequential organ failure assessment (SOFA) score of ≥ 2 from baseline [51] to indicate new organ dysfunction AND clinical suspicion of infection with no identified source despite thorough screening with imaging (CXR and Computed Tomography (CT) scan), urine culture, blood or line tip cultures, ascitic tap, stool culture or wound swab

^*^The diagnosis of incidental viral infection, including COVID-19, contracted in the community or whilst in hospital, will not qualify as meeting primary endpoint

^†^Culture refers to both bacterial and fungal infection

**Table 4:** Trial definition of decompensating event [52]

New-onset moderate or large volume ascites requiring diuretic therapy and/or paracentesis*
Variceal bleeding confirmed following emergency endoscopy or CT angiography suggestive of portal hypertensive bleeding elsewhere in the gastrointestinal tract
Overt hepatic encephalopathy (West Haven criteria grade 2–4) [30]

^*^Presentation to day case paracentesis unit with new-onset moderate or large volume ascites will also qualify as meeting primary endpoint. *Abbreviations*: *CT* computed tomography

For those participants with ALD or MetALD cirrhosis, the AUDIT questionnaire will be completed, which may be performed up to 14 days following discharge.

Participants who meet the primary end-point will no longer receive IMP, nor attend the clinic for follow-up trial visits, nor have further samples collected. If they are willing to continue to provide trial-specific data, this will be collected via 3-monthly telephone visits or from routine clinical practice visits (notes-based follow-up). 

### Participant withdrawal

Trial participants have the right to withdraw from trial participation at any time and for any reason, without penalty. Withdrawn participants will not be replaced.

### Safety and risk assessment

#### Procedures for recording and reporting adverse events

The Medicines for Human Use (Clinical Trials) Regulations 2004 and Amended Regulations 2006 definitions for adverse events (AE), adverse reactions (AR), unexpected adverse reactions (UAR), serious adverse events (SAE), serious adverse reactions (SAR), suspected unexpected serious adverse reactions (SUSAR), important medical events (IME), including pregnancy, will be strictly followed, and such occurrences reported immediately (and no later than 24 h) by the site Principal Investigate (PI) or delegate to the King’s Health Partners Clinical Trials Office (KHP-CTO) and Chief Investigator (CI). Consideration for liver transplantation (referral and listing) will also be deemed an IME and require expedited reporting to the KHP-CTO.

All investigators in the study will be provided with full details of possible adverse events that may result from the trial medication. The KHP-CTO will report SUSARs to the regulatory authorities (MHRA). The CI will report to the relevant ethics committee.

#### Safety parameters

The following safety parameters will be recorded in the eCRF at the scheduled visits as per Fig. 1:

- Adverse events.
- Episodes of infection not requiring hospital admission.
- Antibiotic usage (number of episodes).
- Nausea for > 72 h following IMP administration.
- Abdominal pain or discomfort for > 72 h following IMP administration.
- Diarrhoea (as defined by the World Health Organisation) [53] > 7 days following IMP administration.

#### Adverse events that do not require reporting

Death as a result of liver disease progression and other events that are primary or secondary outcome measures will not require expedited reporting. However, as such events come under the standard SAE definition, CI confirmation that the event fits one or more of these event types should be obtained.

All serious and non-serious events will be recorded on the eCRF.

#### IMP risks

FMT is a heterogeneous medicinal product, and its mechanism of action remains to be fully elucidated. As such, it is difficult to assess the risks associated with FMT administration. The primary safety concern relates to the potential transmission of pathogens from donor material.

To mitigate this risk, the existing FMT laboratory established at Guy’s and St Thomas’ NHS Foundation Trust in 2014, which holds an MHRA (MIA(IMP)) licence, will be used to manufacture FMT. The PROMISE trial adheres to stringent donor screening (for full donor enrolment and screening protocol see Supplementary Table 1) and manufacturing protocols in line with MHRA requirements and the joint British Society of Gastroenterology (BSG) and Healthcare Infection Society (HIS) guidelines for FMT use [22].

FMT is recommended by NICE (MTG71) for the treatment of recurrent *C. difficile* infection [54]. 15 patients with cirrhosis received FMT as part of the PROFIT trial (NCT02862249), and there were no IMP-related serious adverse events [26].

The majority of reported side effects from FMT are gastrointestinal and short-lived/self-limiting [19]. The side effects also relate to the route of administration, for example endoscopy. As a result of our encapsulated formulation, there is no need for endoscopic administration of FMT, therefore eliminating endoscopy-related risks.

### Trial duration

#### Expected duration of trial

Recruitment will be carried out for approximately 43 months and commenced on 21st June 2023 at King’s College Hospital NHS Foundation Trust. Follow-up will be carried out for 24 months and, during the last 6 months data analysis, reporting and dissemination will occur.

The end of trial will be defined as the database lock following completion of the last participant’s final visit. Each individual participant will remain on trial for 24 months.

#### Premature termination of the trial

The trial may be prematurely discontinued by the Sponsor, Chief Investigator or Regulatory Authority on the basis of new safety information or for other reasons given by the Data Monitoring and Ethics Committee (DMC), Trial Steering Committee (TSC), Regulatory Authority or Ethics Committee concerned. If the trial is prematurely discontinued, active participants will be informed and no further participant data will be collected. The Competent Authority and REC will be informed within 15 days of the early termination of the trial.

## Statistics

A statistical analysis plan (SAP) was drafted by the trial statistician and finalised prior to the trial statistician becoming partially blinded (able to view patients and outcome partitioned and coded as A/B). The first version of the SAP was approved on 25th May 2023. As of 20th February 2026, the SAP version is V2.0 (date 8th December 2025).

The SAP covers all of the trial analysis details (Supplementary Materials).

### Sample size

The French multicentre double-blinded RCT of the long-term fluoroquinolone antibiotic norfloxacin versus placebo in 291 norfloxacin naïve patients with advanced cirrhosis (Child–Pugh C) assessed 6-month mortality as primary outcome, as estimated by the Kaplan–Meier method. The cumulative incidence of death at 6 months was significantly lower in the norfloxacin group than in the placebo group (sub-distribution hazard ratio, 0.59; 95% confidence interval, 0.35–0.99). Norfloxacin significantly decreased the incidence of any and Gram-negative bacterial infections without increasing infections caused by *Clostridioides difficile* or multidrug-resistant bacteria. We have estimated FMT to be at least as effective as norfloxacin to reduce infection (hazard ratio = 0.625) [55].

We have assumed that 65% of participants in the control group experience an infection or decompensating event resulting in presentation to ED or hospitalisation within 2 years and that this is reduced to 48.9% in the FMT arm. In order to detect this hazard ratio of 0.625, with 80% power (alpha = 0.047) we would need to analyse 270 participants (to experience 155 events). In order to account for a 10% loss to follow up, we will inflate to 300 participants (1:1 ratio). Note: 203 events would provide 90% power to detect this effect, and 261 events would provide 80% power to detect a HR = 0.70. Using a group-sequential design, an interim analysis will be carried out after 81 events (alpha = 0.006).

### Recruitment

Trial recruitment will be monitored closely by the Trial Manager and reported at the Trial Monitoring Group (TMG) meetings. Anonymised participant pre-screening logs will be provided by sites and assessed throughout the trial. The DMC will be consulted to advise on potential strategies where there are recruitment delays such as modifications to the inclusion/exclusion criteria, targeted recruitment drives, escalation at site level to PI and/or Research and Development (R&D), opening additional study centres or site closure.

### Analysis

Primary outcome: The number of days from randomisation to first infection or acute decompensating event, whichever comes first, resulting in presentation to ED or hospital admission.

Assessment of efficacy: The main analysis will include all randomised participants in the arms to which they were randomised, regardless of what treatment they received post-randomisation, with the intent to estimate an intention-to-treat (ITT) type estimand. For the primary analysis, a treatment policy strategy will be used for any intercurrent events (such as discontinuation of treatment or death); that is, the occurrence of the intercurrent event will be considered irrelevant in defining the treatment effect of interest. Sensitivity analyses will examine this assumption using different strategies for the intercurrent events. Population definitions, target estimands and handling of intercurrent events will be further described in the statistical analysis plan.

Primary analysis: a Cox baseline-proportional hazards model of the time to infection/decompensating event will be fitted with a frailty effect to account for site heterogeneity, adjusted for baseline disease severity, participant age, sex and presence of diabetes. The adjusted hazard ratio will be presented with an associated 95% confidence interval. Proportionality will be assessed using Kaplan–Meier survival and log–log plots.

Secondary analysis: Time-to-event outcomes including mortality (all-cause; liver-related), hospitalisation, progression to ACLF, and hepatic decompensation, will be analysed in a manner consistent with the primary analysis.

Binary outcomes (infection rates, AMR incidence and antibiotic usage) will be analysed with a mixed-effects multivariable logistic regression, fitting a random effect for site, and fixed effects for patient age, sex, baseline disease severity.

Continuous outcomes (liver disease severity scores; AUDIT score; HADS; EQ-5D-5L score) will be analysed using a mixed-effects multivariable regression, adjusted in a consistent manner with the primary outcome analysis including baseline version of the outcome if applicable.

Missing data and population under investigation: Missing data will be quantified and explored for structure and breaches to the assumption of missing at random. If missing outcome data is greater than 5%, predictors of missing outcomes will be explored and if post-randomisation variables predict missingness, multiple imputation will be considered [56]. The primary population will include all randomised participants regardless of subsequent events (an “intention to treat” population). A sensitivity analysis of the primary outcome will be carried out to estimate the treatment effect for those participants who complied with the intervention (a “per-protocol” type population), by estimating the complier average causal effect [57].

Subgroup, and sensitivity analyses: The following subgroups will be used to explore for moderating effect: sex; patient age; baseline disease severity, socioeconomic status (using the index of multiple deprivation), and additional factors determined by the Trial Steering Committee.

Adherence: Patient adherence will be determined by patients taking > 70% of their allocated intervention.

### Formal interim analysis

Using an asymmetric two-sided non-binding two-stage group sequential design with 90% power and 2.5% type 1 error, we will apply a lower bound cut-off rule to conclude futility and an upper bound cut-off to determine superiority. After a minimum of 81 events, stage 1 will conclude futility if Z < 0.41 (nominal *p*-value > 0.6599), favouring FMT, or superiority if Z > 2.75 (H0: reject, nominal *p* < 0.006) favouring FMT. Applying type 1-error spending using Hwang-Shih-DeCani and gamma = − 4 (one-sided, stage one spent error = 0.003). Using this alpha spending approach, at the final analysis, the null hypothesis for efficacy will be rejected if *p* < 0.047.

### Understanding the mechanisms of action

A mediation analysis will be carried out to assess the indirect and direct effects of the mediators. Mediation analysis will be performed to investigate whether the effect of FMT on reduced infection rate is explained by a third variable/s (mediator/s) that could be also under influence of FMT. The mechanistic endpoints will be fitted as mediators (for example: markers of bacterial translocation and faecal/plasma biomarkers). Each potential mediating variable will be entered separately in mediation analyses to convey the indirect influence of FMT on the infection rate. If any of the potential mediators are not affected by the FMT, then it will not be considered as a mediator. A multi-level mediation analysis will be conducted with the estimation of various quantities for mediation including average causal mediation effect (indirect effect), average direct effect, and total effect. Subgroups will be investigated as moderating effects.

The mechanistic objectives are considered as exploratory objectives and this work will be reported outside of the primary results manuscript.

### Statistical software

Analyses will be performed using Stata (version 18 or later) statistical software package.

## Trial organisation, ethics and dissemination

### Trial Steering Committee (TSC)

This group will provide the overall supervision of the trial. It will be chaired by an independent clinician and include an independent senior statistician, an independent microbiologist, joint co-sponsor representative(s), the trial patient advocate, patient/lay representative(s) from the British Liver Trust and clinicians with experience relevant to the trial. The TSC will meet typically between six monthly and annually throughout the period of the trial, and at other times deemed necessary by the chief investigator, the sponsor or by the EME board. The TSC will be responsible for monitoring trial progress and conduct. The TSC will approve the PROMISE TSC terms of reference.

### Data Monitoring Committee (DMC)

This is an independent group that will be chaired by an independent clinician and include an independent microbiologist and an independent statistician. The DMC, in accordance with ICH GCP guidelines, is responsible for safeguarding the interests of trial participants, monitoring the main outcome measures including safety and efficacy (at interim), and monitoring the overall conduct of the trial. The DMC receives, on a periodical basis, unblinded data on trial recruitment, data quality and safety data. The DMC is required to assess emerging external evidence that might influence the ethical position of the trial. The committee is also expected to advise on any protocol modifications and whether the trial (or specific treatment groups) should be stopped early. The DMC will review recruitment and unblinded safety data per group allocated (and efficacy data at the interim analysis) and report to the TSC regarding any safety or ethical concern raised.

### Trial Management Group (TMG)

The TMG will be responsible for day-to-day trial management and will meet monthly to oversee the running of the trial. Chaired by the Chief Investigator, the TMG will be composed of PROMISE Statisticians, PROMISE Research Director, PROMISE Trial Manager, PROMISE Trial Pharmacist and several of the PROMISE co-investigators. The TMG will be responsible for reviewing and advising on trial recruitment, reviewing the final results, approving publications and approval of secondary studies.

## Ethical and regulatory approval

The trial will be conducted in compliance with the principles of the Declaration of Helsinki (1996), the principles of GCP and in accordance with all applicable regulatory requirements including but not limited to the Research Governance Framework and the Medicines for Human Use (Clinical Trial) Regulations 2004, as amended in 2006 and any subsequent amendments.

Approval has been granted by the Health Research Authority (HRA) REC [East of England Cambridge South] and the MHRA. Protocol amendments have been and will be submitted to the REC and Regulatory Authorities for approval. Approved protocol amendments will then be communicated to all co-investigators.

We will seek full REC approval for any ancillary studies and participants will undergo a separate consent process. An ancillary qualitative sub-study, entitled TRANSCEND (Faecal microbiota TRANSplantation in Cirrhotic patiENts to improve Depressive symptoms and quality of life) [IRAS project ID: REC reference: 25/WS/0036] is being conducted at King’s College Hospital. The TRANSCEND study involves a single qualitative interview during the PROMISE trial, to explore how the mental health (primary objective) and quality of life (secondary objective) of 20 participants has been affected by the PROMISE trial.

## Data management

A web-based electronic data capture (EDC) system has been designed, using the InferMed Macro 4 system. The EDC was created in collaboration with the trial analyst/s and the CI and will be maintained by the KCTU for the duration of the project. It is hosted on a dedicated server within KCL. The CI or delegate will request usernames and passwords from the KCTU. Database access will be strictly restricted through user-specific passwords to the authorised research team members. Participant initials and date of birth will be entered on the EDC. No personally identifiable information, such as NHS number, email addresses, patient names, addresses and full postcodes, will be entered into the EDC. No data will be entered onto the EDC system unless a participant has signed a consent form to participate in the trial. Each participant will be allocated a unique study number which will be used to identify samples and information collected during the trial. All participant data collected will be pseudo-anonymised and all data will be handled in accordance with the Data Protection Act 2018.

At the end of the trial, the site PI will review all the data for each participant and provide electronic sign-off to verify that all the data are complete and correct. At this point, all data can be formally locked for analysis.

At the end of the trial, all trial data will be stored in line with the Medicines for Human Use (Clinical Trials) Amended Regulations 2006 and the 2018 Data Protection Act and archived in line with the Medicines for Human Use (Clinical Trials) Amended Regulations 2006 as defined in the Co-Sponsors Archiving Standard Operating Procedure (SOP).

## Patient and public involvement

Two patient representative groups, The British Liver Trust (BLT) and King’s Liver Outpatient Advisory Group were involved in the study set-up and protocol development at all stages. A patient representative and member of the British Liver Trust senior committee were co-applicants for funding and will lead on dissemination. The study was launched at a Houses of Parliament event in March 2022.

Three focus groups, facilitated by creative artists, have been conducted with 15 patients each where the study protocol and any suggested amendments were discussed.

Results will be disseminated to study participants via Liver Research Nurses and newsletters if they indicate an interest in the study outcome.

## Data availability

Data will be openly available in a public repository at the time of trial completion and results dissemination.

## Discussion

The PROMISE study is the first Phase 3 multicentre, randomised, double-blinded, placebo-controlled trial that will evaluate the efficacy of encapsulated FMT (versus placebo) to reduce infection, decompensating events and mortality in patients with ALD, MASLD and MetALD cirrhosis.

The PROMISE study will add to the knowledge base of the use of FMT in cirrhosis, evaluating both its efficacy and underlying mechanisms of action. Infection remains a major complication in cirrhosis [3], and frequently in hepatic decompensation and worsening prognosis [3, 4, 7]. At the same time, reliance on long-term antibiotic prophylaxis has contributed to the growing burden of AMR, posing significant challenges to patient care [6, 7]. FMT offers a potential non-antibiotic strategy by restoring ecological and functional balance within the gut microbiome. Rather than simply increasing microbial diversity, FMT may re-establish key metabolic networks, improve gut barrier integrity and recalibrate immune responses [21, 26, 29], mechanisms that may collectively reduce systemic inflammation and the risk of decompensating events. FMT may also reduce invasive multidrug-resistant organism (MDRO) infection [58] and encourage MDRO decolonisation [22].

The study findings will be analysed and published in high impact open access peer-reviewed journals, disseminated at international conferences and by engagement with patient support groups, wider media and NHS organisations to ensure a broad readership. The International Committee of Medical Journal Editors (ICMJE) recommendations will be followed regarding authorship. Professional writers will not be employed. The BLT will be central in leading the dissemination of the trial results and ensuring that the public and patients will be kept fully informed. A public webinar in conjunction with the BLT will also be held as an opportunity to disseminate the study findings.

## Trial status

As of 20th February 2026, the protocol version is V4.0 date 19th June 2025. Recruitment began at King’s College Hospital on 21st June 2023. The first patient was randomised on 30th June 2023 to protocol V2.0 dated 10th March 2023. The estimated end of recruitment date is 31st December 2026.

## Supplementary Information

Supplementary Material 1. Tables 1, 2, and 3

Supplementary Material 2

## Data Availability

Data will be openly available in a public repository at the time of trial completion and results dissemination.

## Abbreviations

ACLF: Acute-on-chronic liver failure
AE: Adverse event
AMR: Antimicrobial resistance
AR: Adverse reaction
ALD: Alcohol-related liver disease
AUDIT: Alcohol-use disorders identification test
BLT: British Liver Trust
CARD: Comprehensive antibiotic resistance database
CAID: Cirrhosis-associated immune dysfunction
CI: Co-investigator
CRN: Clinical Research Network
cGMP: Current Good Manufacturing Practice
GSTT: Guy’s and St Thomas’ NHS Foundation Trust
CLD: Chronic liver disease
DMC: Data Monitoring Committee
eCRF: Electronic case report form
EDC: Electronic data capture
ELISA: Enzyme-linked immunosorbent assay
EPR: Electronic patient record
ESBL: Extended-spectrum beta-lactamase
FDA: Food and Drug Administration
FMT: Faecal microbiota transplantation
HADS: Hospital Anxiety and Depression Scale
HC: Healthy control
HKEB: Heat-killed E. coli 0111:B4
ICMJE: International Committee of Medical Journal Editors
IL: Interleukin
IL-1b: Interleukin 1 beta
IMP: Investigational medicinal product
IMPD: Investigational medicinal product dossier
IME: Important medical event
KCTU: King’s Clinical Trial Unit
KHP-CTO: King’s Health Partners Clinical Trials Office
LC-MS: Liquid chromatography tandem mass spectrometry
LPS: Lipopolysaccharide
MASLD: Metabolic dysfunction-associated steatotic liver disease
MAIT: Mucosa-associated invariant T
MELD-Na: Model for End-Stage Liver Disease sodium score
MetALD: Metabolic dysfunction and alcohol-related liver disease
MHRA: Medicines and Healthcare Products Regulatory Agency
MOF: Multi-organ failure
MRSA: Methicillin-resistant Staphylococcus aureus
MSD: Mesoscale discovery platform
NGI: National Genomics Infrastructure
NMR: Proton magnetic resonance spectroscopy
PBMC: Peripheral blood mononuclear cells
PI: Principal Investigator
PIS: Patient information sheet
PPI: Patient and public involvement
PROFIT: PROspective, randomised placebo-controlled feasibility trial of Faecal mIcrobiota Transplantation in cirrhosis
RCT: Randomised controlled trial
SAE: Serious adverse event
SAP: Statistical analysis plan
SAR: Serious adverse reaction
SOP: Standard Operating Procedure
SUSAR: Suspected unexpected serious adverse reaction
TMG: Trial Management Group
TNF-α: Tumour necrosis factor-alpha
TSC: Trial Steering Committee
UAR: Unexpected adverse reaction
VRE: Vancomycin-resistant Enterococci

## References

[1] Williams R, Aspinall R, Bellis M, et al. Addressing liver disease in the UK: a blueprint for attaining excellence in health care and reducing premature mortality from lifestyle issues of excess consumption of alcohol, obesity, and viral hepatitis. Lancet. 2014;384(9958):1953–97. 10.1016/S0140-6736(14)61838-9 25433429

[2] Devarbhavi H, Asrani SK, Arab JP, et al. Global burden of liver disease: 2023 update. J Hepatol. 2023;79(2):516–37. 10.1016/j.jhep.2023.03.017 36990226

[3] Borzio M, Salerno F, Piantoni L, et al. Bacterial infection in patients with advanced cirrhosis: a multicentre prospective study. Dig Liver Dis. 2001;33(1):41–8. 10.1016/s1590-8658(01)80134-1 11303974

[4] Jalan R, Fernandez J, Wiest R, et al. Bacterial infections in cirrhosis: a position statement based on the EASL Special Conference 2013. J Hepatol. 2013;60(6):1310–24. 10.1016/j.jhep.2014.01.024 24530646

[5] Albillos A, Lario M, Álvarez-Mon M. Cirrhosis-associated immune dysfunction: distinctive features and clinical relevance. J Hepatol. 2014;61(6):1385–96. 10.1016/j.jhep.2014.08.010 25135860

[6] Paintsil EK, Adu-Asiamah CK, Kronsten VT, et al. Global trends in antimicrobial resistance among cirrhosis patients with bacteremia: a systematic review and meta-analysis. Clin Gastroenterol Hepatol. 2025;24:S1542-3565(25)00625-1. 10.1016/j.cgh.2025.07.014 40712712

[7] Fernandez J, Acevedo J, Wiest R, et al. Bacterial and fungal infections in acute-on-chronic liver failure: prevalence, characteristics and impact on prognosis. Gut. 2018;67(10):1870–80. 10.1136/gutjnl-2017-314240 28847867

[8] Xirouchakis E, Pelekanos A, Xirouchakis S, et al. A systematic review of microbiota in cirrhosis: a change towards a more pathogenic predisposition. Int J Mol Sci. 2025;26(2):527. 10.3390/ijms26020527 PMC11765289 39859243

[9] Chang L, Liu Y, Li H, et al. Gut microbiome and its metabolites in liver cirrhosis: mechanisms and clinical implications. Front Cell Infect Microbiol. 2025;6(15):1717696. 10.3389/fcimb.2025.1717696 PMC12631333 41278477

[10] Lee S, Arefaine B, Begum N, et al. Oral-gut microbiome interactions in advanced cirrhosis: characterisation of pathogenic enterotypes and salivatypes, virulence factors and antimicrobial resistance. J Hepatol. 2025;82(4):622–33. 10.1016/j.jhep.2024.09.046 39447963

[11] Woodhouse CA, Patel VC, Singanayagam A, et al. Review article: the gut microbiome as a therapeutic target in the pathogenesis and treatment of chronic liver disease. Aliment Pharmacol Ther. 2018;47(2):192–202. 10.1111/apt.14397 29083037

[12] Riva A, Gray EH, Azarian S, et al. Faecal cytokine profiling as a marker of intestinal inflammation in acutely decompensated cirrhosis. JHEP Rep. 2020;2(6):100151. 10.1016/j.jhepr.2020.100151 PMC7391986 32838247

[13] Edwards LA, Goldenberg SD, Shawcross DL. Meeting the challenge of antimicrobial resistance in cirrhosis: the invisible threat that lies within. Gastroenterology. 2021;161(2):413–5. 10.1053/j.gastro.2021.05.043 34048780

[14] Hudson M, Radwan A, Di Maggio P, et al. The impact of rifaximin-α on the hospital resource use associated with the management of patients with hepatic encephalopathy: a retrospective observational study (IMPRESS). Frontline Gastroenterol. 2017;8(4):243–51. 10.1136/flgastro-2016-100792 PMC5641856 29067149

[15] Patel VC, Lee S, McPhail MJW, et al. Rifaximin-α reduces gut-derived inflammation and mucin degradation in cirrhosis and encephalopathy: RIFSYS randomised controlled trial. J Hepatol. 2022;76(2):332–42. 10.1016/j.jhep.2021.09.010 34571050

[16] Chang JY, Kim SE, Kim TH, et al. Emergence of rifampin-resistant staphylococci after rifaximin administration in cirrhotic patients. PLoS ONE. 2017;12(10):e0186120. 10.1371/journal.pone.0186120 PMC5628927 28982166

[17] Turner AM, Li L, Monk IR, et al. Rifaximin prophylaxis causes resistance to the last-resort antibiotic daptomycin. Nature. 2024;635(8040):969–77. 10.1038/s41586-024-08095-4 PMC11602712 39443798

[18] Boll L, Kern WV, Schuster S, et al. Frequent high-level rifaximin resistance in *Escherichia coli* associated with long-term treatment of patients with liver cirrhosis: a prospective, controlled study. Microbiol Spectr. 2025;13(11):e03347-24. 10.1128/spectrum.03347-24 PMC12584746 41081389

[19] Baxter M, Colville A. Adverse events in faecal microbiota transplant: a review of the literature. J Hosp Infect. 2016;92(2):117–27. 10.1016/j.jhin.2015.10.024 26803556

[20] Dehlholm-Lambertsen E, Hall BK, Jørgensen SMD, et al. Cost savings following faecal microbiota transplantation for recurrent *Clostridium difficile* infection. Therap Adv Gastroenterol. 2019;12:1756284819843002. 10.1177/1756284819843002 PMC6460887 31007720

[21] Allegretti JR, Mullish BH, Kelly C, et al. The evolution of the use of faecal microbiota transplantation and emerging therapeutic indications. Lancet. 2019;394(10196):420–31. 10.1016/S0140-6736(19)31266-8 31379333

[22] Mullish BH, Merrick B, Quraishi MN, et al. The use of faecal microbiota transplant as treatment for recurrent or refractory *Clostridioides difficile* infection and other potential indications: second edition of joint British Society of Gastroenterology (BSG) and Healthcare Infection Society (HIS) guidelines. Gut. 2024;73:1–24. 10.1136/gutjnl-2023-331550 38609165

[23] Bajaj JS, Kassam Z, Fagan A, et al. Fecal microbiota transplant from a rational stool donor improves hepatic encephalopathy: a randomized clinical trial. Hepatology. 2017;66(6):1727–38. 10.1002/hep.29306 PMC6102730 28586116

[24] Bajaj JS, Fagan A, Gavis EA, et al. Microbiota transplant for hepatic encephalopathy in cirrhosis: the THEMATIC trial. J Hepatol. 2025;83(1):81–91. 10.1016/j.jhep.2024.12.047 39800192

[25] Woodhouse CA, Patel VC, Goldenberg S, et al. PROFIT, a prospective, randomised placebo-controlled feasibility trial of faecal microbiota transplantation in cirrhosis: study protocol for a single-blinded trial. BMJ Open. 2019;9(2):e023518. 10.1136/bmjopen-2018-023518 PMC6398649 30772848

[26] Woodhouse C, Edwards L, Mullish BH, et al. Results of the PROFIT trial, a prospective randomised placebo-controlled feasibility trial of faecal microbiota transplantation in advanced cirrhosis. J Hepatol. 2020;73:S77–8.

[27] Kamath P, Wiesner RH, Malinchoc M, et al. A model to predict survival in patients with end-stage liver disease. Hepatology. 2001;33(2):464–70. 10.1053/jhep.2001.22172 11172350

[28] Bajaj JS, Salzman NH, Acharya C, et al. Fecal microbial transplant capsules are safe in hepatic encephalopathy: a phase 1, randomized, placebo-controlled trial. Hepatology. 2019;70(5):1690–703. 10.1002/hep.30690 PMC6819208 31038755

[29] Merrick B, Prossomariti D, Allen E, et al. Faecal microbiota transplant to ERadicate gastrointestinal carriage of Antibiotic-Resistant Organisms (FERARO): a feasibility randomised controlled trial. J Infect. 2025;91(1):106504. Epub 2025 May 16. Erratum in: J Infect. 2025 Aug 14;91(3):106583. 10.1016/j.jinf.2025.106504 40383397

[30] Conn H, Lieberthal M. The hepatic coma syndromes and lactulose. Baltimore: Williams and Wilkins; 1979.

[31] Moreau R, Jalan R, Gines P, et al. Acute-on-chronic liver failure is a distinct syndrome that develops in patients with acute decompensation of cirrhosis. Gastroenterology. 2013;144(7):1426-1437.e9. 10.1053/j.gastro.2013.02.042 23474284

[32] Cirera I, Martin Bauer T, Miguel N, et al. Bacterial translocation of enteric organisms in patients with cirrhosis. J Hepatol. 2001;34(1):32–7. 10.1016/s0168-8278(00)00013-1 11211904

[33] Dolan P. Modeling valuations for EuroQol health states. Med Care. 1997;35(11):1095–108. 10.1097/00005650-199711000-00002 9366889

[34] Bjelland I, Dahl AA, Haug TT, et al. The validity of the Hospital Anxiety and Depression Scale. An updated literature review. J Psychosom Res. 2002;52(2):69–77. 10.1016/s0022-3999(01)00296-3 11832252

[35] Saunders JB, Aasland OG, Babor TF, et al. Development of the Alcohol Use Disorders Identification Test (AUDIT): WHO collaborative project on early detection of persons with harmful alcohol consumption‐II. Addiction. 1993;88(6):791–804. 10.1111/j.1360-0443.1993.tb02093.x 8329970

[36] Trebicka J, Macnaughtan J, Schnabl B, Shawcross DL, Bajaj JS. The microbiota in cirrhosis and its role in hepatic decompensation. J Hepatol. 2021;75 Suppl 1(Suppl 1):S67-S81. 10.1016/j.jhep.2020.11.013 PMC8973011 34039493

[37] Shao L, Ling Z, Chen D, Liu Y, Yang F, Li L. Disorganized gut microbiome contributed to liver cirrhosis progression: a meta-omics-based study. Front Microbiol. 2018;18(9):3166. 10.3389/fmicb.2018.03166. PMID:30631318;PMCID:PMC6315199. PMC6315199 30631318

[38] Bajaj JS, Vargas HE, Reddy KR, Lai JC, O’Leary JG, Tandon P, et al. Association between intestinal microbiota collected at hospital admission and outcomes of patients with cirrhosis. Clin Gastroenterol Hepatol. 2019;17(4):756-765.e3. 10.1016/j.cgh.2018.07.022. Epub 2018 Jul 20 PMID: 30036646. 30036646

[39] Hamad MMAA, Connolly VM. Ambulatory emergency care - improvement by design. Clin Med (Lond). 2018;18(1):69–74. 10.7861/clinmedicine.18-1-69. PMID:29436442;PMCID:PMC6330911. PMC6330911 29436442

[40] Chan AW, Tetzlaff JM, Gøtzsche PC, et al. SPIRIT 2013 explanation and elaboration: guidance for protocols of clinical trials. BMJ. 2013;346:e7586. 10.1136/bmj.e7586 PMC3541470 23303884

[41] Kalra A, Wedd JP, Biggins SW. Changing prioritization for transplantation: MELD-Na, hepatocellular carcinoma, exceptions and more. Curr Opin Organ Transplant. 2016;21(2):120–6. 10.1097/MOT.0000000000000281 26825358

[42] Dribin TE, Schnadower D, Spergel JM, et al. Severity grading system for acute allergic reactions: a multidisciplinary Delphi study. J Allergy Clin Immunol. 2021;148(1):173–81. 10.1016/j.jaci.2021.01.003 PMC8273088 33476673

[43] Brown SG. Clinical features and severity grading of anaphylaxis. J Allergy Clin Immunol. 2004;114(2):371–6. 10.1016/j.jaci.2004.04.029 15316518

[44] Duo H, You J, Du S, et al. Liver cirrhosis in 2021: global burden of disease study. PLoS ONE. 2021;20(7):e0328493. 10.1371/journal.pone.0328493 PMC12273999 40680005

[45] Liver disease: applying all our health, Office for Health Improvement and Disparities. https://www.gov.uk/government/publications/liver-disease-applying-all-our-health/liver-disease-applying-all-our-health. Accessed Feb 2026.

[46] Shahzad M, Bokhari SSS, Rabia F, et al. Trends and demographics of liver fibrosis and cirrhosis-related mortality among adults living in the United States from 1999 to 2020: a CDC wonder analysis. JGH Open. 1999;9(10):e70247. 10.1002/jgh3.70247 PMC12474855 41019032

[47] Wang Z, Nirantharakumar K, Copland A, et al. Estimating inequality in alcohol-related liver disease burden in the UK, 2009 to 2020: a population-based study using routinely collected data. Lancet Prim Care. 2025;1(1):100002. 10.1016/j.lanprc.2025.100002 PMC12379629 40874015

[48] Office for Health Improvement and Disparities, premature mortality in 2022 in the most deprived vs least deprived deciles for England. https://www.gov.uk/government/statistics/liver-disease-profile-december-2024-update/liver-disease-profile-december-2024-update and Scottish Public Health Observatory data for Scotland https://www.scotpho.org.uk/health-conditions/chronic-liver-disease/data/mortality/. Accessed Feb 2026.

[49] Zain NMM, ter Linden D, Lilley AK, et al. Design and manufacture of a lyophilised faecal microbiota capsule formulation to GMP standards. J Control Release. 2022;350:324–31. 10.1016/j.jconrel.2022.08.012 35963468

[50] Zain NMM, Merrick B, Martin-Lilley T, et al. Bacterial diversity, viability and stability in lyophilised faecal microbiota capsules support ongoing clinical use. Int J Pharm. 2025;678:125703. 10.1016/j.ijpharm.2025.125703 40354906

[51] Singer M, Deutschman CS, Seymour CW, et al. The Third International Consensus Definitions for Sepsis and Septic Shock (Sepsis- 3). JAMA. 2016;315(8):801–10. 10.1001/jama.2016.0287 PMC4968574 26903338

[52] Trebicka J, Fernandez J, Papp M, et al. The PREDICT study uncovers three clinical courses of acutely decompensated cirrhosis that have distinct pathophysiology. J Hepatol. 2020;73(4):842–54. 10.1016/j.jhep.2020.06.013 32673741

[53] World Health Organization. Diarrhoeal disease. Fact sheet. 2024. Available from: https://www.who.int/news-room/fact-sheets/detail/diarrhoeal-disease.

[54] NICE. Faecal microbiota transplant for recurrent Clostridium difficile infection IPG485. 2014. Available from: https://www.nice.org.uk/guidance/ipg485.

[55] Moreau R, Elkrief L, Bureau C, al. Effects of long-term norfloxacin therapy in patients with advanced cirrhosis. Gastroenterology. 2018;155:1816–1827.e9. 10.1053/j.gastro.2018.08.026 30144431

[56] Jakobsen JC, Gluud C, Wetterslev J, et al. When and how should multiple imputation be used for handling missing data in randomised clinical trials – a practical guide with flowcharts. BMC Med Res Methodol 2017;17(1):1–10. 10.1186/s12874-017-0442-1 PMC5717805 29207961

[57] Peugh JL, Strotman D, McGrady M, et al. Beyond intent to treat (ITT): a complier average causal effect (CACE) estimation primer. J Sch Psychol. 2017;60:7–24. 10.1016/j.jsp.2015.12.006 28164801

[58] Ghani R, Mullish BH, Davies FJ, et al. How to adapt an intestinal microbiota transplantation programme to reduce the risk of invasive multidrug-resistant infection. Clin Microbiol Infect. 2022;28(4):502–12. 10.1016/j.cmi.2021.11.006 34826617

