# Supplementary material for: PROMISE: a PROspective randomised double-blind parallel group placebo-controlled multicentre trial of faecal MIcrobiota tranSplantation to improve outcomEs in patients with cirrhosis over 24 months—study protocol for a clinical trial
Source: Trials. 2026 Jul 31;27:632. doi: 10.1186/s13063-026-09914-x (PMC13711809; doi:10.1186/s13063-026-09914-x)
Supplement: Supplementary file 1 [file 13063_2026_9914_MOESM1_ESM.pdf]

Supplementary Materials

Table of contents

Supplementary table 1

Supplementary table 2

Supplementary table 3

FMT donor questionnaire Part 1

FMT donor questionnaire Part 2

Baseline dietary questionnaire

Patient information sheet

Informed consent form

Statistical analysis plan

Donor registered as patient at GSTT
Donors receive written and verbal information about the screening and donation processes and the use of FMT in clinical service and research studies by a registered medical practitioner
Donors provide written informed consent to undergo the screening process and donate faecal samples to be used in the study
Donors are assessed by a registered medical practitioner to determine if they meet the specified inclusion and exclusion criteria as defined by the local SOP
The screening process is documented using a donor questionnaire
Blood and stool samples will be tested according to a local SOP and in accordance with National guidelines. All diagnostic testing will be undertaken by ViaPath Analytics LLP at Guy's and St Thomas' NHS Foundation Trust. This laboratory is accredited to ISO 15189:2012 by the United Kingdom Accreditation Service (accreditation reference 8640)
If any tests are positive the donor is informed and referral to their GP is offered (with the donor's consent)
If the donor is eligible to proceed to donation, a unique donor number is assigned which is recorded in the FMT donor master file and electronic database
A record of the unique donor number is also made on the donor's electronic patient record
Results of all donor testing are recorded on their electronic patient record and are reviewed and deemed as satisfactory by a registered medical practitioner prior to release
As stool samples from donors will have metagenomic and metabolomic analyses performed (not part of standard of care), donors will also be recruited and consented as part of the PROMISE trial

Supplementary Table 1. Donor consent, screening and enrolment.

Abbreviations: FMT: faecal microbiota transplant; GP: general practitioner; GSTT: Guy's and St Thomas' NHS Foundation Trust; SOP: Standard Operating Procedure.

Full blood count (FBC)
International normalised ratio (INR)
Sodium
Potassium
Urea
Creatinine
Albumin
Aspartate transaminase (AST)
Alanine transaminase (ALT)
Alkaline phosphatase ALP)
Gamma-glutamyl transferase (GGT)
Glucose
C-reactive protein (CRP)
Bilirubin
*Glycated haemoglobin (HbA1c)
**Lipid profile (Total cholesterol, HDL cholesterol, LDL cholesterol and triglycerides)
***Urinary ethyl glucuronide/ethyl sulphate test

Supplementary Table 2. Standard of care tests. These routine tests will be performed at baseline, month-3, month-6, month-12, month-18 and month-24 by the local laboratory.

**For participants with diabetes only. **For participants with MASLD or MetALD cirrhosis only. ***If performed as standard of care.*

**Study-specific laboratory analyses**	**Baseline**	**Month 3** (+/- 14 days)	**Month 6** (+/- 14 days)	**Month 12** (+/- 14 days)
Blood (EDTA purple) 9mL x2 for bacterial DNA quantification and immunological marker assays	X	X	X	X
Blood (Clotted Gold) 5mL x1 for storage	X	X	X	X
Blood (Clotted red) 9mL x2 for metabonomic profiling (serum) and bile acid profiling (LCMS)	X	X	X	X
***London sites:**<br>Blood (Lithium heparinised green 9mL x8, for PBMC, monocyte phenotype and function assays and metabonomics (plasma)<br>**Non-London sites:**<br>Blood (Lithium heparinised green 9mL x1, for metabonomics (plasma))	X	X	X	X
Urine for metabolic profiling (20 mL sterile universal container) (metabonomic analysis with LCMS)	X	X	X	X
Faeces for biomarker, metabonomic, proteomic and cytokine analysis (20 mL sterile stool collection container)	X	X	X	X
Faeces for metagenomic sequencing (20 mL sterile stool collection container) (microbiome composition including mycome and virome diversity, pathogenic species of interest and resistome)	X	X	X	X

Supplementary Table 3. Study specific tests. Study-specific sample collection and laboratory analyses will be conducted at baseline, month-3, month-6 and month-12. Centres will be supplied with visit-specific blood, urine and faeces collection kits with detailed instructions on

collection, processing, storage and shipping. Blood will be collected in the supplied BD vacutainer tubes, centrifuged within 1-hour and stored/biobanked at -80 °C until shipping to the central laboratory at the James Black Centre, King's College London. Urine and stool samples will be collected in sterile universal containers and stored at -80 °C until shipping to the central laboratory at the James Black Centre, King's College London. At the end of the trial, blood and surplus samples collected will be stored at the King's Liver Research Tissue Bank for use in future research programmes.

* *London sites = King's College Hospital, St Mary's Hospital and St George's Hospital.*

Abbreviations: DNA: deoxyribonucleic acid; LCMS: liquid chromatography-mass spectrometry.

Guy's and St Thomas' NHS
NHS Foundation Trust

STANDARD OPERATING PROCEDURE

**TITLE: FMT Donor Consent template for clinical service**	
AUTHOR:	E Allen
For details of document's approvers, activation and review date see Q-Pulse document details.	

Faecal Microbiota Transplant (FMT) – Donor programme
Donor screening and eligibility questionnaire Part 1

Donor name	
Donor date of birth	
Donor Hospital number	
Donor number	
Donor contact details – telephone / email	
Name of assessor	
Position	
Date of assessment	

Age	Exclude if <18 or >60
Gender	☐ Male ☐ Female
Ethnicity	☐ White - White British<br>☐ White - White Irish<br>☐ White - Other<br>☐ Mixed race – White and Black Caribbean<br>☐ Mixed race – White and Black African<br>☐ Mixed race – White and Asian<br>☐ Mixed race – Other<br>☐ Asian or Asian British – Indian<br>☐ Asian or Asian British – Bangladeshi<br>☐ Asian or Asian British – Pakistani<br>☐ Asian or Asian British – Other<br>☐ Black or Black British – Caribbean<br>☐ Black or Black British – African<br>☐ Black or Black British – Other<br>☐ Chinese<br>☐ Other
Height	cm
Weight	kg
BMI	Exclude if BMI ≤18 or ≥30
Has your weight changed by more than 5lb / 2kg in the past 6 months?	☐ Yes ☐ No<br>Detail:

Guy's and St Thomas' NHS
NHS Foundation Trust

STANDARD OPERATING PROCEDURE

**TITLE: FMT Donor Consent template for clinical service**	
AUTHOR:	E Allen
For details of document's approvers, activation and review date see Q-Pulse document details.	

Describe your diet (as many as apply):	☐ Omnivore ☐ No red meat<br>☐ Vegetarian ☐ Pescatarian<br>☐ Vegan ☐ Low carbohydrate<br>☐ Kosher ☐ Lactose free<br>☐ Halal ☐ Gluten free<br>☐ Raw food only ☐ Other ______________
How many portions of fruit and vegetables do you consume per day?	☐ one or less ☐ five to six<br>☐ two to three ☐ seven or more<br>☐ three to four
How many servings of cow, sheep or goat's milk do you consume per day?	☐ one or less ☐ five to six<br>☐ two to three ☐ seven or more<br>☐ three to four
Alcohol – units/week	
Smoking/day	
Do you regularly take any illicit drugs?	☐ Yes ☐ No<br>Exclude if YES
Normal bowel habit – average Bristol Stool Consistency	☐ 1 ☐ 5<br>☐ 2 ☐ 6<br>☐ 3 ☐ 7<br>☐ 4<br>Exclude if 1, 6 or 7
Normal bowel habit – average frequency	☐ >2/day<br>☐ once to twice daily<br>☐ once / 2 days<br>☐ <once / 2 days<br>Defer if active diarrhoea (>3 UBM/day for at least 2 consecutive days)
What is your country of birth?	
Have you ever resided in another country (other than UK) for >5 years?<br>If so which countries and when?	☐ Yes ☐ No
Do you currently have a profession that is associated with an increased risk of blood-borne transmissible diseases (e.g. daily contact with patients/inmates)?<br>If yes, what profession?	☐ Yes ☐ No<br>Exclude if health worker with <u>direct</u> patient contact
Have you ever had a needle-stick or injury from a blood contaminated object from someone else?<br>If yes, when and how was this follow up?	☐ Yes ☐ No
Have you ever injected yourself or been injected with illegal or non-prescribed drugs including body building drugs or cosmetics (even if this was only once or a long time ago?)	☐ Yes ☐ No

Guy's and St Thomas' NHS
NHS Foundation Trust

STANDARD OPERATING PROCEDURE

**TITLE: FMT Donor Consent template for clinical service**	
AUTHOR:	E Allen
For details of document's approvers, activation and review date see Q-Pulse document details.	

	Exclude if YES
Have you ever had a tattoo?<br>If yes, when and in which country was it performed?	☐ Yes ☐ No<br>Defer if within past 6 months
Have you ever had a piercing?<br>If yes, when and in which country was it performed?	☐ Yes ☐ No<br>Defer if within past 6 months
Have you ever had acupuncture?<br>If yes, when and in which country was it performed?	☐ Yes ☐ No<br>Defer if within past 4 months
Have you ever undergone an operation or clinical procedure in a hospital for poor hygienic conditions?<br>If yes, when and where was this?	☐ Yes ☐ No
Have you ever had a rare infectious disease (e.g. tuberculosis, malaria, trypanosomiasis)?<br>If yes, when and which disease?	☐ Yes ☐ No<br>Relative exclusion criteria
In the last 3 months:<br>Have you had sex with anyone who has had syphilis, hepatitis or anyone who is HIV positive?	☐ Yes ☐ No<br>Defer if YES
Have you been given money or drugs for sex?	☐ Yes ☐ No<br>Defer if YES
Had sex with anyone who has ever been given money or drugs for sex?	☐ Yes ☐ No<br>Defer if YES
Had sex with anyone who has ever injected drugs?	☐ Yes ☐ No<br>Exclude if YES
Have you taken PrEP or been prescribed PEP for the prevention of HIV?	☐ Yes ☐ No<br>Defer if YES
Used drugs during sex?<br>(do not include drugs for erectile dysfunction)	☐ Yes ☐ No<br>Defer if YES
Had sex with a new partner, or had sex with more than one partner?<br>If yes, did you have anal sex with any of your sexual partners?	☐ Yes ☐ No<br>☐ Yes ☐ No<br>Defer if YES
Have you ever been treated for an intestinal infection?<br>If yes, which one and when?	☐ Yes ☐ No
Do you have any gastro-intestinal conditions:<br>Barrett's Oesophagus<br>Coeliac disease<br>Diverticular disease<br>Bariatric surgery<br>Gastric ulcer<br>Gasto-oesophageal reflux disease<br>Hepatitis<br>H. pylori infection<br>Crohn's disease	 ☐ Yes ☐ No<br>☐ Yes ☐ No<br>☐ Yes ☐ No<br>☐ Yes ☐ No<br>☐ Yes ☐ No<br>☐ Yes ☐ No<br>☐ Yes ☐ No<br>☐ Yes ☐ No<br>☐ Yes ☐ No

**TITLE: FMT Donor Consent template for clinical service**	
AUTHOR:	E Allen
For details of document's approvers, activation and review date see Q-Pulse document details.	

Ulcerative colitis<br>Other inflammatory bowel diseases<br>Irritable Bowel Syndrome<br>Lactose intolerance<br>Liver disease<br>Pancreatitis<br>Gastrointestinal malignancy or polyps	☐ Yes ☐ No<br>☐ Yes ☐ No<br>☐ Yes ☐ No<br>☐ Yes ☐ No<br>☐ Yes ☐ No<br>☐ Yes ☐ No<br>☐ Yes ☐ No<br><br>Exclude if Inflammatory Bowel Disease, Irritable Bowel Syndrome, GI malignancy, Hepatitis
Is there any family history of inflammatory Bowel Disease or colorectal cancer?	☐ Yes ☐ No<br>Relative exclusion criteria
Have you taken any antibiotics in the last 3 months?	☐ Yes ☐ No<br>Defer if YES
Have you had any of the following in the last 2 weeks?<br><br>Fever<br>Diarrhoea<br>Sore throat<br>New continuous cough<br>Swollen glands<br>Loss/change in normal sense of taste/smell	 <br>☐ Yes ☐ No<br>☐ Yes ☐ No<br>☐ Yes ☐ No<br>☐ Yes ☐ No<br>☐ Yes ☐ No<br>☐ Yes ☐ No<br>Defer for 4 weeks if YES
Have you received a live vaccination within the past 6 months?	☐ Yes ☐ No<br>Defer if YES
Have you ever been incarcerated in prison?	☐ Yes ☐ No<br>Defer if in past 4 months
Have you ever been immunosuppressed (e.g. during treatment for cancer, or for a solid organ transplant)?<br>If yes, when and why?	☐ Yes ☐ No<br>Relative exclusion criteria
Have you ever had major gastrointestinal surgery?<br>If yes, when and why?	☐ Yes ☐ No<br>Relative exclusion criteria
Have you ever suffered from metabolic syndrome or diabetes?	☐ Yes ☐ No<br>Exclude if YES
Have you ever suffered from any autoimmune condition (e.g. rheumatoid), asthma or eczema?<br>If yes, which, and do you take any treatment?	☐ Yes ☐ No<br>Relative exclusion criteria
Have you ever had any chronic pain or fatigue syndromes e.g. chronic fatigue syndrome, fibromyalgia?<br>If yes, which?	☐ Yes ☐ No<br>Exclude if YES
Have you any history of CJD or other prion disease in your family? If yes, please specify<br>Patients should be considered to be at risk from genetic forms of CJD if they have or have had<br>1. Genetic testing, which has indicated they are at significant risk of developing CJD or other prion disease	☐ Yes ☐ No

2. A blood relative known to have a genetic mutation indicative of genetic CJD or other prion disease<br>3. Two or more blood relatives affected by CJD or other prion disease	Exclude if YES
Have you ever received growth hormone or gonadotrophic treatment? If yes, please specify;<br>i) Whether the hormone was derived from human pituitary glands<br>ii) The year of the treatment<br>iii) Whether the treatment was received in the UK or in another country<br><br>Recipients of hormone derived from human pituitary glands e.g. growth hormone or gonadotrophin have been identified as potentially at risk of CJD. In the UK, the use of human growth hormone was stopped in 1985 but human-derived products may have been continued to be used in other countries. In the UK, the use of human-derived gonadotrophin was discontinued in 1973 but may have been continued in other countries after this time.	☐ Yes ☐ No<br><br>Exclude if human-derived product
Have you ever had surgery on your brain or spinal cord? People who underwent intradural neurosurgical or spinal procedures before August 1992 may have received a graft of human-derived dura mater and should be treated as at increased risk, unless evidence can be provided that human-derived dura mater was not used. Patients who received a graft of human-derived dura mater between 1980 and August 1992 are at increased risk of both sporadic CJD and vCJD.	☐ Yes ☐ No<br><br>Exclude if YES
Are you normally resident in the UK?<br>If No state country of usual residence	☐ Yes ☐ No
Which countries have you visited in the last 12 months and what was the duration of stay?<br><br>If yes state when and which countries	If travel, please refer to https://my.blood.co.uk/KnowledgeBase/Travel to determine suitability to donate.<br>Relative exclusions may apply.
In the past 12 months have you been admitted to a hospital in a country other than the UK?<br><br>If yes state when and which countries	☐ Yes ☐ No<br><br>If travel, please refer to https://my.blood.co.uk/KnowledgeBase/Travel to determine suitability to donate.<br>Relative exclusions may apply.
Are you taking any regular medications?<br><br>List all current medications:	☐ Yes ☐ No

**TITLE: FMT Donor Consent template for clinical service**	
AUTHOR:	E Allen
For details of document's approvers, activation and review date see Q-Pulse document details.	

	Exclude if any regular prescribed drugs (except OCP or dietary supplementation e.g. iron, B12, folate)

Is patient eligible to donate?	☐ Yes ☐ No
If No document reasons:	
If Yes, proceed to screening tests. Ensure consent for testing documented	
Sign and date (health care practitioner) to indicate patient has provided consent	

**Obtain serology for:**	Requested?
HIV 1 and 2 antigen/antibody	☐ Yes ☐ No
HTLV-1/HTLV-II antibodies	☐ Yes ☐ No
Hepatitis A IgG and IgM	☐ Yes ☐ No
Hepatitis B surface antigen, core antibody and DNA	☐ Yes ☐ No
Hepatitis C IgG and RNA	☐ Yes ☐ No
Hepatitis E serology and RNA	☐ Yes ☐ No
Syphilis serology (T. pallidum antibodies)	☐ Yes ☐ No
CMV IgG and IgM	☐ Yes ☐ No
EBV Serology	☐ Yes ☐ No
Strongyloides serology	☐ Yes ☐ No
**Obtain blood for:**	Requested?
Full blood count and differential	☐ Yes ☐ No
Urea, creatinine and electrolytes	☐ Yes ☐ No
Liver function tests	☐ Yes ☐ No
CRP	☐ Yes ☐ No
**Stool testing for:**	Requested?
Bacterial PCR panel (Campylobacter, Salmonella, Shigella, E. coli O:157)	☐ Yes ☐ No
Novodiag bacterial GE+ (Yersinia spp. EPEC, Vibrio spp.)	☐ Yes ☐ No
Bacterial culture (Yersinia, EPEC, Vibrio)	☐ Yes ☐ No
Clostridioides difficile	☐ Yes ☐ No
Ova, Cysts and Parasites	☐ Yes ☐ No
Norovirus	☐ Yes ☐ No
Resistant gram negative organism screen (Gent resistance/ CRE)	☐ Yes ☐ No
ESBL screen (in-house)	☐ Yes ☐ No
MRSA screen	☐ Yes ☐ No
VRE screen	☐ Yes ☐ No
Helicobacter pylori antigen	☐ Yes ☐ No
SARS-CoV-2	☐ Yes ☐ No

Screening results:

Guy's and St Thomas' NHS
NHS Foundation Trust

STANDARD OPERATING PROCEDURE

**TITLE: FMT Donor Consent template for clinical service**	
AUTHOR:	E Allen
For details of document's approvers, activation and review date see Q-Pulse document details.	

**Serology:**	Result
HIV 1 and 2 antigen/antibody	☐ Detected ☐ Not detected
HTLV-1/HTLV-II antibodies	☐ Detected ☐ Not detected
Hepatitis A IgM	☐ Detected ☐ Not detected
Hepatitis A IgG	☐ Detected ☐ Not detected
Hepatitis B surface antigen	☐ Detected ☐ Not detected
Hepatitis B core antibody	☐ Detected ☐ Not detected
Hepatitis B DNA	☐ Detected ☐ Not detected
Hepatitis C IgG	☐ Detected ☐ Not detected
Hepatitis C RNA	☐ Detected ☐ Not detected
Hepatitis E IgM	☐ Detected ☐ Not detected
Hepatitis E IgG	☐ Detected ☐ Not detected
Hepatitis E RNA	☐ Detected ☐ Not detected
Syphilis serology (T. pallidum antibodies)	☐ Detected ☐ Not detected
CMV IgM	☐ Detected ☐ Not detected
CMV IgG	☐ Detected ☐ Not detected
EBV IgM	☐ Detected ☐ Not detected
EBV IgG	☐ Detected ☐ Not detected
EBV EBNA IgG	☐ Detected ☐ Not detected
Strongyloides serology	☐ Detected ☐ Not detected
**Blood:**	Result
Full blood count and differential	☐ In range ☐ Out of range
Urea, creatinine and electrolytes	☐ In range ☐ Out of range
Liver function tests	☐ In range ☐ Out of range
CRP	☐ In range ☐ Out of range
**Stool testing (aliquoted by participant or entire donation):**	Requested?
Bacterial PCR panel (Campylobacter, Salmonella, Shigella, E. coli O:157)	☐ Detected ☐ Not detected
Novodiag bacterial GE+ (Yersinia spp. EPEC, Vibrio spp.)	☐ Detected ☐ Not detected
Bacterial culture (Yersinia, EPEC, Vibrio)	☐ Detected ☐ Not detected
Clostridioides difficile	☐ Detected ☐ Not detected
Norovirus	☐ Detected ☐ Not detected
Resistant gram negative organism screen (Gent resistance/ CRE)	☐ Detected ☐ Not detected
ESBL screen (in-house)	☐ Detected ☐ Not detected
MRSA screen	☐ Detected ☐ Not detected
VRE screen	☐ Detected ☐ Not detected
Helicobacter pylori antigen	☐ Detected ☐ Not detected
SARS-CoV-2	☐ Detected ☐ Not detected

Is all testing complete?	☐ Yes ☐ No
Comments on test results	
Accept as donor?	☐ Yes ☐ No
Any referrals required	☐ Yes ☐ No
Details of referrals made:	
Signature<br><br>Designation<br><br>Print name	Date

1) Provide donor with stool donation kit (contains collection instructions and copy of patient screening questionnaire part 2).

2) Explain requirement for stool to be fully processed within 6 hours of production.

The donor may provide multiple donations, but screening questionnaire 2 **MUST** be completed with **EACH** donation

If yes to any question, may need to undergo repeat full screening (however samples may be banked and quarantined for release after repeat testing).

If No to all questions samples may be released immediately and donor may continue to provide unlimited donations for 3 months after which time repeated screening will be required.

Guy's and St Thomas' NHS
NHS Foundation Trust

STANDARD OPERATING PROCEDURE

**TITLE: FMT Donor Consent template for clinical service**	
AUTHOR:	E Allen
For details of document's approvers, activation and review date see Q-Pulse document details.	

Faecal Microbiota Transplant (FMT) accepted donor health check

Donor Questionnaire Part 2

Donor name: ______________________________________

Date: ___________________ Time of sample: _____________________

If completing the questionnaire electronically please tick the appropriate box.

If completing the questionnaire manually please use black or blue ballpoint pen.

Since your last donation have you:

Had any diarrhoea?	NO ☐	YES ☐
Had a fever?	NO ☐	YES ☐
Had a sore throat or new continuous cough?	NO ☐	YES ☐
Had swollen glands?	NO ☐	YES ☐
Had a loss or change in normal sense of taste or smell?	NO ☐	YES ☐
Had any abdominal pain or jaundice?	NO ☐	YES ☐
Been admitted to hospital?	NO ☐	YES ☐
Felt unwell for any other reason?	NO ☐	YES ☐
Has there been any change in your general health?	NO ☐	YES ☐
Taken any new medicines or antibiotics?	NO ☐	YES ☐
Had any new sexual contacts?	NO ☐	YES ☐
Travelled abroad?	NO ☐	YES ☐

If the answer to any of these questions is YES please speak to the study doctor

---

For hospital use only:

Donor ID:

Donation ID: Accept Donation? YES / NO

Signed: ______________________________ Date:_____________________ Time: _______________

Head of FMT laboratory or designated deputy (registered medical practitioner)

The electronic template for this document is embedded with the associated document in the Q-Pulse Document Module

25 Appendix A

Dietary Questionnaire:

Describe your diet (as many as apply):	☐ Omnivore<br>☐ Vegetarian<br>☐ Vegan<br>☐ Raw food only<br>☐ Pescatarian<br>☐ No red meat<br>☐ Low carbohydrate<br>☐ Lactose free<br>☐ Gluten free<br>☐ Other
How many portions of fruit and vegetables do you consume per day?<br><br>One portion is:<br>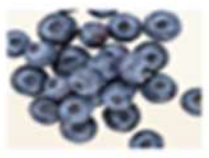<br>1 -2 handfuls of berries e.g. blueberries, strawberries<br>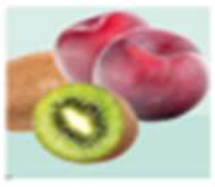<br>2 whole small fruit e.g. kiwis, plums<br>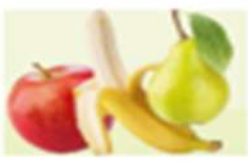<br>1 whole medium fruit e.g. apple, banana<br>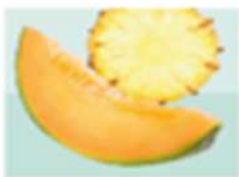<br>1-2 slices large fruit e.g. melon, pineapple<br>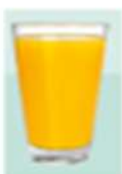<br>1 glass (150mls) fruit juice, vegetable juice, smoothie<br>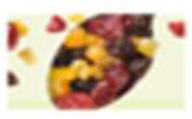<br>1 tablespoon/box (30g) dried fruit e.g. raisins<br>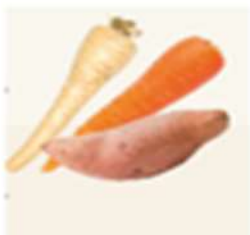<br>1 whole root vegetable e.g. carrot, parsnip<br>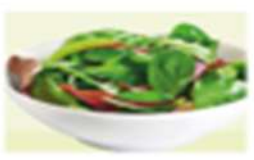<br>1 cereal bowl salad leaves e.g. spinach, lettuce<br>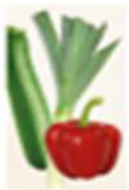<br>½-1 whole large vegetable e.g. pepper, leek<br>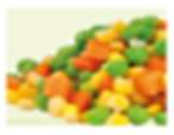<br>3-4 tablespoons of small vegetables e.g. peas, sweetcorn	☐ one or less<br>☐ two to three<br>☐ four to five<br>☐ six to seven<br>☐ more than seven
How many servings of cow, sheep or goats milk do you consume per day?<br><br>One serving is:	☐ one or less<br>☐ two to three<br>☐ four to five<br>☐ six to seven<br>☐ more than seven

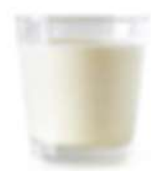 1 glass of milk (250mls)<br>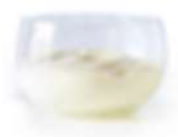 1 medium yoghurt<br>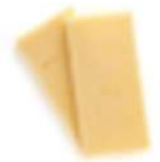 1 matchbox sized (40g) piece of cheese	
How many servings of fermented milk products (e.g. live yoghurt/cultures, kefir, cultured buttermilk, sour cream and feta cheese) do you consume per day?<br><br>One serving is:<br>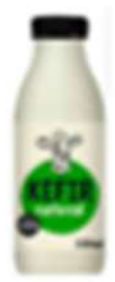 1 glass of kefir (250mls)<br>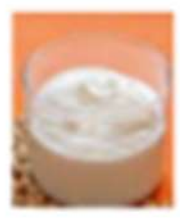 1 medium yoghurt<br>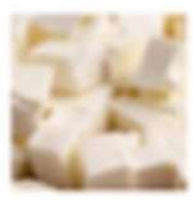 Matchbox size portion (40g) feta cheese	☐ one or less<br>☐ two to three<br>☐ four to five<br>☐ six to seven<br>☐ more than seven

PROMISE TRIAL King's College London King's College Hospital NHS Foundation Trust <<ADD TRUST LOGO HERE>>

A PROspective faecal MIcrobiota tranSplantation (FMT) trial to improve outcomEs in patients with cirrhosis (PROMISE)

Information Leaflet for Patients

We would like to invite you to take part in this research study called PROMISE

- Before you decide whether to take part, it is important for you to understand why the research is being done and what it will involve.
- Please **take time** to read the following information carefully. Discuss it with friends, family or your GP if you wish.
- You are free to decide whether or not to take part in this trial. If you choose not to take part, this will not affect the care you get from your healthcare team.
- **Ask us** if there is anything that is not clear or if you would like more information.

ABOUT THE STUDY:

1. Why are we doing this study?

Our body contains trillions of bacteria. Many of these live in our bowel which keeps us healthy and helps our immune system to fight against infections. However, an increased number of 'unfriendly' bacteria are found in the bowel of patients with liver cirrhosis (permanent liver damage), which makes them highly susceptible to infections. Antibiotics are becoming less effective as they are used so frequently, and the bowel can become infected with 'super-bugs'.

Replacing the unfriendly bowel bacteria in patients with cirrhosis with healthy bacteria donated from healthy volunteers (Faecal Microbiota Transplantation) could be highly beneficial and reduce antibiotic usage. Findings from our initial FMT trial (PROFIT Trial) showed that FMT administered during endoscopy was safe with no serious side effects. However, patients told us that they would prefer to take tablets rather than have an endoscopy.

We have therefore made oral FMT capsules from freeze-dried stool and in this study, we will test whether treating patients with these oral FMT capsules reduces the likelihood of getting an infection.

2. Why am I being asked to take part?

If you have been diagnosed with alcohol-related liver disease (ALD) or metabolic dysfunction-associated steatotic liver disease (MASLD) (previously referred to as non-alcoholic fatty liver disease) or MetALD cirrhosis (a combination of the two types of liver disease) at any time point and are aged 18 years and above, you may be eligible to take part in this study.

Unfortunately, if your cirrhosis is caused by viral hepatitis, autoimmune disease or other causes or you have previously had a liver transplant, you will be ineligible to take part in this study.

3. Who is organising and funding the study?

This study is being funded by the Efficacy and Mechanism of Evaluation Programme (EME), a National Institute for Health Research (NIHR) and Medical Research Council partnership and jointly sponsored by King's College London and Kings College Hospital NHS Foundation Trust. The doctor in charge of this study is Professor Debbie Shawcross.

4. Is the study ethical?

This study has been reviewed and given a favourable opinion by East of England Cambridge South Research Ethics Committee, who protects the rights and interests of the patients and by the Health Research Authority (HRA). The FMT to be used in this trial has been approved by the Medicines and Healthcare products Regulatory Agency (MHRA).

5. How long is the study expected to run for?

The trial will run for 5-years. Recruitment for this study will be carried out over 18 -months followed by a follow-up period of 2 years. Each patient will remain on this trial for 24 months from the start of treatment. We aim to recruit 300 patients from 16 hospitals around the United Kingdom.

ABOUT TAKING PART:

6. What will I need to do if I take part?

If you decide to take part, you will be given this information sheet to keep and asked to sign an informed consent form confirming your understanding of the study and agreement to take part. You may contact the research team **(details on the last page of this information sheet)** if you have any questions about the study, and these will be fully answered. You will be given a copy of the consent form for your records.

7. What will happen to me if I agree to take part?

If you agree to take part in the study, you will be assessed by an authorised investigator at our outpatient clinic for your suitability to be involved in this study. Your suitability will be assessed as per the below inclusion criteria:

i) You should be 18 years and above.
ii) You have a confirmed diagnosis of alcohol-related liver disease (ALD) or metabolic dysfunction-associated steatotic liver disease (MASLD) or MetALD cirrhosis.
iii) If you have liver disease cirrhosis, your alcohol consumption on average should be no more than 20 grams/day [1 unit of alcohol contains 10mLs or 8g of alcohol].
iv) You have not received antibiotics or probiotics within 7 days prior to participation in the trial.
v) You have the capacity to provide written informed consent to participate.
vi) You have no history of a liver transplant.
vii) You do not have a moderate, severe or life-threatening food allergy (e.g., peanut allergy) that has ever required treatment or hospitalisation.
viii) You have not received another investigational drug or device 4 months prior to participation in the trial.
ix) You are not pregnant or breastfeeding. Urine testing will be performed to rule out pregnancy in females.
x) You have not been treated for acute variceal bleeding, infection, hepatic encephalopathy, or bacterial peritonitis within 14 days prior to participation in the trial.

If you are happy to proceed you will be asked to sign a consent form by a member of our research team. You will then be randomly allocated (like tossing a coin) by a web-based system to one of two treatment groups:

1) The 'active treatment' group: Participants in this group will receive the oral treatment being tested (FMT capsule), or
2) The comparison or 'control group': Participants in this group will receive an oral 'placebo' treatment. The placebo will be a dummy capsule and will NOT contain any stool. This method of using a 'placebo control' is used to make sure the study results are reliable by checking that any changes measured are actually because of the active drug and not just because of chance.

Randomisation ensures that participants in both groups are as similar as possible, so you will have a 50% chance of getting either of the treatments. This is the only way we can compare the groups and make sure the study is fair.

This study will be double blinded; meaning neither the researcher nor the participant will know which treatment they are getting.

You will be followed up at set time points during the trial to see how effective the active treatment is based on various measurements taken.

Your GP will be kept informed of your participation in the trial. By consenting to take part, you will agree to us sharing your progress in the trial with your GP which forms a part of your Standard of Care.

8. Treatment visits and summary

*Each clinic visit will last for ***approximately 2 HOURS,*** and all research visits will be prioritised meaning you may not need to wait for a long time on the day of your appointment* **<<Site to Amend/Remove as Appropriate>>**

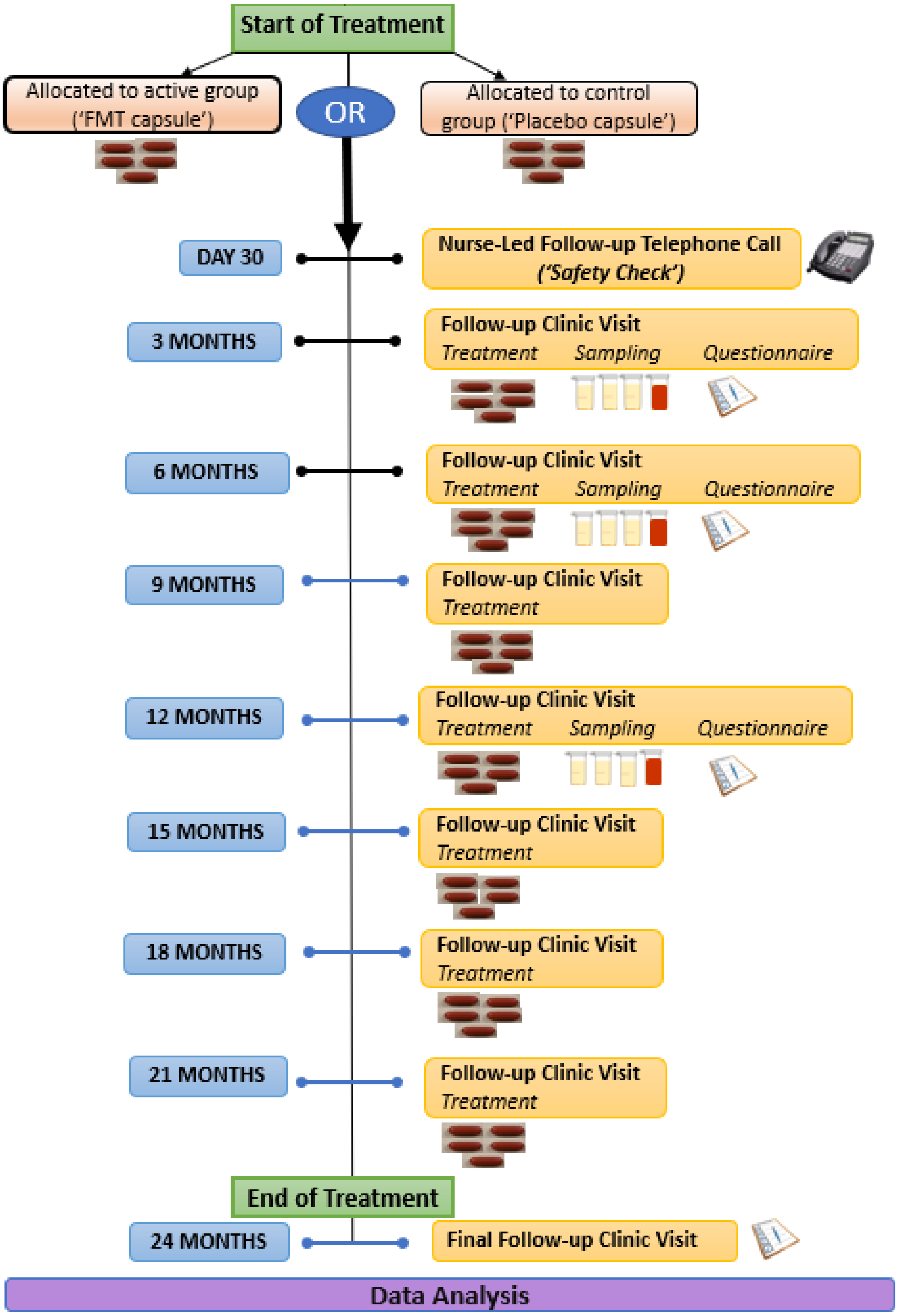
Start of Treatment
Allocated to active group ('FMT capsule')
OR
Allocated to control group ('Placebo capsule')
DAY 30
Nurse-Led Follow-up Telephone Call ('Safety Check')
3 MONTHS
Follow-up Clinic Visit
Treatment Sampling Questionnaire
6 MONTHS
Follow-up Clinic Visit
Treatment Sampling Questionnaire
9 MONTHS
Follow-up Clinic Visit
Treatment
12 MONTHS
Follow-up Clinic Visit
Treatment Sampling Questionnaire
15 MONTHS
Follow-up Clinic Visit
Treatment
18 MONTHS
Follow-up Clinic Visit
Treatment
21 MONTHS
Follow-up Clinic Visit
Treatment
End of Treatment
24 MONTHS
Final Follow-up Clinic Visit
Data Analysis

Before the treatment (First Research Visit):

At your first clinic visit and before starting treatment with either the FMT capsule or the placebo capsule, you will undergo baseline screening and assessment which will involve the following:

- Physical examination: Height, Weight, Blood Pressure, Temperature, Heart Rate and Oxygen Saturation
- Current medication usage
- Routine blood samples: These tests are part of your standard medical care.
- A urine pregnancy test will be performed for all females of childbearing potential.

(Note: The pregnancy urine tests will be disposed of in the clinic room in clinical waste bins/bags in accordance with our hospital's clinical waste procedures.)

- Additional Sampling*:
- Blood sampling* – For patients with diabetes and metabolic dysfunction-associated steatotic liver disease (MASLD), you will be asked to provide additional blood samples to measure your HbA1C levels and lipid profile which are usually taken anyway as part of standard medical care.
- Urine sampling* – you will be asked to provide a urine sample (approx. 20 mL). You may collect your urine sample at home and bring it to your appointment if this is easier for you.
- Stool sampling* – you will be asked to provide a stool sample to measure what types of bacteria are found, and in what patterns and quantities. You may collect your stool sample at home and bring it to your appointment if this is easier for you.
- Questionnaires*:
- You will be asked to complete a short dietary questionnaire, a quality-of-life questionnaire, and a questionnaire to assess your current mental health. Each questionnaire should take no longer than 5 minutes to complete.
- You will be asked whether you have been abroad recently. This is to measure any significant daily dietary changes.
- For patients with alcohol-related cirrhosis – you will be asked to complete an additional questionnaire to assess your alcohol consumption which should take no longer than 5 minutes to complete.

Start of the Treatment (DAY 0):

Followed by the completion of the assessment at the clinic, you will be randomly allocated to receive one of the two treatments, where you will receive 5 capsules of the medicine (either the FMT or the placebo) to be taken by mouth and swallowed with water or cordial. You will be recommended to fast for at least 4 hours prior to your treatment, which you may choose to do prior to your hospital visit. This is to help with treatment absorption and to prevent vomiting after swallowing the capsule. Light snack is allowed if medically advised by your study doctor.

Telephone Follow-Up (DAY 30):

On day 30, you will receive a telephone call from your research nurse to check for adverse events (if any) and to record any changes to the medications you are taking.

Follow-Up Clinic Visits:

After the start of the treatment, you will be followed up at different time points during the trial to see how effective the active treatment is based on various measurements taken. You will be recommended to fast for at least 4 hours prior to your treatment, which you may choose to do prior

to your hospital visit. This is to help with treatment absorption and to prevent vomiting after swallowing the capsule. Light snack is allowed if medically advised by your study doctor.

At each follow-up clinic visit – 3, 6, 9, 12, 15, 18 and 21 months, you will receive 5 capsules of the medicine (either the FMT or the placebo) to be taken by mouth and swallowed with water or cordial.

- Routine (standard of care) blood samples will be taken at 3, 6, 12, 18 and 24 months.
- Additional Sampling* (Blood, Stool and Urine) - At 3, 6 and 12 months, additional samples of blood, stool and urine will also be collected.
- Questionnaires* - At 3, 6, 12, 18 and 24 months, you will be asked to repeat the quality-of-life questionnaire, and a questionnaire to assess your current mental health status. For patients with alcohol-related cirrhosis – you will be asked to complete an additional questionnaire to assess your alcohol consumption. Each questionnaire should take no longer than 5 minutes to complete.
- You will be asked whether you have been abroad recently. This is to measure any significant daily dietary changes.
- Travel expenses will be reimbursed for up to 4 additional follow-up/treatment visits at max £30 return per visit. For trial centres located in rural areas, if you require additional support with travel expenses, please contact your local trial team.

9. Do I have to take part, and what if I change my mind?

Participation is completely voluntary. It is up to you whether you wish to take part or not. If you decide to take part, you are still free to withdraw at any time and without giving a reason. This will not affect the medical care you will receive.

Your rights to access, change or move your information are limited, as we need to manage your information in specific ways in order for the research to be reliable and accurate. If you withdraw from the project, we will keep the information about you that we have already obtained. To safeguard your rights, we will use the minimum personally identifiable information possible.

For us to assess the potential benefits and effectiveness of this innovative treatment, we would like to encourage you to attend all the follow-up visits. If you decide that you no longer want to continue with the treatment, you have the option of not attending hospital visits. If possible, we would like to continue gathering information during the remainder of your follow-up period through telephone calls and access to your medical records. For further information, please discuss with your study team.

10. What are the possible benefits of taking part?

You will be taking part in a new and innovative trial to assess the beneficial effects of FMT capsules in patients with cirrhosis. We hope that the knowledge we will gain from this study will improve our understanding of the way in which FMT works, and the role of the gut and immune system in liver cirrhosis.

We will also gather information during the trial on how the FMT works in patients with cirrhosis including the impact on the make-up and function of the gut microbiome. We

will examine whether FMT can reduce the likelihood of developing an infection and being admitted to the hospital.

11. What are the possible disadvantages and risks of taking part?

There is a theoretical risk associated with the transmission of infectious agents from the donor, but FMT has been shown to be very safe in several thousand patients who have received it. Any potential risk of infection is mitigated by rigorous selection and screening of the stool donors. This is in accordance with National guidance issued by the British Society of Gastroenterology and the Healthcare Infection Society, and the European consensus statement issued by United European Gastroenterology. The donor stool is also stored for 30 years for full traceability should in the future there be any concerns regarding the potential transmission of infection.

There are no disadvantages or risks involved in taking part with regard to the samples being obtained, and the amount of blood taken will not be harmful to you. The placebo treatment is not harmful but is not expected to have any benefit on your clinical condition as it contains no active drug or treatment. All patients, regardless of whether they receive placebo or FMT have the same rigorous follow up and support from the trials team. You will be closely monitored after the treatment is given and will be seen at regular intervals afterwards to see if you have experienced any side effects.

We do not anticipate any serious problems to occur after the FMT or placebo treatment, but you will be given contact numbers should you run into any problems outside of the trial visits.

12. Will my details be kept confidential?

Your data will be processed under the terms of UK data protection law (including the UK General Data Protection Regulation (UK GDPR) and the Data Protection Act 2018).

- King's College London and King's College Hospital NHS Foundation Trust are the joint sponsors for this trial and will act as a data processor and data controller for the trial.
- You will be given a unique study number, which will be used to identify any samples and information collected during the research.
- All participant data collected will be pseudo-anonymised and will be stored on a password-protected database, or in a file that will be stored in locked cabinets with restricted office access at research sites.
- Research data collected will be inserted into an electronic database, InferMed Macro 4 system which is maintained on a secure server by the King's Clinical Trials Unit (KCTU). Database access will be strictly restricted through user-specific passwords to the authorised research team members. Personally identifiable

information (such as name, email address, postal address, postcode and NHS Number) will not be recorded on the database.

- During monitoring visits, we will need to use information from you and your medical records for this research project. This information includes your
    - Initials,
    - NHS number,
    - Name,
    - Contact details

  People will use this information to do the research or to check your records to ensure that the research is being done properly.
- The people who analyse the information will not be able to identify you and will not be able to find out your name, NHS number or contact details.
- In case of an emergency and for your safety, investigators may have to disclose information about you and the treatment you are receiving for the appropriate medical management to be provided.
- We advise that you inform your private medical insurance provider (if you hold private medical insurance) of your participation in this study - this again is a routine procedure for this type of research.
- The sponsor will keep the research data collected from you for 15 years after the trial has finished.

We will keep all information about you safe and secure.

13. What will happen to any samples that I give?

Every effort will be made to ensure blood samples for the study are taken at the same time as samples taken as part of your routine clinical care in the outpatient clinic – so that there will be no additional discomfort to you as a result of being involved in this study. The amount of blood taken at each study visit is approximately 86 mLs or 17 teaspoonfuls. This is a very small amount of blood (your body contains 5000 mLs of blood) and will not adversely affect you in any way.

Samples of collected blood, urine and stool will be processed and stored in a locked freezer for future analysis at King's College London. Some analyses might be undertaken at a genomic facility in the UK or Sweden. Analyses will include an assessment of the function of the immune system and isolation of bacterial DNA to determine the composition and function of the gut microbiome in response to the FMT treatment.

Advances in science and technology happen all the time and it may be possible that over the course of the trial or after the trial has ended that new techniques become available that can give us a better understanding of your disease and the treatment we are testing. We would like to be able to keep your samples in the King's Liver Research Tissue Bank so that we may be able to further relevant research on your samples with the aim of progressing research into liver cirrhosis and the gut microbiome in the future.

We would therefore like to transfer any excess samples remaining to the King's Liver Research Tissue Bank where we store blood and surplus samples for use in research programmes. We would like you to consider whether you are happy to participate in this and donate to the Tissue Bank. All samples stored and tested will be fully anonymised.

14. Where can I find out more about how your information is used?

You can find out more about how we use your information

- at www.hra.nhs.uk/information-about-patients/
- our leaflet available from https://www.kcl.ac.uk/research/support/research-ethics/kings-college-london-statement-on-use-of-personal-data-in-research
- by asking one of the research team
- by contacting King's College London's Data Protection Officer, Mr Albert Chan at info-compliance@kcl.ac.uk

If you are not happy with their response or believe they are processing your data in a way that is not right or lawful, you can complain to the Information Commissioner's Office (ICO) (www.ico.org.uk or 0303 123 1113).

15. What if something goes wrong?

If you have a concern about any aspect of this study, you should ask to speak to your study team or the trial manager who will do their best to answer your questions. If you remain unhappy and wish to complain formally, you can do this through the NHS Complaints procedure by contacting your local Patient Advice Liaison Service (PALS) office <<Site to Add>>. Details of your local office can be obtained by asking your study doctor, GP, telephoning your local hospital or looking on the NHS choices website. In Scotland, you can contact your local hospital's Patient Experience Team <<Site to Add>>.

Every care will be taken in the course of this study. However, in the unlikely event that you are injured by taking part, compensation may be available.

In the event that something does go wrong, and you are harmed during the research, and this is due to someone's negligence then you may have grounds for a legal action and compensation against the sponsors King's College Hospital NHS Foundation Trust and King's College London, but you may have to pay your legal costs. King's College London maintains adequate insurance to cover any liabilities arising from the study

16. What will happen to the results of the clinical trial?

When we have results for an adequate number of patients after the study is completed, we plan to publish them in high impact medical journals and national and international conference presentations, involving patient support groups led by the British Liver Trust, wider media, and NHS organisations. We will ensure the research impacts on the

management of patients with cirrhosis shaping policy and guideline development. We can provide you with a summary of the results of the study when available should you desire this.

17. Study contact details:

If you have any questions regarding the study, please contact the study team at the numbers below:

For Information on Treatment and Appointments:	
**Principal Investigator at <<SITE NAME>>:**<br><<ADD Name>>	Email:<br>Tel:
**Site Research Nurse:**<br><<ADD NAME>>	Email:<br>Tel:

Thank you for taking the time to read this information leaflet and for considering taking part in this clinical trial.

Study Title: A **PRO**spective randomised double-blind parallel-group placebo-controlled multicentre trial of faecal **MI**crobiota tran**S**plantation (FMT) trial to improve the primary outcom**E** (first hospitalisation due to infection) in patients with cirrhosis over 24 months **(PROMISE)**

REC Number: 22/EE/0247

Study Number: NIHREME130730

Participant Identification Number: ☐

INFORMED CONSENT FORM - PATIENT

Please add your initials in the boxes:

1. I confirm that I have read and understand the Participant Information Sheet dated ________________ (version ____________) for the above study. I have had the opportunity to consider the information, ask questions and have had these answered satisfactorily. I have been given a copy of the Participant Information Sheet to keep. ☐

2. I understand that my participation is voluntary and that I am free to withdraw at any time, without giving any reason, without my medical care or legal rights being affected. ☐

3. I understand that relevant sections of my medical records and data collected during the study may be looked at by responsible individuals or regulatory authorities or from the NHS Trust, where it is relevant to my taking part in this research. I give permission for these individuals to have access to my records. I understand that my personal data will be processed and stored securely in compliance with the Data Protection Act 2018 and the General Data Protection Regulation. ☐

4. I consent to the following samples being collected, stored, and analysed: Blood, stool and urine ☐

5. I agree that any surplus samples (including DNA) will be transferred into and stored in the Liver Biobank, and may be used in future, anonymised research. ☐

6. I understand that the information and samples collected from me will be used to support other research in the future, and maybe shared anonymously with other researchers at different organisations which could be at a genomic facility in the UK or Sweden. ☐

7. I understand that my General Practitioner will be informed of my participation in the study. ☐

8. I agree to take part in the above study. ☐

Optional Consent:

9. I agree to my contact details being stored to enable me to be contacted about any future research studies. ☐

10. I would like to be sent information regarding the results of this study when available. ☐

Name of Participant	Signature	Date
Chief/Principal/Sub-Investigator	Signature	Date

Records:

1) Investigator Site File *(original)*
2) Participant *(copy)*
3) Hospital notes *(copy)*

King's College Hospital NHS
NHS Foundation Trust

PROMISE Trial: A PROspective randomised double-blind parallel group, placebo-controlled multicentre trial of faecal MIcrobiota tranSplantation to improve the primary outcomEs (first hospitalisation due to infection) in patients with cirrhosis by 24 months.

EudraCT Number: 2022-000300-35

Statistical Analysis Plan
Version 2.0
08/12/2025

Authors:
Dominic Stringer, Francesca Maher, Prof Ben Carter

Trial Statistician: Francesca Maher
Signature.. Date......................................
Francesca Maher (27/01/2026 11:07:55 GMT) 27/01/2026

Senior Statistician: Professor Ben Carter

Signature.. Date......................................
Ben Carter
Ben Carter (27/01/2026 11:10:55 GMT) 27/01/2026

Chief Investigator: Professor Debbie Shawcross

Signature.. Date......................................
Debbie Shawcross (27/01/2026 11:22:02 GMT) 27/01/2026

Trial Steering Committee Chair: Professor William Alazawi

Signature.. Date......................................
William Alazawi
William Alazawi (17/02/2026 10:48:48 GMT) 17/02/2026

Purpose and Scope of Statistical Analysis Strategy

This document details the presentation and analysis strategy for the primary papers reporting results from the PROMISE trial. It is intended that the results reported in these papers will follow the strategy set out herein; subsequent papers of a more exploratory nature will not be bound by this strategy but will be expected to follow the broad principles laid down for the principal paper(s). These principles are not intended to curtail exploratory analysis or to prohibit sensible statistical and reporting practices, but they are intended to establish the strategy that will be followed as closely as possible, when analysing and reporting the trial. Reference was made to the trial protocol v3.0 03.11.2023, ICH (13) guidelines on Statistical Principles (E9) and CONSORT guidelines (14).

This trial is being conducted by KCTU and this Statistical Analysis Plan (SAP) has been developed following KCTU Statistics Standard Operating Procedure ST-02.

1 DESCRIPTION OF THE TRIAL 6

1. Key Trial Personnel 6
2. Principal research objectives to be addressed. 6
3. Endpoints 7
4. Trial designs 9
5. Method of allocation of groups 9
6. Duration of the treatment period 10
7. Frequency and duration of follow-up 10
8. Visit windows 10
9. Data collection 10
10. Inclusion/Exclusion criteria 10
11. Measures 11

1.11.1 Measures collected at Baseline only 11
1.11.2 Primary outcome 11
1.11.3 Secondary outcomes 12

Sample size estimation (including clinical significance) 14

2 DATA ANALYSIS PLAN – DESCRIPTIVES 14

1. Recruitment and representativeness of recruited participants 14
2. Baseline comparability of randomised groups 16
3. Anticipated intercurrent events 16
4. Adherence to allocated treatment and treatment fidelity 16
5. Loss to follow-up and other missing data 17
6. Adverse event reporting 17
7. Assessment of outcome measures (planned unblinding of trial statistician) 17
8. Blinding status of personnel and planned unblinding 17
9. Brief description of proposed analyses and any pre-analysis statistical checks required 18
10. Descriptive statistics for outcome measures 18
11. Listing of concomitant medications 18

3 DATA ANALYSIS PLAN – INTERIM ANALYSES 18

4 DATA ANALYSIS PLAN – INFERENTIAL ANALYSES 19

1. Primary analysis description 19

4.1.1 Definition of primary estimand 19

4.1.2 Further details on handling of intercurrent events and subsequent supplementary analyses 19

2. Main analysis of treatment differences (intention-to-treat population set) 20

3. Per protocol population set 20

4.3.1 Definition of protocol deviation/violation 21

4. Analysis of primary outcomes 21

4.4.1 Primary Efficacy Parameters 21

5. Analysis of secondary outcomes 21

4.5.1 CONTINUOUS OUTCOMES 22

4.5.2 BINARY OUTCOMES 22

4.5.3 TIME TO EVENT OUTCOMES 22

6. Safety 22

7. Time points 22

8. Stratification and clustering 22

9. Missing items in scales and subscales 23

10. Missing baseline data 23

11. Missing outcome data 23

12. Method for handling multiple comparisons 23

13. Method for handling non-compliance 23

14. Model assumption checks 24

15. Sensitivity analyses 24

16. Planned subgroup analyses 25

5 Quality Control and Quality Assurance 25

1. Quality control 25

2. Internal Pilot 'Go/No Go' progression 25

3. Carrying out interim analysis and final analysis 26

4. Approval and version control of the SAP 26

5. Quality Assurance 26

6 SOFTWARE 27

7 SCHEDULE OF ASSESSMENTS AND MEASURES 28

8 SCORING 32

9 DUMMY TABLES FOR FINAL ANALYSIS 36

Descriptive baseline characteristics 36

Primary outcome 38

Intercurrent events 39

Secondary outcomes 40

Compliance 40

Safety/Adverse events Reporting 41

Unblinding events 44

10 REFERENCE LIST 45

11 AMENDMENTS 46

1 DESCRIPTION OF THE TRIAL

1. Key Trial Personnel

Chief investigator:
Prof Debbie Shawcross

King's Clinical Trials Unit (KCTU), Trial statisticians:
Trial Statistician: Francesca Maher
Senior Statistician: Prof Ben Carter

UK funding is from the NIHR EME Programme (NIHREME130730)

2. Principal research objectives to be addressed

The **clinical efficacy aim** of the PROMISE Trial will be to evaluate the efficacy and mechanisms of action of encapsulated Faecal Microbiota Transplantation [FMT] (versus placebo) to reduce infection and mortality in participant's with alcohol-related liver disease (ALD), metabolic dysfunction-associated steatotic liver disease (MASLD), and metabolic dysfunction-associated steatotic liver disease and alcohol-related liver disease overlap (MASLD-ALD Overlap).

The **clinical efficacy objectives are** as follows:

- (Primary objective) Evaluate the efficacy of encapsulated FMT to reduce the susceptibility of infection in participants with cirrhosis measured by the time to first infection or decompensating episodes of cirrhosis resulting in presentation to the emergency department (ED) or hospitalisation.
- Evaluate the efficacy of encapsulated FMT to reduce non-acute decompensating cirrhotic events (not requiring presentation to hospital/hospitalisation) including new-onset or worsening ascites and covert (Westhaven criteria grade 0-1) hepatic encephalopathy.
- To evaluate the impact of encapsulated FMT on participant's outcomes including all-cause hospitalisation, disease progression (including development of acute-on-chronic liver failure), quality of life, mental health, alcohol use and survival.
- To assess the impact of FMT on antibiotic usage and loss/reduction of antimicrobial resistance (AMR) genes.
- To assess the safety of encapsulated FMT.

Following the assessment of efficacy, our **mechanistic aims are**:

- To determine the mechanisms of action on the basis of the hypothesis that the characteristics of the gut microbiome in cirrhosis drive the development of cirrhosis-associated immune dysfunction [CAID].

- To determine if FMT will favourably modify key contributors by improving gut microbiome diversity and reducing bacterial translocation into the systemic circulation.

The **mechanistic objectives** are:

- Determine whether FMT increases the diversity of the gut microbiome in cirrhosis.
- Determine whether FMT reduces bacterial translocation and endotoxemia.
- Evaluate whether FMT improves CAID and reduces systemic inflammation.
- To evaluate whether FMT reduces portal hypertension as a driver of bacterial translocation.

3. Endpoints

Primary endpoint:

1. **Time to first infection resulting in presentation to the Emergency Department (ED) or admission into hospital with a confirmed infection as defined in the criteria below:**
2. **Decompensating episodes as defined in the criteria below:**
    a. **Resulting in presentation to the Emergency Department**
    b. **Resulting in hospital admission**

Definition of infection

For the purposes of this study, infection is defined as outlined below:

Lower respiratory tract infection	New pulmonary infiltrate on chest x-ray (CXR) in the presence of at least one respiratory symptom (cough, sputum production, dyspnoea, pleuritic pain) or one sign of infection (core body temperature >38°C or less than 36°C, shivering, or leukocyte count >10,000/mm3 or <4,000/mm3) in the absence of antibiotics. This includes the presence of **respiratory tract viral infections**, including COVID-19, contracted in the community resulting in a hospital admission with a positive test on viral PCR. Viral infections contracted whilst in hospital will not qualify as meeting the primary endpoint.
Urine infection	Urine white blood cell >15/high-power field (or equivalent) with either positive urine Gram stain (or cytometry) or culture.
Catheter associated infection	Evidence of an organism grown on urine culture or positive urine gram stain (or cytometry), plus symptoms to suggest systemic infection such as suprapubic pain, pyuria, fever >38°C, signs of sepsis.

Spontaneous bacterial peritonitis	Ascitic white cell count >250 cells/mm3 and/or positive ascitic culture.
Gastrointestinal tract infection	Positive stool culture/PCR for a pathogen typically associated with gastrointestinal infection, including the presence of *Clostridioides difficile* if suspected.
Cellulitis or Soft Tissue Infection	Localised area of pain tenderness, swelling, erythema and heat, with a positive swab/tissue sample on culture or requiring intravenous antibiotics with evidence of improvement after treatment.
Bone Infections/Osteomyelitis	Positive microbial culture and/or PCR of a bacterial organism from biopsy of bone.
Bacteraemia	The presence of bacteraemia with a positive line tip or peripheral blood cultures.
Central nervous system (meningitis or encephalitis	Positive cerebrospinal fluid microscopy, culture and/or PCR with a pathogen typically associated with meningitis/encephalitis. This includes viral, bacterial and fungal infection.
Joint Infections	Localised area of pain, tenderness, swelling, erythema and heat, with joint fluid analysis compatible with infection and positive microbial culture and/or PCR of a bacterial organism.
Intra-abdominal Infections	Symptoms of abdominal pain, tenderness or jaundice (to suggest cholangitis) combined with core body temperature >38°C or less than 36°C, rigors or leukocyte count >10,000/mm3 and confirmatory findings on imaging to suggest an infective source such as cholecystitis or intra-abdominal abscess.

Definition of decompensating episodes which result in presentation to ED (including day case paracentesis unit) or hospitalisation:

- New onset moderate or large volume ascites requiring diuretic therapy and/or paracentesis (this can include patients who are referred to an outpatient paracentesis service)
- Variceal bleeding confirmed following emergency endoscopy or on CT angiography suggestive of bleeding elsewhere in the gastrointestinal tract as a consequence of portal hypertension.
- Overt hepatic encephalopathy (Westhaven criteria grade 2-4)

Secondary endpoints:

- Time to first infection resulting in hospitalisation over 24 month follow up period (former primary endpoint)

- Incidence of decompensating events including hepatic encephalopathy, new-onset or worsening ascites, variceal bleeding and spontaneous bacterial peritonitis over 24 month follow up period.
- Incidence of all-cause infection over 24 month follow up period, including infections not resulting in hospitalisation.
- Progression to ACLF (the development of one or more organ failures).
- Infection rates and antibiotic usage following randomisation over 24 month follow up period.
- Incidence of AMR including skin and nose colonisation with methicillin-resistant Staphylococcus aureus (MRSA), vancomycin-resistant Enterococci (VRE), linezolid-resistant Enterococci (LRE), extended-spectrum beta-lactamase producing bacteria (ESBL), fluoroquinolone-resistant gram negative and carbapenem-resistant Enterobacteriales (over 24 month follow up period).
- Hospitalisation rates (liver-related and all-cause) including the length of stay and admission to high dependency/intensive care (over 24 month follow up period).
- Change in liver disease severity scores at 3-, 6-, 12-, 18- and 24-months post-randomisation
- Change in quality of life (EQ-5D-5L score) at 3-, 6-, 12- and 24-months post-randomisation.
- All-cause mortality, and liver-related mortality.
- Change in depression and anxiety score using HADS at 3-, 6-, 12- and 24-months post-randomisation.
- Change in alcohol use disorder-related events in participants enrolled with alcohol-related cirrhosis at 3-, 6-, 12- and 24-months post-randomisation, as assessed by the alcohol-use disorders identification test (AUDIT score) and urinary ethyl glucuronide/ethyl sulphate levels if tested as part of the standard of care.
- Safety based on assessments including vital signs, physical examination, clinical laboratory evaluation, and reported as adverse events (AEs) or serious adverse events (SAEs) over 24-month follow-up period.

4. Trial designs

PROMISE is a Phase 3 multicentre, randomised, double-blinded, parallel group placebo-controlled trial evaluating encapsulated FMT versus matched placebo for 2 years. Participants will be randomised (1:1) to FMT or placebo. Participants will receive the investigational medicinal product (IMP) every 3 months +/- 14 days for 21 months or until reaching the primary endpoint and will be evaluated at baseline before the IMP is administered and at 30-days (nurse-led telephone interview), 3-months, 6-months, 1 and 2 years. Blood, stool, and urine will be collected at baseline, 3-months, 6-months and 1 year.

5. Method of allocation of groups

Participants will be randomised on a 1:1 basis to either FMT or placebo. Randomisation will be at the level of the individual using the method of block randomisation with varying block sizes, stratified by site.

A web-based randomisation system will be designed, using the bespoke King's Clinical Trials Unit (KCTU) randomisation system. The randomisation system will be created in collaboration with the trial analyst/s and the CI and maintained by the KCTU for the duration of the project. It will be hosted on a dedicated server within KCL.

6. Duration of the treatment period

Participants will receive five capsules of the Investigational Medicinal Product (IMP) to be taken by mouth and swallowed every 3 months +/- 14 days for a total of 21 months. Participants must fast for 4-hours prior to treatment. They will be requested to ingest all capsules within 30 minutes and are permitted unlimited water or squash. The last treatment will be administered at the 21-month visit.

7. Frequency and duration of follow-up

Primary and secondary measures will be collected at baseline (pre-randomisation) and at 3, 6, 9, 12, 15, 18, 21 and 24 months (the majority of outcomes will not be collected at all of these timepoints). Liver disease severity score is collected at Months: 3, 6, 12, 18, 24. An additional visit at Day 30 will collect safety data (and the primary outcome if an event occurred).

8. Visit windows

Visits are scheduled every 3 months post-randomisation, with a visit window of +/-14 days.

9. Data collection

Please see the protocol for a detailed explanation of data collection.

10. Inclusion/Exclusion criteria

Inclusion criteria:

- Aged ≥ 18 years
- Confirmed alcohol-related (ALD) cirrhosis or metabolic dysfunction-associated steatotic liver (MASLD) or MASLD-ALD overlap cirrhosis based on clinical, radiological and/or histological criteria.
- MELD score 8-16
- Patients with alcohol-related cirrhosis must have an active alcohol consumption on average ≤ 20 grams/day
- Patients must be deemed to have the capacity to provide written informed consent to participate.

Exclusion criteria:

- Moderate, severe or life-threatening food allergy (*e.g.*, peanut allergy)
- Pregnancy or planned pregnancy. Urine testing will be performed at screening to rule out pregnancy in females.
- Breast-feeding.

- Patients treated for acute variceal bleeding, infection, overt hepatic encephalopathy, bacterial peritonitis or ACLF within 14 days prior to randomisation.
- Active alcohol consumption of >20 grams/day [1 unit of alcohol contains 10mLs or 8g of alcohol].
- Had a previous liver transplantation.
- Patients with inflammatory bowel disease.
- Patients with coeliac disease.
- Patients with a history of prior gastrointestinal resection or surgery that could change the gut microbiome or result in bacterial overgrowth e.g. gastric bypass.
- Active malignancy including hepatocellular carcinoma.
- Patients with an expected life expectancy <6 months or listed for liver transplantation.
- Infected with HIV, hepatitis B or C [patients who have undetectable hepatitis B or C DNA/RNA can be recruited].
- Patients who have received antibiotics or probiotics (excluding food stuffs containing 'live bacteria' such as live yoghurts, kefir, fermented vegetables such as sauerkraut/kombucha or cheese) within 7 days prior to randomisation.
- Swallowing disorder, oral-motor dyscoordination or likely inability/unwillingness to ingest study medication.
- Patients who have received another investigational drug or device within 4 months prior to randomisation.
- Patients, who in the opinion of the PI, have a medical condition, or other relevant psychological, familial, or social factor that may jeopardise their health, compliance, or influence the trial integrity in any way.

11. Measures

A detailed description of data collected will be given in the Schedule of Assessments and Measures (Section 7 of this document). Scoring for the primary and secondary outcomes will be provided in Section 8.

1.11.1 Measures collected at Baseline only

- Demographics; age, sex at birth, ethnicity, smoking status, BMI, index of multiple deprivation (IMD), country of birth, cirrhosis diagnosis, diabetes status, current or past history of psychiatric disorders, dietary information.

1.11.2 Primary outcome

- Defined infection or decompensating event resulting in hospital admission or presentation to ED (including day-cause paracentesis unit).

Infections and decompensating events which count for the primary outcome are defined above (in Section 3) and in the protocol.

Any participant that experiences the primary outcome will stop IMP and be requested to continue to be followed up for mortality and other secondary measures (if willing).

1.11.3 Secondary outcomes

The following secondary outcome measures will be covered by this Statistical Analysis Plan and form part of the Primary outcome paper:

1. Time to first infection resulting in hospitalisation over 24 month follow up period (former primary endpoint) (time to event).
2. Time to first decompensating event (time to event). All types of decompensating events will be included in a single outcome, but total counts will also be described separately, split into:
   i. hepatic encephalopathy,
   ii. new-onset or worsening ascites,
   iii. variceal bleeding and
   iv. spontaneous bacterial peritonitis.
3. Progression to ACLF i.e. the development of one or more organ failures (time to event)
4. Time to all-cause infection (time to event). All-cause infections will also be described by 6-, 12-, 18- and 24- month event rate.
5. Incidence of antibiotic usage (binary)
6. Occurrence of AMR (including skin and nose colonisation with methicillin-resistant Staphylococcus aureus (MRSA), vancomycin-resistant Enterococci (VRE), extended-spectrum beta-lactamase producing bacteria (ESBL), fluoroquinolone-resistant gram negative and linezolid-resistant *Enterococci* (LRE). Any occurrence of AMR will count towards this outcome, but types may be described separately (time to event).
7. Time to liver-related hospitalisation and all-cause hospitalisation (time to event). Length of stay (time to discharge among hospitalised participants) and admission to high dependency/intensive care (binary as to whether admitted to HDU/IC among those hospitalised)
8. Change in liver disease severity scores (scalars)
   i. Child Pugh
   ii. MELD
   iii. UKELD
9. Change in quality of life (EQ-5D-5L) scores (scalar)
10. Change in depression and anxiety scores (using HADS). (scalar)
11. All-cause mortality and liver-related mortality. (time to event)
12. Change in alcohol use disorder-related events in participants enrolled with alcohol-related cirrhosis as assessed by the alcohol-use disorders identification test (AUDIT score) (scalar)
13. Change in urinary ethyl glucuronide/ethyl sulphate levels if tested as part of the standard of care (scalar)

The following outcome measures will be mechanistic and not form part of the PROMISE Primary analysis, and out of scope of this analysis plan:

- **Matched faecal and plasma biomarkers** (D-lactate, fatty acid-binding protein 2 and ammonia) measured by enzyme-linked immunosorbent assay (ELISA).
- **Faecal proteases** as markers of DNA replication, tissue re-modelling and immune response as a proxy of gut mucosal barrier integrity.
- Matched **plasma and faecal cytokine profiles** using a combination of ELISA and by electrochemiluminescence using the Mesoscale discovery platform (MSD) [Tumour

necrosis factor-alpha, interferon-gamma, interleukin (IL)-1-beta, IL-6, IL-8, IL-10, IL-17A, IL-17E, IL-17F, IL-21 and IL-22].

- **Plasma, faecal and urinary metabonomics**. Short–chain fatty acids measured by proton magnetic resonance spectroscopy (^{1}NMR). Tryptophan metabolites and primary, secondary and tertiary bile acids measured with liquid chromatography tandem mass spectrometry (LC-MS) [Butyrate and Succinate].
- **Faecal proteomics** will identify protein changes using quantitative high-resolution mass spectrometry coupled with liquid chromatography LC-MS/MS analysis.
- Matched **faecal and blood microbiome composition** (including mycome, virome and phagome) and **microbial diversity** by metagenomic sequencing [20 million reads using NovaSeq] as a marker of bacterial translocation].
- **Faecal PCR for pathogenic species of interest** including copy number of DNA of cytolysin positive and negative *E. faecalis, enteropathogeneic E. coli* and *Candida albicans*.
- Analysis of the presence and dynamics of the **microbial 'resistome'** (ensemble of genes encoding AMR in a given microbiome using the comprehensive antibiotic resistance database [CARD]).
- The effect of FMT in cirrhosis on the human circulating immune profile will be explored in stored peripheral blood mononuclear cells (PBMCs) by assessing monocyte surface expression of CD16, HLA-DR, MerTK, Axl, CD163, PD-1, PD-L1, bile acid receptors TGR5 and FXR, transcriptomic profile and functional capacity (LPS-induced TNF-α, IL-6 and IL-10 production, phagocytosis and oxidative burst).
- Mucosa-associated invariant T (MAIT) cell numbers, phenotype and function isolated from PBMCs.
- Change in lipid profile (total cholesterol, triglycerides, HDL cholesterol, LDL cholesterol), and HbA1c in those with metabolic associated (MASLD) cirrhosis.

To interpret the faecal microbiome sequencing and metabolome analyses and how it impacts the participant's gut microbiome composition in relation to dietary habits

Sample size estimation (including clinical significance)

We assume 65% of participants in the control group experience an infection resulting in hospitalisation within 2-years and that this is reduced to 48.9% in the FMT arm. In order to detect this hazard ratio of 0.625, with 80% power (alpha = 0.047) we would need to analyse 270 participants (to experience 155 events). In order to account for a 10% loss to follow up, we will inflate to 300 participants (1:1 ratio). Note: 203 events would provide 90% power to detect this effect, and 261 events would provide 80% power to detect a HR = 0.70.

Using a group sequential design, an interim analysis will be carried out after 81 events (alpha = 0.003).

The sample size has been checked following KCTU-Statistics Standard Operating Procedures ST-05 and stored as signed as an essential document.

2 DATA ANALYSIS PLAN – DESCRIPTIVES

1. Recruitment and representativeness of recruited participants

A CONSORT flow chart will be constructed [12]– see Figure 1. This will include the number of eligible patients, number of patients consenting to enter the trial, number of patients refusing, then by treatment arm: the number continuing through the trial, the number withdrawing, the number lost to follow-up and the numbers excluded / analysed.

Figure 1 – Template CONSORT diagram

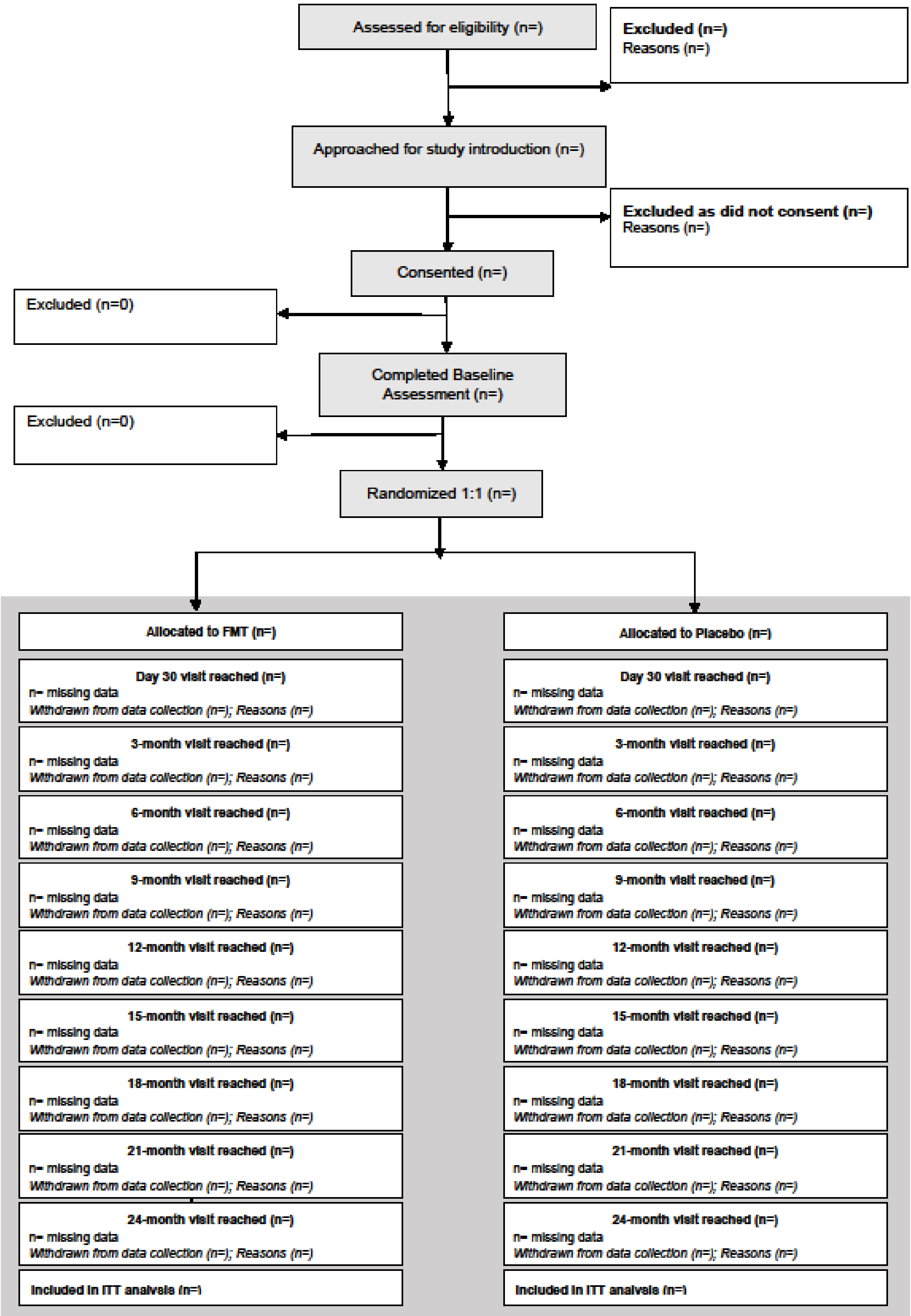

2. Baseline comparability of randomised groups

Baseline descriptions of participants by treatment and overall: means and standard deviation, medians and upper and lower quartiles, or numbers and proportions will be reported as appropriate.

No significance testing will be used to test baseline difference between the randomised treatment groups [5].

The following baseline variables will be described both overall and between randomised groups:

- Age
- Sex at Birth
- Ethnicity
- Country of birth
- Socioeconomic status (index of multiple deprivation)
- Smoking status
- Cirrhosis diagnosis
- Whether participant has diabetes

3. Anticipated intercurrent events

- Discontinuation of treatment (investigational medicinal product or placebo), estimated rate ≈10-20%
- Death, <5% expected
- Hospitalisation for non-infection or non-qualifying infection
- Hospitalisation for non-infection or non-qualifying infection and then contracting a nosocomial (qualifying) infection/decompensating event
- Liver transplant or being put on liver transplant waiting list (very low rates expected)
- Significant alcohol intake

Withdrawal from data collection (withdrawal of consent, uncontactable or unable to travel to assessment) also affects the outcomes as later measurements will be missing, but this affects the analysis and not the estimand so is not considered an intercurrent event.

4. Adherence to allocated treatment and treatment fidelity

Adherence to the IMP will be ascertained by number of capsules taken by the participant at each visit as recorded on the IMP Administration Log.

Formal adherence will be defined as having taken >70% of their capsules up until either reaching the end of the trial or experiencing the primary endpoint of hospitalisation due to infection.

Formal discontinuation of treatment will also be summarised by reason for discontinuation and by treatment arm using data from the IMP Administration Log.

5. Loss to follow-up and other missing data

It is the aim of the trial to minimise withdrawal of participants from treatment and follow-up. Completion of a withdrawal from trial is regarded as withdrawal from data collection/follow-up in this analysis; participants who discontinue treatment will still be followed up where possible.

Withdrawal from trial will be reported by intervention group. Moreover, the proportions of participants missing each variable will be summarised in each arm and at each time point. The numbers, proportions and reasons for withdrawal from trial will be summarised by treatment arm. The distribution of times between randomisation and withdrawal from follow-up will be summarised using a histogram.

The baseline characteristics of those missing follow-up will be compared to those with complete follow-up. Participants that experience the primary outcome event will be considered as completers. Data will be explored for pattern missingness.

6. Adverse event reporting

Adverse events (AE), adverse reactions (AR), serious adverse events (SAE) and serious adverse reactions (SAR) will be summarised by MEDDRA System Organ class, trial arm and time point. Adverse events will be reported cumulatively at each follow up timepoint. Additionally, risk ratios for adverse events by system organ class will be reported (using all participants' reported AEs post-randomisation *i.e.,* the ITT population) with 95% confidence intervals. These will be reported graphically in a dotplot in approach as proposed by Amit, Heiberger, and Lane (2008) (1) and implemented in Stata in the aedot package (2).

7. Assessment of outcome measures (planned unblinding of trial statistician)

Planned unblinding is based upon KCTU-Statistics SOP ST:06 Protocol Review and the protocol. All members of the research team will be blinded except the trial statistician who will be unblinded following approval of the first version of the SAP. The senior statistician will remain (fully) blind to post-baseline outcome data. The data monitoring committee (DMC) will be subgroup unblinded and reminded not to unblind members of the study team at the beginning of every meeting.

8. Blinding status of personnel and planned unblinding

All participants, Chief Investigator, Site Principal Investigators and outcome assessors will be fully blinded throughout the trial. The site pharmacy trials team, the nurse administering the treatment, and the trial manager will be unblinded to a participant's treatment allocation.

The Trial Statistician will be fully blind until the first version of the Statistical Analysis Plan (SAP) is approved by the Trial Steering Committee (TSC) as per KCTU SOP ST-02 (Statistical Analysis Plan). After the first version of the SAP is approved by the TSC, the Trial Statistician will become fully unblinded for the purpose of reporting at DMC meetings (for the closed report) and at interim analyses.

As per KCTU SOP ST-06, the Senior Statistician (and all other researchers) will be fully blind to treatment allocation until after completion of the final analysis.

9. Brief description of proposed analyses and any pre-analysis statistical checks required

Prior to database lock the statistician will perform data checks and generate queries for review by those responsible for entering data on the following:

- Ensuring that the CONSORT diagram can be completed with agreement between status forms/withdrawal forms and available data.
- Ensuring that all available primary and secondary outcome data has been entered by providing lists of pins of participants who do not have each measure entered.
- Checking inconsistencies between data entered in the trial database and data extracted from the randomisation system
- Ensure dates are coherent, e.g., time between date of hospitalisation and discharge is as expected
- Missing baseline data

10. Descriptive statistics for outcome measures

Each of the outcome measures will be described by treatment group. Means, standard deviations, or medians and interquartile ranges will be used for continuous variables. Frequencies and proportions will be used to describe categorical variables. Events will be described as the number occurred by each timepoint.

11. Listing of concomitant medications

The percentage of participants who are taking each type of medication (Antibiotics, Beta blockers, Probiotics, Anti-Depressants, Anti-Anxiety, Proton Pump Inhibitors only) will be described at baseline, 6, 12 and 24 months by trial arm.

3 DATA ANALYSIS PLAN – INTERIM ANALYSES

Using an asymmetric two-sided non-binding two-stage group sequential design with 90% power and 2.5% type-1 error, we will apply a lower bound cut-off rule to conclude futility and upper bound cut-off to determine superiority. After a minimum of 81 events, stage 1 will conclude futility if $Z<0.41$ (nominal p-value > 0.6499), favouring FMT, or superiority if $Z>2.75$ (H0: Reject, nominal $p<0.006$) favouring FMT. Applying type 1-error spending using HwangShih-DeCani and Gamma = -4 (one-sided, stage one spent error =0.003). Using this alpha spending approach, at the final analysis, the null hypothesis for efficacy will be rejected if $p<0.047$.

The interim analysis will include all randomised participants (eligibility as defined in Section 1.10) with outcome data in the arms to which they were randomised, regardless of what treatment they received post-randomisation. The interim will use all available follow up data at the cut-off timepoint for these participants. The formal between-group hypothesis test to assess efficacy will use an adjusted Cox regression model consistent with the primary outcome analysis. Kaplan-Meier survival curves will also be presented.

A second unblinded trial statistician will carry out the unblinded checks on the analysis code and results prior to generating the report following KCTU ST:07 Statistical Report sand KCTU Statistics SOPs ST:04- Statistical Quality Assurance. Meanwhile the Senior Statistician will remain fully blinded.

At the interim analysis, The DMC will provide advice to the TSC, who will advise the Sponsor in making the final decision on continuing or stopping the trial.

4 DATA ANALYSIS PLAN – INFERENTIAL ANALYSES

1. Primary analysis description

4.1.1 Definition of primary estimand

The primary clinical question of interest is what is the difference in time until first infection (as defined in Section 3) OR first decompensating event (as defined in Section 1.3) resulting in hospitalisation or presentation to ED for participant's with alcohol-related and Metabolic dysfunction-Associated Steatotic Liver Disease (MASLD) cirrhosis (as defined by trial inclusion/exclusion criteria per Section 1.10) over a maximum of 24 months follow up, treated with FMT compared to Placebo (5 capsules once daily), regardless of discontinuation of treatment or presence of other intercurrent events whilst participants are alive (as described below).

The estimand is described by the following attributes:

- **Population**: People with alcohol-related cirrhosis (ALD), metabolic dysfunction-associated steatotic liver disease (MASLD), or Alcohol -related Liver Disease Overlap (MASLD-ALD Overlap), (as defined by trial inclusion/exclusion criteria).
- **Endpoint**: Composite endpoint of first infection or decompensating event resulting in hospitalisation over a maximum of 24 months follow-up.
- **Treatment condition**: FMT compared to placebo regardless of discontinuation for any reason (treatment policy strategy).
- **Remaining intercurrent events:** The intercurrent event "intervention discontinuation" is already addressed by the treatment condition of interest attribute. Other non-terminating intercurrent events will also use a treatment policy strategy. Death is a terminating intercurrent event that will use a while alive strategy. These are further described in Section 4.1.2.
- **Population-level summary:** The adjusted hazard ratio (ratio of the hazard rate of first infection resulting in hospitalisation between treatment conditions).

Rationale for estimand: To compare FMT against placebo as would be observed in routine practice for participants who remain alive.

4.1.2 Further details on handling of intercurrent events and subsequent supplementary analyses

As stated, for the primary analysis, a treatment policy strategy will be used for the ICEs (intercurrent events) of discontinuation of treatment and other non-terminating events other than liver transplantation (as listed in Section 2.3); that is the occurrence of the

intercurrent event will be considered irrelevant in defining the treatment effect of interest and the value for the variable of interest will be used regardless of whether or not the intercurrent event occurs. Separate supplementary analyses using different strategies will be carried out for these ICEs if they have non-trivial rates of occurrence that will target different estimands.

Death is a terminating intercurrent event for the primary outcome. The outcome will be censored at time of death for the primary analysis and therefore the primary analysis will use a while-alive strategy for this intercurrent event (that is the estimand will be for participants who do not die). A supplementary analysis will be carried out if there are non-trivial numbers of liver related deaths, this will use an alternative composite strategy, where the outcome will be defined as "liver related death or time to first hospitalisation for qualifying infection" and targets a different estimand.

A while-alive strategy will also be employed for liver transplantation. The outcome will be censored at time of liver transplant for the primary analysis – the estimand will be for participants who do not have a liver transplant. A supplementary analysis will be carried out if there are non-trivial numbers of liver transplant (an event rate of 7.5% or higher), where a composite strategy will be used (i.e. the outcome will be time to first hospitalisation for qualifying infection OR time to liver transplantation).

For ICEs that remove the participant from risk of the primary outcome i.e. hospitalisation due to other reasons (or non-qualifying infection) or nosocomial infection/decompensating event, a composite strategy is suitable. However, these are equivalent to secondary outcomes (all cause hospitalisation, all cause culture confirmed infections, incidence of decompensating events) for which analyses are already being carried out.

A composite strategy will also be used combining the ICE of being put on the liver transplant waiting list if there are non-trivial rates of these ICEs.

2. Main analysis of treatment differences (intention-to-treat population set)

The main analysis will include all randomized participants (eligibility as defined in section 1.10) in the arms to which they were randomized, regardless of what treatment they received post-randomisation, with the intent to estimate an intention-to-treat (ITT) type estimand. This Modified ITT population will be estimated for the primary outcome and all secondary outcomes and will include: all participants who return to at least one of the assessment time-points, or those confirmed as alive and without hospitalisation and presentation to the ED of any cause, or those confirmed hospitalised/presented to ED due to infection or decompensating event (via hospital record or telephone call).

3. Per protocol population set

As per Section 1, the aim of the main analysis is to estimate an Intention to treat (ITT) type estimand. A separate analysis will also be carried out using a per-protocol population set to estimate efficacy of the intervention (Average treatment effect in the treated) in ideal conditions. The per-protocol population will be the same as the ITT population except including only participants who adhered to the treatment schedule (as defined in Section 4) and excluding other protocol violators. Protocol deviators as defined below will not be excluded.

4.3.1 Definition of protocol deviation/violation

A protocol deviation/violation is defined as any excursion from the protocol. Examples of possible protocol deviations/violations include participants who do not meet the eligibility criteria and are randomised in error, participants receiving the incorrect treatment allocation and participant assessments occurring outside the specified visit windows.

A protocol deviation (PD) is defined as a non-serious breach from the protocol that is unlikely to lead to any impact on the value of the data contributing to the overall treatment effect. A protocol violation (PV) is an excursion from the protocol that is likely to lead to an impact on the estimate of the "efficacy" of the intervention. Protocol violations will be reported (and tabulated by arm).

All visit window deviations will be classified as protocol deviations rather than protocol violations as the primary analysis uses actual time since randomisation and therefore deviations from the visit windows are not impactful for this analysis. An assessment of the impact of other excursions from the protocol on the primary outcome will be made by the Chief investigator and Senior statistician. All excursions from the protocol will be reported to the DMC and they will have final decision on which should be considered protocol violations (rather than deviations).

4. Analysis of primary outcomes

4.4.1 Primary Efficacy Parameters

We will model the time to first hospitalisation or presentation to ED due to defined infection or decompensating event, between participants allocated to FMT and placebo using a multivariable Cox proportional baseline hazards regression model, with a frailty (random intercept) for site. The analyses will be adjusted for type of cirrhosis (alcohol related, MASLD, both), participant's age, sex at birth, baseline cirrhosis severity (baseline MELD score), and whether the participant has diabetes (yes/no).

We will present the crude Hazard Ratio (HR), i.e., not adjusted for covariates as above and adjusted Hazard Ratio (aHR) i.e., adjusted for covariates as above. The adjusted hazard ratio will be considered the primary result. A significance level=0.047 will be used for the primary analysis between-group difference for the aHR (accounting for alpha spending approach for the interim analysis), alongside the 95% confidence interval (95%CI).

Log-log plots will be used to visually assess the proportional hazards assumption. If the proportional hazards assumption appears to be violated, we will examine how the hazard ratio changes over time using an interaction between trial arm and time in the model and report the results from this model accordingly.

A Kaplan-Meier plot will also be generated with an associated log-rank test and the adjusted Hazard Ratio (with confidence intervals and p-value) presented as part of the plot.

5. Analysis of secondary outcomes

All secondary analyses will use a significance level=0.05.

4.5.1 CONTINUOUS OUTCOMES

Continuous outcomes (liver disease severity scores; AUDIT score; HADS score; EQ-5D-5L score) will be analysed with (longitudinal) multi-level mixed-effects multivariable linear regression models using all follow up time-points. Time will be included as a continuous covariate using actual time of measurements post-randomisation, and post-estimation commands will be used to get estimates at specific follow up times. A random intercept and slope over time will be fitted by participant and the analysis adjusted by terms consistent with the primary outcome as well as baseline values of the outcome where appropriate. Unadjusted and adjusted results will be presented.

4.5.2 BINARY OUTCOMES

Binary outcomes (antibiotic usage) will be analysed with (longitudinal) multi-level mixed-effects logistic regression models, at 12- and 24-months post-randomisation. Time will be included as a categorical covariate using actual time of measurements post-randomisation, and post-estimation commands used to get estimates at specific follow up times. A random intercept will be fitted for participant and the analysis adjusted by terms consistent with the primary outcome as well as baseline values of the outcome where appropriate. Unadjusted and adjusted analyses will be presented.

The binary outcome of HDU/Intensive care amongst hospitalised participants will use a logistic regression model. The analysis adjusted by terms consistent with the primary outcome; unadjusted and adjusted results will be presented.

4.5.3 TIME TO EVENT OUTCOMES

Time to event outcomes (mortality (all-cause; liver-related), hospitalisation (all-cause; liver-related; time to discharge), infection, AMR incidence, progression to ACLF and hepatic decompensation events) will be analysed in the same way as the primary outcome using a multivariable Cox regression model. The analyses will be adjusted for type of cirrhosis (alcohol related, MASLD, both), participant's age, sex at birth, diabetes status (yes/no) and baseline cirrhosis severity as fixed effects with a frailty term (random intercept) for site as per the primary analysis. We will present unadjusted and adjusted analyses.

Log-log plots will be used to visually assess the proportional hazards assumption. If the proportional hazards assumption appears to be violated, we will examine how the hazard ratio changes over time using an interaction between trial arm and time in the model.

6. Safety

No additional safety outcomes will be analysed other than those covered by existing secondary outcomes or descriptives of AEs (risk ratios by system body class) as in Section 6.

7. Time points

As per Section 8, Visit windows exist for all follow up visits. However, all available data will be used for the main analyses regardless of compliance to visit windows.

8. Stratification and clustering

Randomisation has been stratified by site. Therefore, it is important to include this variable in the modelling process. Site at randomisation will be adjusted for in all relevant analyses.

The structure of some of the secondary outcome data is longitudinal with repeated measurements. This correlation of observations within participants will be accounted for in the modelling process by including a subject-varying random intercept.

9. Missing items in scales and subscales

The number (%) with complete data will be reported. If missing data guidance is provided for missing items within the scale scoring guide, this will be used. If no guidance is provided scales will be pro-rated for an individual if 20% or fewer items are missing. For example, in a scale with 10 items, prorating will be applied to individuals with 1 or 2 items missing. The average value for the 8 or 9 complete items will be calculated for that individual and used to replace the missing values. The scale score will be calculated based on the complete values and these replacements.

10. Missing baseline data

If there is missing baseline data for an outcome, an approach such as including a missing indicator for baseline as a covariate in the model may be used as per the recommendations of White et al [7] .

11. Missing outcome data

For secondary binary and continuous outcomes, the mixed model will be fitted using maximum likelihood methods that are valid under the MAR assumption. However, this assumption relates to the variables that are included in the model. To allow for a variable which predicts missingness, this variable needs to be included as either one of the explanatory or dependent variables in the model. Demographic or clinical baseline variables (those listed in Section 2) will be investigated as predictors of missingness. Should any of these variables be predictors of outcome missingness then such variables will be included as covariates in the model if deemed suitable for adjustment.

If post treatment variables such as compliance with treatment, relapse to alcohol, or baseline variables not suitable to adjust for in the main analyses, are found to be predictive of drop out, inverse probability weighting will be considered to account for these variables.

12. Method for handling multiple comparisons

No adjustment will be considered for multiple comparisons, allowing reviewers to make their own adjustment to estimates if they wish [8].

13. Method for handling non-compliance

Compliance for the purposes of analysis will be defined as having taken >70% of IMP capsules up until either reaching the end of the trial or experiencing the primary endpoint (or terminating intercurrent event such as death). Participants will receive five capsules of the Investigational Medicinal Product (IMP) to be taken orally at a hospital clinic every 3 months +/- 14 days for a total of 21 months. This equates to an expected total of 40 capsules over course of trial if they complete all study medication.

Compliance will be summarised and if there is considerable non-compliance, we will look at estimating the average treatment effect for the subpopulation of compliers using a complier average causal effect (CACE) estimand.

We will not account for patterns of compliance (i.e., compliance over time) in such an analysis unless the data suggests large variation in such patterns. As capsules are administered at the visit, we expect participants to almost always take all 5 capsules at the visit. We don't expect many participants to miss taking capsules and then resume taking capsules later.

14. Model assumption checks

For each time-to-event outcome the Cox proportional hazard model assumption will be checked visually using a log-log plot using Schoenfeld residuals. During the analysis, the blinded senior statistician will examine such residuals whilst remaining blinded to trial arm. If there is any concern as to the proportional hazards assumption being violated, a different model will be used with approval from the unblinded statistician on the Trial Steering Committee.

The models assume normally distributed error terms (residuals); this will be checked following model fit and if these appear non-normal, transformations or bootstrapping of the standard errors (and subsequent confidence intervals) will be considered.

15. Sensitivity analyses

The following sensitivity analyses will be carried out for the primary analysis:

- The impact of departures from the missing at random (MAR) assumption on treatment effects will be assessed using pattern mixture models (3) to give results for a range of scenarios for the possible unobserved values.
- If relevant, the impact of any changes to the inclusion/exclusion criteria during the trial will also be assessed using a sensitivity analysis/analyses; looking at effects within subgroups (by including an interaction term) as defined by different eligibility criteria over time.
- If relevant, the impact of alcohol will be assessed by excluding those who have significant alcohol consumption.

16. Planned subgroup analyses

The following subgroups will be used to explore for moderating effect:

- sex (male, female)
- participant's age
- type of cirrhosis (alcohol-related, MASLD or overlap)
- socioeconomic status (using the index of multiple deprivation)
- additional factors determined by the Trial Steering Committee.

Subgroup analyses will be conducted by including an interaction term (subgroup*trial arm*time) in the model.

5 Quality Control and Quality Assurance

This trial is conducted by KCTU using the KCTU Quality Management systems including Incident reporting, KCTU SOPs and the Statistics File Upload System for storing essential statistical documents. Quality control and assurance is maintained following KCTU-Statistics ST:04 Statistical Quality Assurance.

1. Quality control

In addition to other data checking carried out during the trial to ensure data quality (including source data verification by the KHP-CTO and data cleaning by trial management team as outlined in the protocol), the junior statistician will carry out data checks on MACRO data prior to preparing DMC reports (as per KCTU Statistical SOP ST-01), prior to interim analyses and prior to final data lock. Generated data queries will be provided to the trial management team in an Excel file to be communicated to sites. Responses should be sent back to the statistician and unresolved data queries will be re-issued. Where possible data queries should be resolved prior to DMC reports/interim analyses, where this is not possible, outstanding data issues will be made clear to the DMC on the report. Syntax for data checking will be written in accordance with KCTU Statistical SOP ST-08 (Data manipulation) to allow reproducibility and for purposes of an audit trail. All syntax, output, resulting queries and responses from sites (via the trial management team) will be retained as per KCTU Statistical SOP ST-03 (Statistical Document Retention).

2. Internal Pilot 'Go/No Go' progression

At the end of one year after the first site has opened for recruitment, we will assess the feasibility of the trial in an internal pilot. The criteria are shown below in the table in green.

At the end of one year after the first site has opened to recruitment, we will assess the recruitment rate and the feasibility of continuing the PROMISE Trial. We will progress to the full trial by meeting the following 'go/'no go' criteria, as indicated by meeting the green criteria: *<80% will result in being flagged as amber and less than 64% red.	*Red	*Amber	Green
Open eight sites	<6	6 to 7	8 or more
Randomise 80 participants	≤64	65-79	80 or more
Participant adherence (% of patients)	≤64	65-79	80 or more

3. Carrying out interim analysis and final analysis

The interim analysis will be carried out by the Trial Statistician and the syntax for the interim analysis will be checked by the Senior TS following the KCTU Statistical SOP ST-08 (Data manipulation). Code will be written such that will not unblind the Senior Trial Statistician and the Senior TS will not see the trial results or trial arm allocation data. A dummy trial arm variable may be created to allow the trial statistician to see format of results.

Similarly, for the final analysis, the Senior TS will review the code (that will be written in such a way as to not unblind) but will not see results until finalisation of the analysis.

As per KCTU Statistical SOP ST-08, all syntax will be written in such a way to allow for the analysis to be reproduced for purposes of an audit trail, and syntax will be appropriately commented to allow interpretation and for reproducibility. All syntax and output will be retained on local filing systems and uploaded to the Statistics File Upload System (as per KCTU Statistical SOP ST-03 (Statistical Document Retention)).

4. Approval and version control of the SAP

As per KCTU Statistical SOP ST-02 Statistical Analysis Plan, after initial sign off of the SAP, if any changes are required, these will be decided by the blinded senior TS and not by the unblinded trial statistician, to avoid any chance or appearance of introducing bias having seen unblinded data. A copy of the tracked changes version will be retained, and the updated version will be approved by the Chief Investigator, Senior Statistician and TSC chair. Any changes between versions will also be outlined in Section 11.

The initial signed off version will be versioned as Version 1.0. Minor amendments following this should be versioned with sub-numbering e.g., Version 1.1, Version 1.2 etc. Major amendments should be numbered as Version 2.0, 3.0 etc.

5. Quality Assurance

Quality assurance processes will be followed according to KCTU Statistical SOP ST-04 Statistical QA.

6 SOFTWARE

Data management: An online data collection system for clinical trials (MACRO; Elsevier) will be used. This is hosted on a dedicated server at KCL and managed by the King's CTU. The KCTU will extract data periodically as needed and provide these in comma separated (.csv) or Stata (.dta) format.

Statistical analysis: Stata (version 17 or higher) will be used for the main analyses. R (version 3.6.3 or higher) and R markdown may also be used for producing graphs and tables and automating report writing.

7 SCHEDULE OF ASSESSMENTS AND MEASURES

Data being collected from participant's and recorded in MACRO:

	Screening (Day -28 to Day 0)	Baseline (Day -7 to Day 0)	**Screening & Baseline Repeat** [b]	Randomisation (Day 0)	Post-randomisation IMP dispensing (Day 0 + 1 day)	Day 30 +/- 7 days	Month 3 +/- 14 days	Month 6 +/- 14 days	Month 9 +/- 14 days	Month 12 +/- 14 days	Month 15 +/- 14 days	Month 18 +/- 14 days	Month 21 +/- 14 days	Month 24 +/-14 days	**Primary Endpoint**
Informed consent	X														
Eligibility confirmation	X		X												
Urinary β-HCG test (females of childbearing potential) [a]	X	X	X												
BMI		X													
Randomisation				X											
IMP dispensing					X		X	X	X	X	X	X	X		
IMP accountability					X		X	X	X	X	X	X	X		
IMP administration					X		X	X	X	X	X	X	X		
IMP compliance					X		X	X	X	X	X	X	X		
Nurse-led telephone call (safety)						X									
Adverse event monitoring	X	X	X	X	X	X	X	X	X	X	X	X	X	X	X

Physical Examination		X					X	X	X	X	X	X	X	X	X
Primary Endpoint															X
Concomitant medications check		X	X			X	X	X	X	X	X	X	X	X	
Dietary Questionnaire		X													
**Duration of abstinence from alcohol**			X												
Routine haematology, biochemistry, glucose, clotting (INR) and C-reactive protein<br><br>(Standard of care [d])		X	X				X	X		X		X		X	
Study blood sampling		X					X	X		X					
Study urine sampling		X					X	X		X					
Study stool sampling		X					X	X		X					
Serum sample<br><br>(for archiving)		X													
HbA1C<br><br>(for diabetic participants only)		X					X	X		X		X		X	X
Lipid Profile (for MASLD and MASLD-ALD Overlap Cirrhosis participants only)		X					X	X		X		X		X	X

Liver disease severity score (MELD, Child Pugh, UKELD)		X	X				X	X		X		X		X	X
Quality of life questionnaire (EQ-5D-5L)		X					X	X		X				X	
AUDIT questionnaire (for alcohol-related and MASLD-ALD Overlap cirrhosis participants only)		X					X	X		X				X	X
**Urinary ethyl glucuronide / Ethyl sulphate test**<br><br>(For alcohol-related and MASLD-ALD Overlap cirrhosis participants only)<br><br>(Only if part of standard of care) [c]		X					X	X		X				X	X
**Assessment of Alcohol Intake**										X				X	X
Hospital and Anxiety Depression Scale (HADS)		X					X	X		X				X	

[a] When screening and baseline visits are performed on the same day, the Urinary β-HCG test (females of childbearing potential) will only need to be performed once for eligibility confirmation.

[b] These procedures must be repeated if time between baseline visit and randomisation is more than 7 days. If the time between baseline and randomisation elapses over 28 days, the full screening and baseline visit procedures must be repeated.

[c] Only performed if this is a part of the participant's standard of care routine.

[d] Routine standard of care bloods must include INR and C-reactive protein testing.

8 SCORING

Outcome	Title	Scoring present?	Timescale/Type	Items	Scoring description	Missing items	Interpretation	Reference
**Primary outcome**	**Time to the first infection resulting in hospital admission.**	NA	Time to first infection with hospitalisation	NA	Time to first infection with hospitalisation	NA	Higher odds/risk/hazard worse	NA
**Secondary outcome**	To evaluate the efficacy of encapsulated FMT to reduce **decompensating cirrhotic events**: (i) acute variceal bleeding	NA	Recurrent event	NA	Whether acute variceal bleeding occurs by 24 months.	NA	Higher odds/risk/hazard worse	NA
**Secondary outcome**	(ii) new-onset ascites	NA	Recurrent event	NA	Whether new-onset ascites occur by 24 months.	NA	Higher odds/risk/hazard worse	NA
**Secondary outcome**	iii) spontaneous bacterial peritonitis	NA	Recurrent event	NA	Whether spontaneous bacterial peritonitis occurs by 24 months.	NA	Higher odds/risk/hazard worse	NA
**Secondary outcome**	(iv) hepatic encephalopathy	NA	Recurrent event	NA	Whether hepatic encephalopathy occurs by 24 months.	NA	Higher odds/risk/hazard worse	NA
**Secondary outcome**	(v) development of acute-on-chronic liver failure (the development of	NA	Time to development of liver failure	NA	Time to development of liver failure	NA	Higher odds/risk/hazard worse	NA

	one or more organ failures)							
**Secondary outcome**	To assess the impact of encapsulated FMT on (i) **antibiotic usage episodes** and	NA	Recurrent event	NA	Whether antibiotic usage episodes occur by 24 months	NA	Higher odds/risk/hazard worse	NA
**Secondary outcome**	(ii) development of antimicrobial resistance	NA	Time to antimicrobial resistance	NA	Time to antimicrobial resistance	NA	Higher odds/risk/hazard worse	NA
**Secondary outcome**	To evaluate the impact of encapsulated FMT on **all-cause hospitalisation** documenting **(i) length of stay**	NA	Time to all-cause hospitalisation	NA	Time to all-cause hospitalisation	NA	Higher odds/risk/hazard worse	NA
**Secondary outcome**	To evaluate the impact of encapsulated FMT on **all-cause hospitalisation** documenting **(i) length of stay** and **(ii) requirement for high dependency and intensive care.**	NA	Time to high dependency or intensive care	NA	Time to high dependency or intensive care	NA	Higher odds/risk/hazard worse	NA

**Secondary outcome**	Liver disease score - Child Pugh	Yes	At visit	5	4-15 points, based on<br>Encephalopathy: None = 1 point, Grade 1 and 2 = 2 points, Grade 3 and 4 = 3 points<br>Ascites: None = 1 point, slight = 2 points, moderate or severe = 3 points<br>Bilirubin: under 34 umol/L = 1 point, 34-50 umol/L= 2 points, over 50 umol/L = 3 points<br>Albumin: greater than 35 g/L = 1 point, 28 to 35 g/L = 2 points, less than 28 g/L = 3 points<br>INR:<1.7 = 1 point, 1.71-2.30=2 points, >2.30 = 3 points	No guidance		(4)
**Secondary outcome**	Liver disease score - MELD	Yes	At visit	3	Score based from 6-40:<br>9.57 × ln(creatinine, mg/dl) + 3.78 × ln(total bilirubin, mg/dl) + 11.2 × ln(INR) + 6.43<br><br>Bilirubin will need to be first converted from umol/L to mg/dl by dividing by 17.1<br><br>Creatinine will need to be first converted from umol/L to mg/dl by dividing by 88.4	No guidance		(5)
**Secondary outcome**	Liver disease score - UKELD	Yes	At visit	4	The UKELD score is calculated from the participant's INR, serum creatinine, serum bilirubin and serum sodium, according to the formula:[3]<br><br>5.395 x ln[INR]) + (1.485 x ln creatinine, umol/L) + (3.13 x ln bilirubin, umol/L) - (81.565 x ln Na, mmol/L) +435	No guidance		(6)

**Secondary outcome**	EQ-5D-5L	Yes	At assessment	5 + VAS	EQ5D-5L health states (the 5 domains) will be converted into an index value as per the EQ5D-5L scoring guide by applying the Devlin formula that attaches values (weights) to each of the levels in each dimension. The Devlin set will be used to calculate as this measure is assessing quality of life. Mean VAS scores will also be compared.	No guidance specified	Higher scores worse on sum of health states. Higher scores better on VAS.	(7) (8)
**Secondary outcome**	liver-related mortality	NA	Time to liver related mortality	NA	Time to liver related mortality	NA	Higher odds/risk/hazard worse	NA
**Secondary outcome**	Change in alcohol use disorder-related events (alcohol related cirrhosis only) - AUDIT score	Yes	At assessment	10	Scores for each question range from 0 to 4, with the first response for each question (eg never) scoring 0, the second (eg less than monthly) scoring 1, the third (eg monthly) scoring 2, the fourth (eg weekly) scoring 3, and the last response (eg. daily or almost daily) scoring 4. For questions 9 and 10, which only have three responses, the scoring is 0, 2 and 4 (from left to right).	No guidance specified	Higher score is worse. A score of 8 or more is associated with harmful or hazardous drinking, a score of 13 or more in women, and 15 or more in men, is likely to indicate alcohol dependence	(9)
**Secondary outcome**	Hospital and Anxiety Depression Scale (HADS)	Yes	At assessment	14	Each item scored 0-3 and items totalled. Some items reversed (1,3,5,6,8,10,11). Two separate scores, one for depression and one for anxiety.	No guidance specified	Higher scores worse	(10)

9 DUMMY TABLES FOR FINAL ANALYSIS

The below tables are examples only and may not be fully populated, for example, for the Site stratifier, this may not be a complete list.

Descriptive baseline characteristics

Table 1 - Baseline characteristics/Demographics

Baseline characteristics		FMT n= XX	Placebo n= XX
Age	Mean(SD), range		
Sex at Birth	Female		
	Male		
Ethnicity	White		
	Mixed or Multiple ethnic groups		
	Asian or Asian British		
	Black, African, Caribbean or Black British		
	Other ethnic group		
Smoking status	Never smoked		
	Former smoker		
	Current smoker		
Index of multiple deprivation (decile)	Mean(SD), range		
Country of birth	United Kingdom		
	Europe (including Russia)		
	South Asia		
	East Asia		
	Africa		
	Caribbean		
	Other		
Confirmed cirrhosis diagnosis	Alcohol-related (ALD)		
	Metabolic dysfunction-Associated Steatotic Liver Disease (MASLD)		
	MASLD-ALD Overlap cirrhosis		
Whether participant has diabetes	Yes – Type I		
	Yes – Type II		
	No		

Table 2 - Baseline Characteristics/Stratification factors

Baseline Characteristics		Total N (%)	FMT n (%)	Placebo n (%)
Stratification factor – Site	King's College Hospital NHS Foundation Trust			
	St. George's University Hospital NHS Foundation Trust			
	Aneurin Bevan University Health Board			
	Gateshead Health NHS Foundation Trust			
	Imperial College Healthcare NHS Foundation Trust			
	Leeds Teaching Hospitals NHS Foundation Trust			
	Newcastle Upon-Tyne NHS Foundation Trust			
	Nottingham University Hospital Foundation Trust			
	University Hospitals of Derby and Burton NHS Foundation Trust			
	Royal Liverpool and Broadgreen Hospitals NHS Trust			
	Glasgow Royal Infirmary			
	Ninewells Hospital, University of Dundee			

Table 3 - Baseline clinical scales

Scale	Sample mean (SD) or N (%)	FMT	Placebo
Child Pugh Score			
MELD score			
UKELD score			
AUDIT score			
Hospital and Anxiety Depression Scale (HADS)			
HADS Depression			
HADS Anxiety			

Primary outcome

Table 4 - Primary outcome results

	Number of events (%)			
**Outcome**	**FMT**	**Placebo**	**Hazard Ratio (SE)**	**p-value (95% CI)**
First infection resulting in hospitalisation				

Figure 1 - Kaplan Meier plot for primary outcome

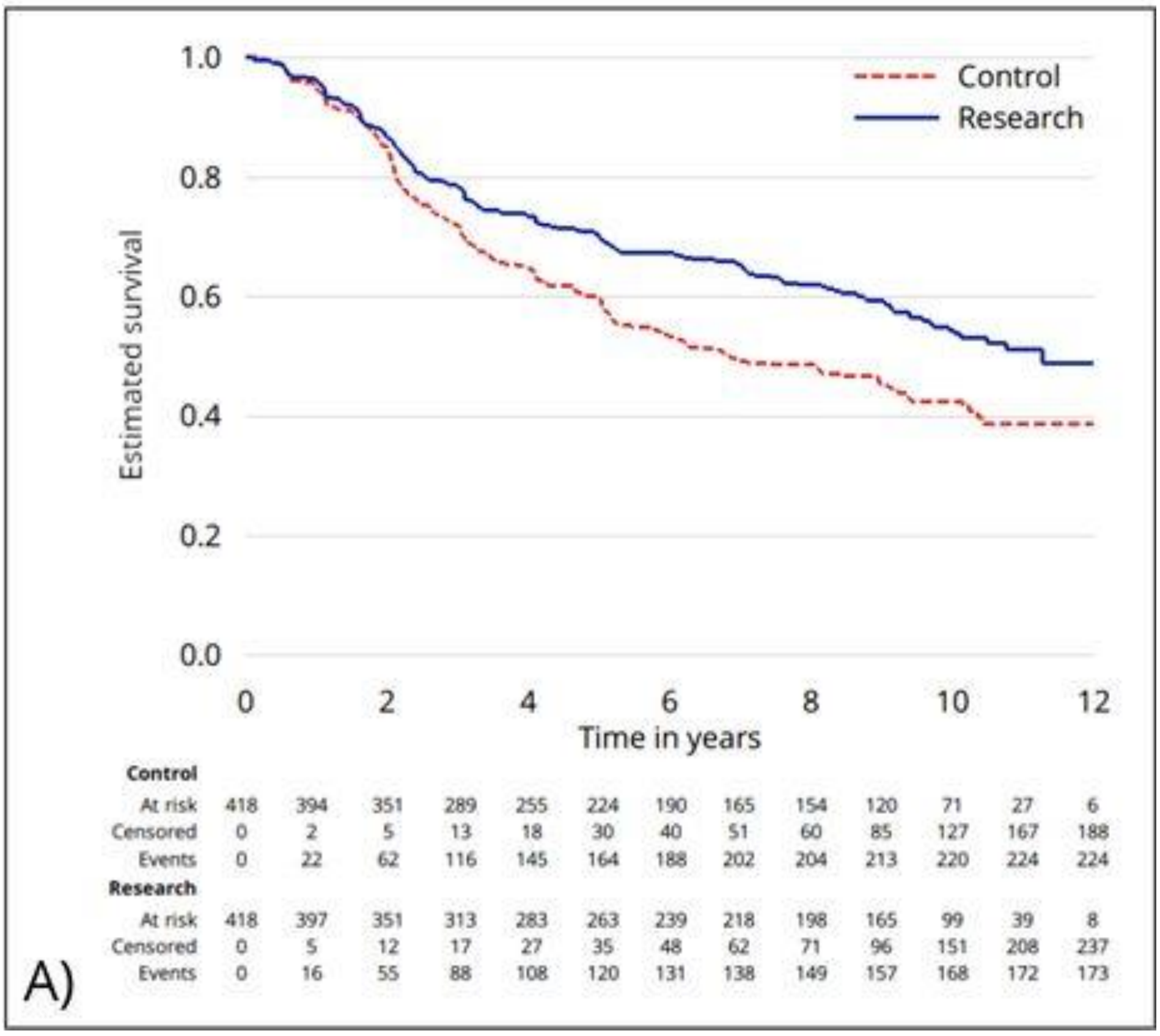

This is an example for purposes of showing proposed formatting only.
(used and modified as per Morris et al (11) under a Creative Commons Attribution 4.0 Unported (CC BY 4.0) license (https://creativecommons.org/licenses/by/4.0/))

Intercurrent events

Hazard ratios in the below table will be for the sensitivity analysis for all-cause mortality accounting for the intercurrent event if such an analysis is carried out (depending on number of events).

Table 5 - Intercurrent events

**Event**	**Number of people (%)**		**Number of events (%)**		**Adjusted Hazard Ratio (SE)**	**p-value (95% CI)**
	**FMT**	**Placebo**	**FMT**	**Placebo**		
Death						
Liver transplant waitlist						
Liver transplant						
Hospitalisation for non-infection or non-qualifying infection						
Hospitalisation for non-infection or non-qualifying infection and then contracting a nosocomial (qualifying) infection						

Secondary outcomes

Table 6 - Secondary outcome results

Outcome	Unadjusted, Mean (SD)		Adjusted Ratio/Odds Ratio/Adjusted Difference (SE)	Hazard Mean	p-value (95% CI)	Cohen's d (if applicable) (95% CI)
	FMT	Placebo				
Outcome:*						
3 months	*n*= XX	*n*= XX				
6 months	*n*= XX	*n*= XX				
9 months	*n*= XX	*n*= XX				
12 months	n= XX	n= XX				
15 months	n= XX	n= XX				
18 months	n= XX	n= XX				
21 months	n= XX	n= XX				
24 months	n= XX	n= XX				

Compliance

Table 7 - compliance/adherence to randomised intervention

Adherence to randomised intervention	FMT n (%)	Placebo n (%)	Total N (%)
Complied to treatment (includes those who had an event or death and complied to that point as per definition of compliance)	0/0 (%)	0/0 (%)	0/0 (%)
Completed treatment to 21 months	0/0 (%)	0/0 (%)	0/0 (%)
**Withdrawn from treatment**	0/0 (%)	0/0 (%)	0/0 (%)
Reasons			
1. Participant met the primary endpoint (ensure Index Hospitalisation form is completed)	0/0 (%)	0/0 (%)	0/0 (%)
2. Other Adverse Event	0/0 (%)	0/0 (%)	0/0 (%)
3. Participant decision unrelated to Adverse Event	0/0 (%)	0/0 (%)	0/0 (%)
4. Participant had a liver transplant	0/0 (%)	0/0 (%)	0/0 (%)
5. Other	0/0 (%)	0/0 (%)	0/0 (%)

Safety/Adverse events Reporting

NSAE = Non Serious Adverse Event SAE = Serious Adverse Event. NSAR = Non Serious Adverse Reaction, SAR = Serious Adverse Reaction

Table 8 - Number of adverse events by timepoint

	FMT		Placebo	
**Timepoint**	**NSAEs (ARs)**	**SAEs (SARs)**	**NSAEs (ARs)**	**SAEs (SARs)**
3 months	*n*= XX (n=XX)	*n*= XX (n=XX)	*n*= XX (n=XX)	*n*= XX (n=XX)
6 months	*n*= XX (n=XX)	*n*= XX (n=XX)	*n*= XX (n=XX)	*n*= XX (n=XX)
9 months	*n*= XX (n=XX)	*n*= XX (n=XX)	*n*= XX (n=XX)	*n*= XX (n=XX)
12 months	*n*= XX (n=XX)	*n*= XX (n=XX)	*n*= XX (n=XX)	*n*= XX (n=XX)
15 months	*n*= XX (n=XX)	*n*= XX (n=XX)	*n*= XX (n=XX)	*n*= XX (n=XX)
18 months	*n*= XX (n=XX)	*n*= XX (n=XX)	*n*= XX (n=XX)	*n*= XX (n=XX)
21 months	*n*= XX (n=XX)	*n*= XX (n=XX)	*n*= XX (n=XX)	*n*= XX (n=XX)
24 months	*n*= XX (n=XX)	*n*= XX (n=XX)	*n*= XX (n=XX)	*n*= XX (n=XX)

Table 9 - All adverse events after 24 months follow up

"n participants" is number of unique participants that has experienced at least one of those type of adverse events.

	Arm A		Arm B		Total	
**Body System Code**	**Number of NSAEs (n participants, %)**	**Number of SAEs (n participants, %)**	**Number of NSAEs (n participants, %)**	**Number of SAEs (n participants, %)**	**Number of NSAEs (n participants, %)**	**Number of SAEs (n participants, %)**
1. Infections and infestations	n= (n/n, %)	n= (n/n, %)	n= (n/n, %)	n= (n/n, %)	n= (n/n, %)	n= (n/n, %)
2. Neoplasms benign, malignant and unspecified (incl cysts and polyps)	n= (n/n, %)	n= (n/n, %)	n= (n/n, %)	n= (n/n, %)	n= (n/n, %)	n= (n/n, %)
3. Blood and lymphatic system disorders	n= (n/n, %)	n= (n/n, %)	n= (n/n, %)	n= (n/n, %)	n= (n/n, %)	n= (n/n, %)
4. Immune system disorders	n= (n/n, %)	n= (n/n, %)	n= (n/n, %)	n= (n/n, %)	n= (n/n, %)	n= (n/n, %)
5. Endocrine disorders	n= (n/n, %)	n= (n/n, %)	n= (n/n, %)	n= (n/n, %)	n= (n/n, %)	n= (n/n, %)
6. Metabolism and nutrition disorders	n= (n/n, %)	n= (n/n, %)	n= (n/n, %)	n= (n/n, %)	n= (n/n, %)	n= (n/n, %)
7. Psychiatric disorders	n= (n/n, %)	n= (n/n, %)	n= (n/n, %)	n= (n/n, %)	n= (n/n, %)	n= (n/n, %)
8. Nervous system disorders	n= (n/n, %)	n= (n/n, %)	n= (n/n, %)	n= (n/n, %)	n= (n/n, %)	n= (n/n, %)
9. Eye disorders	n= (n/n, %)	n= (n/n, %)	n= (n/n, %)	n= (n/n, %)	n= (n/n, %)	n= (n/n, %)
10. Ear and labyrinth disorders	n= (n/n, %)	n= (n/n, %)	n= (n/n, %)	n= (n/n, %)	n= (n/n, %)	n= (n/n, %)
11. Cardiac disorders	n= (n/n, %)	n= (n/n, %)	n= (n/n, %)	n= (n/n, %)	n= (n/n, %)	n= (n/n, %)
12. Vascular disorders	n= (n/n, %)	n= (n/n, %)	n= (n/n, %)	n= (n/n, %)	n= (n/n, %)	n= (n/n, %)
13. Respiratory, thoracic and mediastinal disorders	n= (n/n, %)	n= (n/n, %)	n= (n/n, %)	n= (n/n, %)	n= (n/n, %)	n= (n/n, %)

14. Gastrointestinal disorders	n= (n/n, %)	n= (n/n, %)	n= (n/n, %)	n= (n/n, %)	n= (n/n, %)	n= (n/n, %)
15. Hepatobiliary disorders	n= (n/n, %)	n= (n/n, %)	n= (n/n, %)	n= (n/n, %)	n= (n/n, %)	n= (n/n, %)
16. Skin and subcutaneous tissue disorders	n= (n/n, %)	n= (n/n, %)	n= (n/n, %)	n= (n/n, %)	n= (n/n, %)	n= (n/n, %)
17. Musculoskeletal and connective tissue disorders	n= (n/n, %)	n= (n/n, %)	n= (n/n, %)	n= (n/n, %)	n= (n/n, %)	n= (n/n, %)
18. Renal and urinary disorders	n= (n/n, %)	n= (n/n, %)	n= (n/n, %)	n= (n/n, %)	n= (n/n, %)	n= (n/n, %)
19. Pregnancy, puerperium and perinatal conditions	n= (n/n, %)	n= (n/n, %)	n= (n/n, %)	n= (n/n, %)	n= (n/n, %)	n= (n/n, %)
20. Reproductive system and breast disorders	n= (n/n, %)	n= (n/n, %)	n= (n/n, %)	n= (n/n, %)	n= (n/n, %)	n= (n/n, %)
21. Congenital, familial and genetic disorders	n= (n/n, %)	n= (n/n, %)	n= (n/n, %)	n= (n/n, %)	n= (n/n, %)	n= (n/n, %)
22. General disorders and administration site conditions	n= (n/n, %)	n= (n/n, %)	n= (n/n, %)	n= (n/n, %)	n= (n/n, %)	n= (n/n, %)
23. Investigations	n= (n/n, %)	n= (n/n, %)	n= (n/n, %)	n= (n/n, %)	n= (n/n, %)	n= (n/n, %)
24. Injury, poisoning and procedural complications	n= (n/n, %)	n= (n/n, %)	n= (n/n, %)	n= (n/n, %)	n= (n/n, %)	n= (n/n, %)
25. Surgical and medical procedures	n= (n/n, %)	n= (n/n, %)	n= (n/n, %)	n= (n/n, %)	n= (n/n, %)	n= (n/n, %)
26. Social circumstances	n= (n/n, %)	n= (n/n, %)	n= (n/n, %)	n= (n/n, %)	n= (n/n, %)	n= (n/n, %)
27. Product issues	n= (n/n, %)	n= (n/n, %)	n= (n/n, %)	n= (n/n, %)	n= (n/n, %)	n= (n/n, %)
TOTAL EVENTS	n= (n/n, %)	n= (n/n, %)	n= (n/n, %)	n= (n/n, %)	n= (n/n, %)	n= (n/n, %)

Table 10 - All adverse reactions after 24 months follow up

	**Arm A**		**Arm B**		**Total**	
**Body System Code**	**Number of NSARs (n participants, %)**	**Number of SARs (n participants, %)**	**Number of NSARs (n participants, %)**	**Number of SARs (n participants, %)**	**Number of SARs (n participants, %)**	**Number of NSARs (n participants, %)**
1. Infections and infestations	n= (n/n, %)	n= (n/n, %)	n= (n/n, %)	n= (n/n, %)	n= (n/n, %)	n= (n/n, %)
2. Neoplasms benign, malignant and unspecified (incl cysts and polyps)	n= (n/n, %)	n= (n/n, %)	n= (n/n, %)	n= (n/n, %)	n= (n/n, %)	n= (n/n, %)
3. Blood and lymphatic system disorders	n= (n/n, %)	n= (n/n, %)	n= (n/n, %)	n= (n/n, %)	n= (n/n, %)	n= (n/n, %)
4. Immune system disorders	n= (n/n, %)	n= (n/n, %)	n= (n/n, %)	n= (n/n, %)	n= (n/n, %)	n= (n/n, %)
5. Endocrine disorders	n= (n/n, %)	n= (n/n, %)	n= (n/n, %)	n= (n/n, %)	n= (n/n, %)	n= (n/n, %)
6. Metabolism and nutrition disorders	n= (n/n, %)	n= (n/n, %)	n= (n/n, %)	n= (n/n, %)	n= (n/n, %)	n= (n/n, %)
7. Psychiatric disorders	n= (n/n, %)	n= (n/n, %)	n= (n/n, %)	n= (n/n, %)	n= (n/n, %)	n= (n/n, %)
8. Nervous system disorders	n= (n/n, %)	n= (n/n, %)	n= (n/n, %)	n= (n/n, %)	n= (n/n, %)	n= (n/n, %)
9. Eye disorders	n= (n/n, %)	n= (n/n, %)	n= (n/n, %)	n= (n/n, %)	n= (n/n, %)	n= (n/n, %)
10. Ear and labyrinth disorders	n= (n/n, %)	n= (n/n, %)	n= (n/n, %)	n= (n/n, %)	n= (n/n, %)	n= (n/n, %)
11. Cardiac disorders	n= (n/n, %)	n= (n/n, %)	n= (n/n, %)	n= (n/n, %)	n= (n/n, %)	n= (n/n, %)
12. Vascular disorders	n= (n/n, %)	n= (n/n, %)	n= (n/n, %)	n= (n/n, %)	n= (n/n, %)	n= (n/n, %)
13. Respiratory, thoracic and mediastinal disorders	n= (n/n, %)	n= (n/n, %)	n= (n/n, %)	n= (n/n, %)	n= (n/n, %)	n= (n/n, %)

14. Gastrointestinal disorders	n= (n/n, %)	n= (n/n, %)	n= (n/n, %)	n= (n/n, %)	n= (n/n, %)	n= (n/n, %)
15. Hepatobiliary disorders	n= (n/n, %)	n= (n/n, %)	n= (n/n, %)	n= (n/n, %)	n= (n/n, %)	n= (n/n, %)
16. Skin and subcutaneous tissue disorders	n= (n/n, %)	n= (n/n, %)	n= (n/n, %)	n= (n/n, %)	n= (n/n, %)	n= (n/n, %)
17. Musculoskeletal and connective tissue disorders	n= (n/n, %)	n= (n/n, %)	n= (n/n, %)	n= (n/n, %)	n= (n/n, %)	n= (n/n, %)
18. Renal and urinary disorders	n= (n/n, %)	n= (n/n, %)	n= (n/n, %)	n= (n/n, %)	n= (n/n, %)	n= (n/n, %)
19. Pregnancy, puerperium and perinatal conditions	n= (n/n, %)	n= (n/n, %)	n= (n/n, %)	n= (n/n, %)	n= (n/n, %)	n= (n/n, %)
20. Reproductive system and breast disorders	n= (n/n, %)	n= (n/n, %)	n= (n/n, %)	n= (n/n, %)	n= (n/n, %)	n= (n/n, %)
21. Congenital, familial and genetic disorders	n= (n/n, %)	n= (n/n, %)	n= (n/n, %)	n= (n/n, %)	n= (n/n, %)	n= (n/n, %)
22. General disorders and administration site conditions	n= (n/n, %)	n= (n/n, %)	n= (n/n, %)	n= (n/n, %)	n= (n/n, %)	n= (n/n, %)
23. Investigations	n= (n/n, %)	n= (n/n, %)	n= (n/n, %)	n= (n/n, %)	n= (n/n, %)	n= (n/n, %)
24. Injury, poisoning and procedural complications	n= (n/n, %)	n= (n/n, %)	n= (n/n, %)	n= (n/n, %)	n= (n/n, %)	n= (n/n, %)
25. Surgical and medical procedures	n= (n/n, %)	n= (n/n, %)	n= (n/n, %)	n= (n/n, %)	n= (n/n, %)	n= (n/n, %)
26. Social circumstances	n= (n/n, %)	n= (n/n, %)	n= (n/n, %)	n= (n/n, %)	n= (n/n, %)	n= (n/n, %)
27. Product issues	n= (n/n, %)	n= (n/n, %)	n= (n/n, %)	n= (n/n, %)	n= (n/n, %)	n= (n/n, %)
TOTAL EVENTS	n= (n/n, %)	n= (n/n, %)	n= (n/n, %)	n= (n/n, %)	n= (n/n, %)	n= (n/n, %)

Figure 2 – example dotplot of incidence of adverse events and relative risk

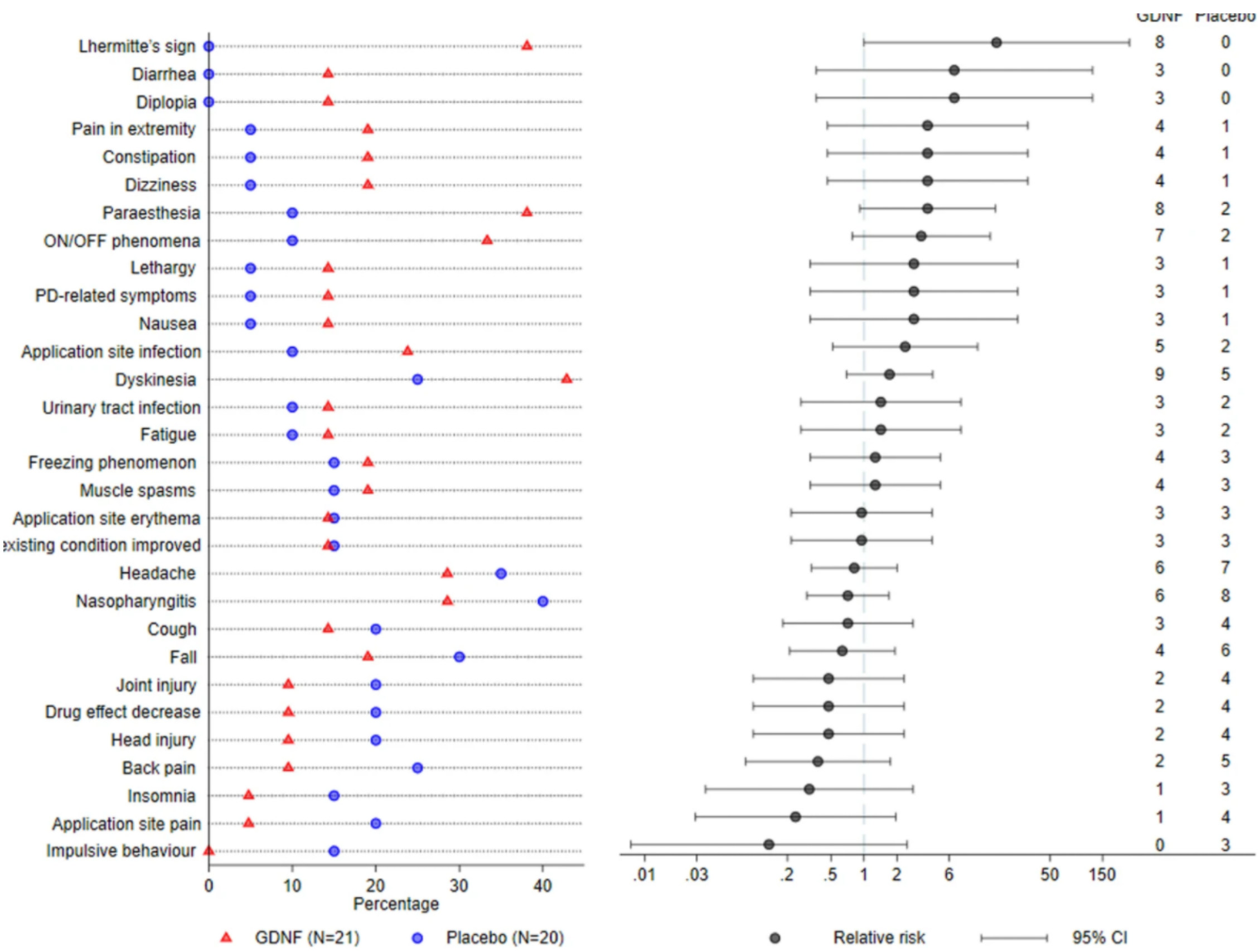

This is an example for purposes of showing formatting only.

Used as per Cornelius et al 2020 (12)

Unblinding events

Table 11 - unblinding events

Unblinding events	FMT	Placebo
Accidental unblinding (n,%)	n, %	n, %
Emergency unblinding (n,%)	n, %	n, %

10 REFERENCE LIST

1. Amit O, Heiberger RM, Lane PW. Graphical approaches to the analysis of safety data from clinical trials. Pharm Stat. 2008;7(1):20–35.

2. Phillips R, Cro S. AEDOT: Stata module to produce dot plot for adverse event data. Statistical Software Components [Internet]. 2021 Nov 17 [cited 2023 Jan 25]; Available from: https://ideas.repec.org//c/boc/bocode/s458735.html

3. Ian R. White, James Carpenter and Nicholas J. Horton (2018). A MEAN SCORE METHOD FOR SENSITIVITY ANALYSIS TO DEPARTURES FROM THE MISSING AT RANDOM ASSUMPTION IN RANDOMISED TRIALS. Vol 28 No. 4, 1985-2003. [Internet]. [cited 2023 Feb 16]. Available from: https://www3.stat.sinica.edu.tw/statistica/J28N4/J28N415/J28N415.html

4. Tsoris A, Marlar CA. Use Of The Child Pugh Score In Liver Disease. In: StatPearls [Internet]. Treasure Island (FL): StatPearls Publishing; 2022 [cited 2023 Feb 16]. Available from: http://www.ncbi.nlm.nih.gov/books/NBK542308/

5. Foxton MR, Al-Freah MAB, Portal AJ, Sizer E, Bernal W, Auzinger G, et al. Increased model for end-stage liver disease score at the time of liver transplant results in prolonged hospitalization and overall intensive care unit costs. Liver Transpl. 2010 May;16(5):668–77.

6. Barber KM, Pioli SE, Blackwell JE, Collett D, Neuberger JM, Gimson AE. Development of a UK score for patients with end-stage liver disease. Hepatology. 2007 Oct;46(4):510A-510A.

7. Herdman M, Gudex C, Lloyd A, Janssen M, Kind P, Parkin D, et al. Development and preliminary testing of the new five-level version of EQ-5D (EQ-5D-5L). Qual Life Res. 2011 Dec;20(10):1727–36.

8. Valuation of EQ-5D – EQ-5D [Internet]. [cited 2023 Jan 25]. Available from: https://euroqol.org/eq-5d-instruments/valuation-of-eq-5d/

9. Saunders JB, Aasland OG, Babor TF, de la Fuente JR, Grant M. Development of the Alcohol Use Disorders Identification Test (AUDIT): WHO Collaborative Project on Early Detection of Persons with Harmful Alcohol Consumption--II. Addiction. 1993 Jun;88(6):791–804.

10. Zigmond AS, Snaith RP. The hospital anxiety and depression scale. Acta Psychiatr Scand. 1983 Jun;67(6):361–70.

11. Morris TP, Jarvis CI, Cragg W, Phillips PPJ, Choodari-Oskooei B, Sydes MR. Proposals on Kaplan–Meier plots in medical research and a survey of stakeholder views: KMunicate. BMJ Open. 2019 Sep 1;9(9):e030215.

12. Cornelius V, Cro S, Phillips R. Advantages of visualisations to evaluate and communicate adverse event information in randomised controlled trials. Trials. 2020 Dec 22;21(1):1028.

13. E 9 Statistical Principles for Clinical Trials. 2006;37.

14. Schulz KF, Altman DG, Moher D, the CONSORT Group. CONSORT 2010 Statement: updated guidelines for reporting parallel group randomised trials. Trials. 2010 Mar 24;11(1):32.

11 AMENDMENTS

V1.1 amendments

- Eligibility criteria updated to:
    - Include definitions of ALD, MASLD and MASLD-ALD overlap.
    - Specify that the exclusion criteria for pregnancy to commence at *screening* rather than *randomisation*.
    - Exclude those who have had surgery which might change gut microbiome.
- Updated cirrhosis diagnosis criteria from Metabolic-Associated Fatty Liver Disease (MAFLD) to Metabolic Dysfunction-Associated Steatotic Liver Disease (MASLD).
- Blood definition of infection updated to: 'exclude common skin contaminants e.g., coagulase-negative Staphylococci or Corynebacteria isolated on a single blood culture set only'.
- New concomitant medications categories now capture new medication type (anti-depressants; anti-anxiety; proton pump inhibitors)
- Template CONSORT diagram (Figure 1) updated to include Day 30 timepoint.
- Removed timepoints Month 9, Month 15, Month 21 from liver disease severity score in the Schedule of Assessment and Measures.
- EQ5d 5l specified to use the Devlin set as will be used to measure quality of life.
- Updated template baseline table to make clear that anxiety and depression are two separate scores, but both taken from the Hospital and Anxiety Depression Scale (HADS).
- Clean up of any typographical, formatting and referencing errors.
- Protocol version number updated.
- Trial Statistician updated under Key Trial Personnel from Rose Tinch-Taylor to Francesca Maher.
- Time-windows for Screening, Baseline, Post-randomisation IMP dispensing updated. Screening and Baseline Repeat schedule added to schedule of measures.
- Timepoints for Urinary β-HCG test, Liver Severity Score (MELD/Child Pugh/UKELD) and Adverse Event Monitoring updated. Liver Profile assessment updated to include MASLD-ALD Overlap Cirrhosis participants. A row for Urinary ethyl glucuronide/Ethyl sulphate test timepoints added in schedule of measures.
- Added section describing the internal pilot (Go, No Go) criteria.

V1.2 Amendments

- Section 4.1.2 'Further details on handling of intercurrent events and subsequent supplementary analyses' was updated to censor liver transplant at the date of transplant. The while-alive strategy will be used for the intercurrent event of liver transplantation, and if there are non-trivial rates of this event (more than 7.5% of participants receive a liver transplant), a composite strategy will be employed.

V2.0 Amendments

A major change made in this amendment was the changing of the primary outcome. The previous primary outcome was time to first infection resulting in hospitalisation. This has been updated to be a composite endpoint of time to first infection or decompensating event. It also now includes events resulting in presentation to the Emergency Department as well as hospitalisation. The rationale behind this change was to increase the low number of primary events that have been observed in the trial so far, whilst still targeting the clinical question. The Trial Management Group (TMG) discussed this with the TSC, who supported the change. The change was approved in the latest protocol amendment (V4.0). The previous primary outcome remains clinically relevant and has been added as a secondary outcome to reflect this and to ensure transparency when reporting.

- Formatting of section headers and Table of Contents updated to be consistent.
- Section 1.2 'Principle research objectives to be addressed' updated to be in line with protocol V4.0.
- Eligibility criteria updated to:
  - MASLD-ALD overlap interchangeable with MetALD
  - Patients with alcohol-related cirrhosis no longer have to be abstinent; instead they must have an active alcohol consumption on average ≤ 20 grams/day, consistent with the criteria for those with MASLD or MetALD
  - Exclude those with a moderate food allergy (previously only excluded those with a severe or life-threatening food allergy)
- Section 1.3 'Endpoints', Section 1.11 'Measures', Section 2.3 'Anticipated intercurrent events', Section 4.1 'Data analysis plan – inferential' updated to reflect the change in primary endpoint definition and to be in line with protocol V4.0. The definition of infection was widened, and the endpoint changed from time to first infection resulting in hospitalisation to a composite endpoint of:
  - Time to infection resulting in hospitalisation or presentation to the emergency department
  - Time to decompensating event resulting in hospitalisation or presentation to the emergency department
- Section 1.3 'Endpoints' was updated to reflect the changes made to the secondary endpoints:
  - The former primary endpoint (time to first infection resulting in hospitalisation) is now a secondary endpoint
  - Clarification of remaining secondary endpoints
- Section 1.11.1 'Measures at baseline only' was updated to be consistent with Section 2.2.
- Section 2.3 'Anticipated intercurrent events' was updated to include significant alcohol intake as an event.
- Section 4.4.1 'Analysis of primary outcomes' was updated to adjust for whether the participant has diabetes in the primary analysis.
- Section 4.5.1 'Continuous outcomes' was updated to include categorical (rather than continuous) time.
- Section 4.5.2 'Binary outcomes' was updated to include categorical (rather than continuous) time and to refine the list of measures included here to be antibiotic usage only.
- Section 4.5.3 'Time to event outcomes' was updated to adjust for diabetes status in line with primary analysis and to add in relevant time-to-event outcome measures in line with the secondary endpoints.

- Section 4.8 'Stratification and clustering' was updated to add that site at randomisation will be adjusted for in the analysis. It was also updated to reflect that secondary outcome (and not primary outcome) data is longitudinal with repeated measurements.
- Section 4.11 'Missing outcome data' was updated to remove the statement about following participants up for their survival status after they have withdrawn from the trial.
- Section 4.15 'Sensitivity analyses' was updated to add a sensitivity analysis excluding participants who drank alcohol (as requested by the TSC).
- Section 7 'Schedule of Assessments and Measures' was updated to add a row for Duration of Abstinence from Alcohol (collected at baseline), and a row for Assessment of Alcohol Intake (collected at months 12, 24 and the primary endpoint).
- The junior trial statistician became unblinded to treatment allocation on 27/03/2025.

PROMISE Statistical Analysis Plan v2.0_08Dec2025

Final Audit Report 2026-02-17

Created:	2026-01-27
By:	KCTU King's Clinical Trials Unit (randomization.request@kcl.ac.uk)
Status:	Signed
Transaction ID:	CBJCHBCAABAAo3bxtTaIHCkS4rssIYmC_DZ9M6CYvLMP

"PROMISE Statistical Analysis Plan v2.0_08Dec2025" History

- Document created by KCTU King's Clinical Trials Unit (randomization.request@kcl.ac.uk)
  2026-01-27 - 10:59:33 GMT- IP address: 193.61.206.5
- Document emailed to Francesca Maher (francesca.maher@kcl.ac.uk) for signature
  2026-01-27 - 11:07:19 GMT
- Email viewed by Francesca Maher (francesca.maher@kcl.ac.uk)
  2026-01-27 - 11:07:33 GMT- IP address: 104.47.51.190
- Document e-signed by Francesca Maher (francesca.maher@kcl.ac.uk)
  Signature Date: 2026-01-27 - 11:07:55 GMT - Time Source: server- IP address: 147.12.151.75
- Document emailed to Ben Carter (ben.carter@kcl.ac.uk) for signature
  2026-01-27 - 11:07:58 GMT
- Email viewed by Ben Carter (ben.carter@kcl.ac.uk)
  2026-01-27 - 11:10:25 GMT- IP address: 104.47.51.190
- Document e-signed by Ben Carter (ben.carter@kcl.ac.uk)
  Signature Date: 2026-01-27 - 11:10:55 GMT - Time Source: server- IP address: 193.61.76.101
- Document emailed to Debbie Shawcross (debbie.shawcross@kcl.ac.uk) for signature
  2026-01-27 - 11:10:58 GMT
- Email viewed by Debbie Shawcross (debbie.shawcross@kcl.ac.uk)
  2026-01-27 - 11:21:28 GMT- IP address: 193.61.74.8
- Document e-signed by Debbie Shawcross (debbie.shawcross@kcl.ac.uk)
  Signature Date: 2026-01-27 - 11:22:02 GMT - Time Source: server- IP address: 193.61.74.8

Document emailed to w.alazawi@qmul.ac.uk for signature

2026-01-27 - 11:22:04 GMT

Email viewed by w.alazawi@qmul.ac.uk

2026-01-27 - 11:59:30 GMT- IP address: 172.226.0.5

Email viewed by w.alazawi@qmul.ac.uk

2026-02-16 - 06:32:05 GMT- IP address: 172.226.0.6

Signer w.alazawi@qmul.ac.uk entered name at signing as William Alazawi

2026-02-17 - 10:48:46 GMT- IP address: 138.37.228.10

Document e-signed by William Alazawi (w.alazawi@qmul.ac.uk)

Signature Date: 2026-02-17 - 10:48:48 GMT - Time Source: server- IP address: 138.37.228.10

Agreement completed.

2026-02-17 - 10:48:48 GMT
